# From netrin‐1‐targeted SPECT/CT to internal radiotherapy for management of advanced solid tumors

**DOI:** 10.15252/emmm.202216732

**Published:** 2023-03-06

**Authors:** David Kryza, Jennifer Wischhusen, Mathieu Richaud, Maëva Hervieu, Jacqueline Sidi Boumedine, Jean‐Guy Delcros, Sophie Besse, Thomas Baudier, Pierre‐Alexandre Laval, Silvia Breusa, Erwan Boutault, Hugo Clermidy, Nicolas Rama, Benjamin Ducarouge, Mojgan Devouassoux‐Shisheboran, Jean‐Michel Chezal, Anne‐Laure Giraudet, Thomas Walter, Patrick Mehlen, David Sarrut, Benjamin Gibert

**Affiliations:** ^1^ Imthernat, LAGEPP, CNRS UMR 5007 Université de Lyon, Hospices Civils de Lyon Lyon France; ^2^ Lumen Nuclear Medicine group, Hospices Civils de Lyon et Centre Léon Bérard Lyon France; ^3^ Apoptosis, Cancer and Development Laboratory‐ Equipe labellisée ‘La Ligue’, LabEx DEVweCAN Institut Convergence PLAsCAN, Centre de Recherche en Cancérologie de Lyon, INSERM U1052‐CNRS 5286, Université de Lyon1 Lyon France; ^4^ Gastroenterology and technologies for health, Centre de Recherche en Cancérologie de Lyon, INSERM U1052‐CNRS5286 Université Lyon 1 Lyon France; ^5^ Small molecules for biological targets, Centre de Recherche en Cancérologie de Lyon. UMR INSERM 1052 – CNRS 5286 ISPB Rockefeller Lyon France; ^6^ Université Clermont Auvergne, Inserm, Imagerie Moléculaire et Stratégies Théranostiques Clermont‐Ferrand France; ^7^ CREATIS, INSA Lyon, INSERM U1206 – CNRS UMR 5220 Université de Lyon Lyon France; ^8^ Netris Pharma Lyon France; ^9^ Hospices Civils de Lyon, Department of Pathology Lyon France; ^10^ Hospices Civils de Lyon, Hôpital Edouard Herriot, Service de Gastroentérologie et d'Oncologie Digestive Lyon Cedex 03 France

**Keywords:** cancer, extracellular matrix, netrin, radioimmunotherapy, targeted therapy, Cancer, Pharmacology & Drug Discovery

## Abstract

Targeted radionuclide therapy is a revolutionary tool for the treatment of highly spread metastatic cancers. Most current approaches rely on the use of vectors to deliver radionuclides to tumor cells, targeting membrane‐bound cancer‐specific moieties. Here, we report the embryonic navigation cue netrin‐1 as an unanticipated target for vectorized radiotherapy. While netrin‐1, known to be re‐expressed in tumoral cells to promote cancer progression, is usually characterized as a diffusible ligand, we demonstrate here that netrin‐1 is actually poorly diffusible and bound to the extracellular matrix. A therapeutic anti‐netrin‐1 monoclonal antibody (NP137) has been preclinically developed and was tested in various clinical trials showing an excellent safety profile. In order to provide a companion test detecting netrin‐1 in solid tumors and allowing the selection of therapy‐eligible patients, we used the clinical‐grade NP137 agent and developed an indium‐111‐NODAGA‐NP137 single photon emission computed tomography (SPECT) contrast agent. NP137‐^111^In provided specific detection of netrin‐1‐positive tumors with an excellent signal‐to‐noise ratio using SPECT/CT imaging in different mouse models. The high specificity and strong affinity of NP137 paved the way for the generation of lutetium‐177‐DOTA‐NP137, a novel vectorized radiotherapy, which specifically accumulated in netrin‐1‐positive tumors. We demonstrate here, using tumor cell‐engrafted mouse models and a genetically engineered mouse model, that a single systemic injection of NP137‐^177^Lu provides important antitumor effects and prolonged mouse survival. Together, these data support the view that NP137‐^111^In and NP137‐^177^Lu may represent original and unexplored imaging and therapeutic tools against advanced solid cancers.

## Graphical abstract

During development, netrin‐1 is released and diffusible in the microenvironment. We showed that in cancer, netrin‐1 accumulates in the extracellular matrix. We used this property to follow its *in vivo* accumulation by radiolabeling and to target netrin‐1‐expressing tumor cells with radioimmunotherapy.

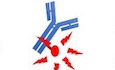

## Introduction

Netrin‐1 is known to be crucial during embryonic development and more particularly in the establishment of the central nervous system, through its role in neuronal navigation as exemplified in commissural axon guidance (Serafini *et al*, 1996; Belle *et al*, 2014). The classically admitted view of its role was that it attracts/repulses neurons/axons following its secretion through the generation of a gradient in the surrounding environment. This has recently been questioned as netrin‐1 seems to be less diffusible than initially described but rather actively transported during embryonic development (Dominici *et al*, 2017). Its signaling is transduced by dependence receptors (DRs) like deleted in colorectal carcinoma (DCC), members of the uncoordinated‐5 (UNC‐5) family, and neogenin (Fazeli *et al*, 1997; Mehlen *et al*, 2011; Renders *et al*, 2021). Binding of netrin‐1 to these receptors impedes cell death and explains its broad physiological and pathological implications (van Gils *et al*, 2012; Ramkhelawon *et al*, 2014; Ozmadenci *et al*, 2015; Boneschansker *et al*, 2016; Brisset *et al*, 2021).

In particular, netrin‐1 was shown to be upregulated in many types of cancers, including breast cancer, non‐small‐cell lung cancer, and medulloblastoma (Fitamant *et al*, 2008; Delloye‐Bourgeois *et al*, 2009; Akino *et al*, 2014). This overexpression in tumor cells is one of the survival mechanisms proposed, whereby increased expression of netrin‐1 blocks cell death induced by DRs (Mehlen *et al*, 2011; Gibert & Mehlen, 2015), and seems to be enhanced following chemotherapy (Paradisi *et al*, 2013). Targeting this DR/ligand interaction has thus appeared as an attractive therapeutic strategy in oncology (Paradisi *et al*, 2013; Gibert & Mehlen, 2015; Grandin *et al*, 2016a,b), with several molecules under development to block netrin‐1. Consistently, a human monoclonal anti‐netrin‐1 antibody, called NP137, blocking UNC5B/netrin‐1 interaction, is currently assessed in several clinical Phase II trials to evaluate its anticancer activity in monotherapy or in combination with standards of care such as chemotherapy or immune checkpoint inhibitors. Preliminary results of clinical activity as a single agent are encouraging (Grandin *et al*, 2016b; Cassier *et al*, 2019). In addition, netrin‐1 blockage seems to be effective in a subset of patients only requiring a simple test to identify eligible patients, that is, those with detectable netrin‐1 levels. Conventional approaches to identify patients likely to respond to cancer treatments are largely based on invasive primary tissue biopsy collection and histological staining and analysis, and overlook specific features of metastases, such as initial treatment resistance. Indeed, immunohistochemistry (IHC) has been the reference for the characterization of target expression in cancer for many years. However, this strategy has been called into question by recent data obtained with immune checkpoint inhibitors, as there is a strong discrepancy between target expression and response within the patient. Thus, patients responding to the PDL‐1 antibody could be negative for the expression of PDL‐1 in IHC and *vice versa*. It can be hypothesized that target expression is not stable over time; for instance, since IHC analyses are made with paraffin blocks taken from the primary tumor at diagnosis sometimes months or years before clinical trials. The expression of the target may then differ in metastases. New diagnostic strategies are therefore needed to analyze target expression in real time on a whole‐body scale to highlight all the variations in protein expression within tumors and metastases.

In an attempt to develop a less invasive means of detection/diagnosis of netrin‐1‐expressing patients eligible for anti‐netrin‐1 treatment, we developed a radiolabeling‐based methodology to visualize netrin‐1 accumulation *in vivo* by SPECT/CT imaging. We then demonstrated netrin‐1 accumulation in the tumor cell extracellular matrix rather than its diffusion in the microenvironment and detected a strong accumulation of radioactivity within tumors. We then created a new radiolabeled NP137 compound aimed at internally delivering radiopharmaceutical therapy (RPT), able to selectively kill netrin‐1‐expressing cells with radioactivity through the highly specific accumulation of the NP137 agent. To achieve this, we radiolabeled NP137 with lutetium‐177 (^177^Lu) and observed tumor cell death with high specificity in a number of xenografts and genetic mouse models. This technology based on coupled peptides and antibody drug conjugates is already used in the clinic (Strosberg *et al*, 2017; Sartor *et al*, 2021) and many developments are ongoing to target both cancer cells and the tumor microenvironment that surrounds them, such as activated platelets or cancer‐associated fibroblasts (CAFs) (Dal Corso *et al*, 2017; Lindner *et al*, 2019; Yap *et al*, 2019; Fabre *et al*, 2020).

Taken together, these data demonstrate that our radiolabeled tools can provide very effective targeting of tumor cells and are highly relevant for a rapid transfer to the clinic.

## Results

### Netrin‐1 is a poorly diffusible matrix‐binding protein

**Figure 1:**
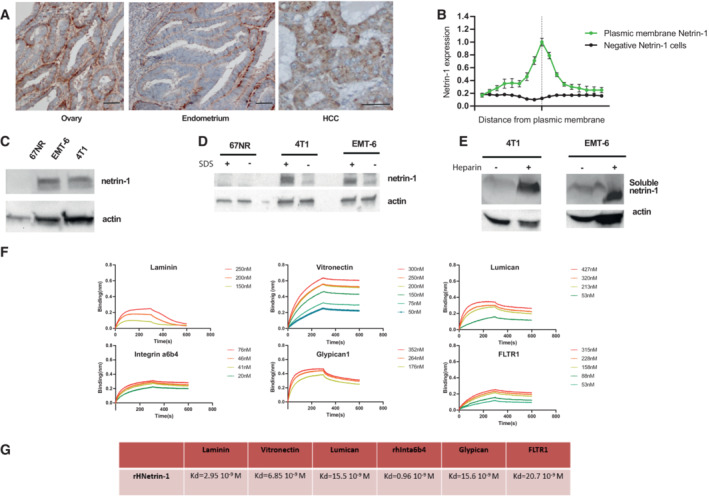
Netrin‐1 binds to and is retained within the extracellular matrix (ECM) ARepresentative bright field micrographs of netrin‐1 immunohistochemistry in formalin‐fixed paraffin‐embedded human ovarian, endometrial, and hepatocellular carcinoma (HCC) tumor sections. Scale bar 50 μm.BQuantification by immunoblots of netrin‐1 in the ECM of cancer cells of human tumors (*n* = 3, error bars are s.d.). The vertical line corresponds to the intercellular zone.CSelection of models expressing netrin‐1 by immunoblot.DImmunoblots quantification of netrin‐1 in samples harvested with or without SDS lysis buffer.EQuantification by immunoblots of netrin‐1 in concentrated supernatants from cells treated or not with heparin.FAnalysis of h‐netrin‐1 (human recombinant netrin‐1) binding to ECM components: mouse laminin‐I (m‐Laminin‐I), recombinant human vitronectin, recombinant human lumican, recombinant human integrin α6β4, recombinant human glypican‐1, and recombinant human FLTR1 by biolayer interferometry assays.GK_d_ calculation after biolayer interferometry analysis of the experiment presented in (F). Source data are available online for this figure.

In order to follow netrin‐1 by radio‐imaging *in vivo*, we first had to precisely localize the protein. Indeed, recent studies suggested that netrin‐1 may not be as diffusible as originally thought during embryonic development (Serafini *et al*, 1996; Dominici *et al*, 2017). To our knowledge, no one has addressed this issue in pathological conditions and more specifically in cancer. IHC analysis of netrin‐1 levels in human tumors showed that the protein was present in the basement membrane and in the intercellular space of tumor cells, indicative of its accumulation in the extracellular matrix (Fig 1A and B). To further investigate the level of netrin‐1 diffusion, we conducted immunoblots of cells expressing netrin‐1 (4T1 and EMT6 cell lines) or not (67NR) collected from plastic dishes with or without SDS, capable of solubilizing matrix proteins. Netrin‐1 could be clearly measured in detergent conditions, in which it is detached from the matrix, compared with control cells, indicating that it may bind to the matrix (Fig 1C and D). We thus used heparin treatment, a procedure routinely used to separate matrix and sugar residues from cells growing in plastic plates (Serafini *et al*, 1994). While no netrin‐1 was detected in the conditioned medium from netrin‐1‐expressing 4T1/EMT6 cells in non‐heparin‐treated condition, when heparin was added, netrin‐1 was detected (Fig 1E). Together, these data support the view that netrin‐1 is only poorly diffusible and binds to the matrix. In order to study the molecular mechanisms and protein interactions necessary for the binding of netrin‐1 to the matrix, we sought to identify potential molecular partners of netrin‐1 within the matrix components, by conducting a biolayer interferometry (BLI) screen (Fig 1F). Human fibronectin was used as a negative control. Of interest, we identified that netrin‐1 strongly bound to the matrix‐forming proteins laminin‐I (K_d_ = 29.5 nM), lumican (K_d_ = 15.5 nM), fibronectin leucine‐rich transmembrane protein (FLRT1) (K_d_ = 15.5 nM), integrin α6β4 (K_d_ = 0.96 nM), glypican‐1 (K_d_ = 15.6 nM), and vitronectin (K_d_ = 6.85 nM; Fig 1F and G).

Together, these results argue in favor of netrin‐1 sequestration by the matrix rather than its diffusion in the cell microenvironment. Netrin‐1 being immobilized in the extracellular matrix of tumor cells, we hypothesized that netrin‐1 could be detected by *in vivo* molecular imaging. Thus, we aimed at developing a netrin‐1‐targeted radiotracer for nuclear imaging.

### Characterization of a new companion test for real‐time netrin‐1 detection

**Figure 2:**
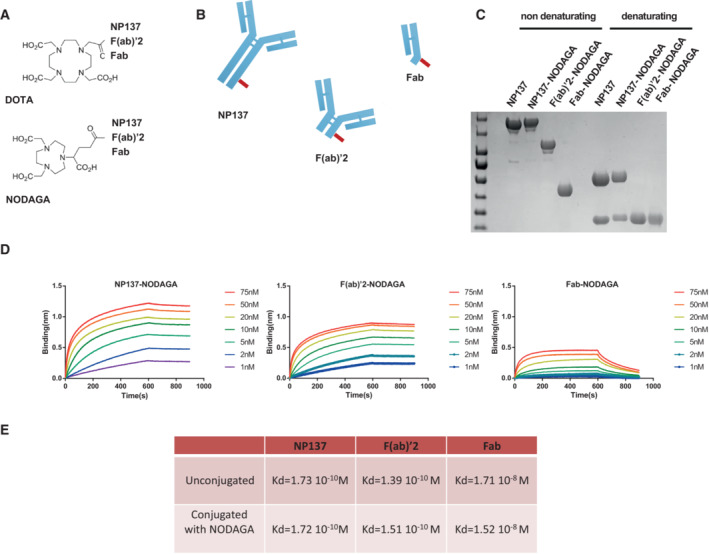
Characterization of conjugated NP137 fragments AChemical representation of DOTA (1,4,7,10‐tetraazacyclododecane‐1,4,7,10‐tetraacetic acid) and NODAGA (1,4,7‐triazacyclononane, 1‐glutaric acid‐4,7 acetic acid) immunoconjugates.BRepresentation of full anti‐netrin‐1 (NP137), F(ab′)_2_, and Fab conjugated to NODAGA or DOTA chelators (in red).CNP137 fragments were produced by enzymatic cleavage and subjected to electrophoresis in denaturing or nondenaturing conditions.DBiolayer interferometry analysis of netrin‐1/NP137 and derivates after NODAGA bioconjugation. Numbers indicate the concentration in nM of NP137 and derivates.EK_d_ calculation after biolayer interferometry analysis of the experiments presented in d and compared with K_d_ of nonmodified NP137. Source data are available online for this figure.

**Figure EV1:**
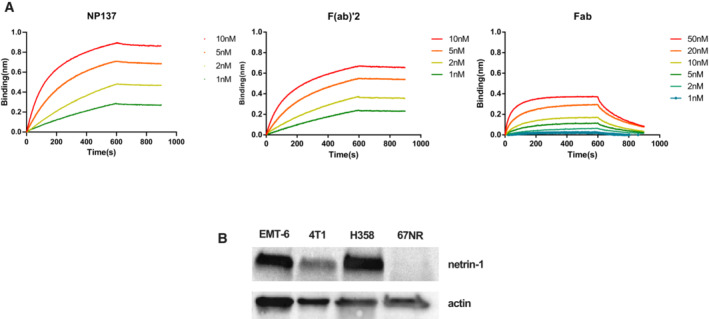
NP137 fragments analysis and netrin‐1 expression in tumor cell models ABiolayer interferometry kinetic analysis of NP137 and its F(ab′)_2_ and Fab fragments on netrin‐1. Colors indicate the concentration of NP137 and derivates.BQuantification by immunoblots of netrin‐1 expression in the cell lines used in the study. Source data are available online for this figure.

Personalized medicine approaches rely on biomarker assessment to guide decision‐making for effective cancer patient management. Accordingly, we assumed that netrin‐1 protein levels would be key to selecting patients eligible for anti‐netrin‐1 treatment. To develop a real‐time noninvasive companion test to specifically monitor netrin‐1 expression, we prepared novel radiotracers by conjugating NP137 or fragments of it (Fab or F(ab′)_2_) to metal chelators (DOTA or NODAGA; Fig 2A–C), which in turn allowed for loading with radioactive indium‐111 (^111^In, half‐life of 67 h), routinely used in nuclear imaging and detectable by SPECT imaging (Ritt, 2022). The three molecules ^111^In‐NP137, ^111^In‐NP137‐Fab, and ^111^In‐NP137‐F(ab′)_2_ were designed to assess their diffusion and accumulation properties *in vivo*. The full‐size ^111^In‐NP137 was presumed to have a longer *in vivo* half‐life, ^111^In‐NODAGA‐NP137‐Fab to penetrate the tumor more rapidly, and ^111^In‐NODAGA‐NP137‐F(ab′)_2_ to penetrate faster than the full‐size agent and with better retention than the Fab construct due to better affinity and avidity. We first verified by BLI that the conjugation of the metal chelator did not disturb the ability of NP137, NP137‐F(ab′)_2_, and NP137‐Fab to bind netrin‐1 and to inhibit the binding of netrin‐1 to its receptors (Fig 2D). We observed that the chelator did not alter the affinity of the full‐size antibody or its fragments, as evidenced by a K_d_ in the nanomolar range (Figs 2D and E, and EV1A and B).

**Figure 3:**
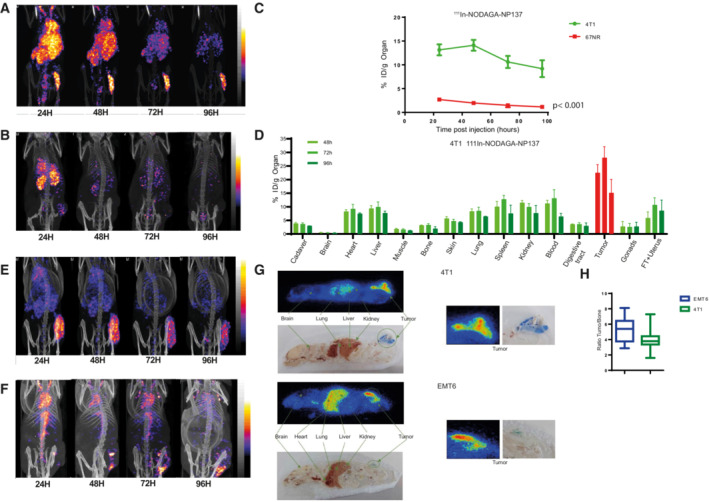
A new SPECT/CT companion test for netrin‐1 detection AMaximum Intensity Projection of Tomographic scintigraphy and X‐ray CT of the whole body of a Balb/cJ mouse bearing a 4T1(netrin‐1‐positive) tumor, acquired 24, 48, 72, and 96 h after IV injection of NP137‐NODAGA‐^111^In.BMaximum Intensity Projection of Tomographic scintigraphy and X‐ray CT of the whole body of a Balb/c mouse bearing a 67NR (netrin‐1‐negative) tumor, acquired 24, 48, 72, and 96 h after IV injection of NP137‐NODAGA‐^111^In.CTumor biodistribution ratio of NP137‐NODAGA‐^111^In in Balb/cJ mice bearing 4T1 xenograft versus 67NR xenografts at 24 h (4 mice), 48 h (4 mice), 72 h (4 mice) and 96 h (4 mice); Two‐way ANOVA; Error bars indicate s.d.DBiodistribution properties of NP137‐NODAGA‐^111^In in a Balb/cJ mouse bearing 4T1 xenografts at 48 h (4 mice), 72 h (4 mice), and 96 h (3 mice). Radioactivity incorporation was quantified by the percentage of the injected dose *per* gram of organ. Error bars indicate s.d.EMaximum Intensity Projection of Tomographic scintigraphy and X‐ray CT of the whole body of a Balb/cJ mouse bearing an EMT6 (netrin‐1‐positive) tumor, acquired 24, 48, 72, and 96 h after IV injection of NP137‐NODAGA‐^111^In.FMaximum Intensity Projection of Tomographic scintigraphy and X‐ray CT of the whole body of a NMRI nude mouse bearing a H358 (netrin‐1‐positive) tumor, acquired at 24, 48, 72, and 96 h after IV injection of NP137‐NODAGA‐^111^In.GWhole‐body autoradiography of NP137‐NODAGA‐^111^In in Balb/cJ mice bearing a 4T1 (top) or an EMT6 (bottom) tumor using a beta imager system.HAnalysis of the autoradiography ratio of NP137‐NODAGA‐^111^In in tumor versus bone in Balb/cJ mice bearing EMT6 (*n* = 14 relative values) or 4T1 (*n* = 24 relative values) tumors. Whiskers indicate min to max, Central band of the box indicates the median, bottom of the box indicates Q1, and top of the box indicates Q3. Source data are available online for this figure.

**Figure EV2:**
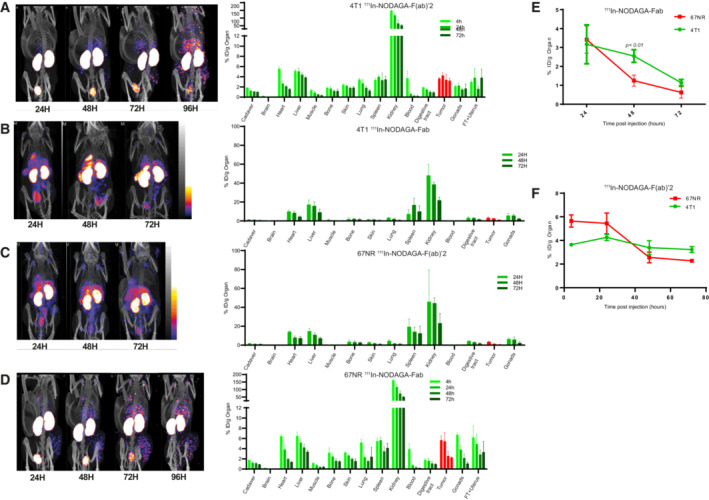
*In vivo* detection of netrin‐1 using NP137‐Fab or NP‐137‐F(ab')_2_ constructs A(Left) Maximum Intensity Projection of Tomographic scintigraphy and X‐ray CT of the whole body of a Balb/c mouse bearing a 4T1 tumor (netrin‐1‐negative), acquired 24, 48h, 72, and 96 h after IV injection of Fab‐NODAGA‐^111^In. (Right) Biodistribution properties of Fab‐NODAGA‐^111^In in Balb/cJ mouse bearing 4T1 xenografts at 4 h (3 mice), 24 h (4 mice), 48 h (4 mice), and 72 h (4 mice) and measured for all organs. Radioactivity incorporation was quantified by the percentage of the injected dose per gram of organ. Error bars indicate s.d.B(Left) Maximum Intensity Projection of Tomographic scintigraphy and X‐ray CT of the whole body of a Balb/c mouse bearing a 4T1 (netrin‐1‐positive) tumor, acquired 4, 24, 48, and 72 h after IV injection of F(ab)′_2_‐NODAGA‐^111^In. (Right) Biodistribution properties of F(ab′)_2_ ‐NODAGA‐^111^In in a Balb/cJ mouse bearing 4T1 xenografts at 4 h (5 mice), 24 h (5 mice), 48 h (5 mice), and 72 h (5 mice) and measured in all organs. Radioactivity incorporation was quantified by the percentage of the injected dose *per* gram of organ. Error bars indicate s.d.C(Left) Maximum Intensity Projection of Tomographic scintigraphy and X‐ray CT of the whole body of a Balb/c mouse bearing a 67NR tumor (netrin‐1‐negative), acquired 24, 48, and 72 h after IV injection of F(ab′)_2_‐NODAGA‐^111^In. (Right) Biodistribution properties of F(ab′)_2_‐NODAGA‐^111^In in a Balb/cJ mouse bearing 67NR xenografts at 24 h (3 mice), 48 h (4 mice), and 72 h (4 mice) and measured for all organs. Radioactivity incorporation was quantified by the percentage of the injected dose *per* gram of organ. Error bars indicate s.d.D(Left) Maximum Intensity Projection of Tomographic scintigraphy and X‐ray CT of the whole body of a Balb/c mouse bearing a 67NR (netrin‐1‐negative) tumor, acquired 24, 48, and 72 h after IV injection of Fab‐NODAGA‐^111^In. (Right) Biodistribution properties of Fab‐NODAGA‐^111^In in a Balb/cJ mouse bearing 67NR xenografts at 4 h (3 mice), 24 h (4 mice), 48 h (4 mice), and 72 h (3 mice) and measured in all organs. Radioactivity incorporation was quantified by the percentage of the injected dose per gram of organ. Error bars indicate s.d.ETumor biodistribution ratio of Fab‐NODAGA‐^111^In in Balb/cJ mice bearing 4T1 xenografts versus 67NR xenografts at 4 h (5 4T1 mice and 3 67NR mice), 24 h (5 4T1 mice and 4 67NR mice), 48 h (5 4T1 mice and 4 67NR mice), and 72 h (5 4T1 mice and 4 67NR mice); Two‐way ANOVA. Error bars indicate s.d.FTumor biodistribution ratio of F(ab′)_2_‐NODAGA‐^111^In in Balb/cJ mice bearing 4T1 xenografts versus 67NR xenografts at 4 h (3 mice), 24 h (4 mice), 48 h (4 mice), and 72 h (4 mice); Two‐way ANOVA; Error bars indicate s.d. Source data are available online for this figure.

Next, to analyze the capacity of these molecules to detect netrin‐1 *in vivo*, we engrafted netrin‐1‐positive 4T1 cells and netrin‐1‐negative 67NR cells in syngeneic mice. Engrafted animals were injected with radiolabeled immunoconjugates, and radioactivity uptake was monitored by SPECT/CT imaging. A rapid ^111^In‐NODAGA‐NP137 uptake was detectable in 4T1 tumors, whereas that of ^111^In‐NODAGA‐NP137‐F(ab′)_2_ was slower, and that of ^111^In‐NODAGA‐Fab negligible (Figs 3A and EV2A and B). Interestingly, no enhancement of radioactive signal was detected in 67NR tumors, indicating that uptake was specific to netrin‐1‐expressing tumors (Figs 3B and EV2C and D). The incorporation ratio of radioactivity in 67NR versus 4T1 tumor cells was determined over time, and an accumulation of 14.1% of total injected radioactivity was measured in netrin‐1‐positive tumors at 48 h compared with 1.97% in netrin‐1‐negative tumors (*P* < 0.001; Fig 3C). To detect possible nonspecific binding of NP137, we quantified indium‐111 uptake within organs, following mouse sacrifice and organ removal. We observed a peak in the incorporation of 28% of the injected dose/g of organ 72 h post injection in netrin‐1‐positive tumors, other tissues displaying minimal radioactive signal enhancement, highlighting the specificity of ^111^In‐NODAGA‐NP137 (Fig 3D). Taken together, these data demonstrate that tumoral indium‐111 accumulation is more efficient with the full‐size antibody compared with the Fab or F(ab′)_2_ constructs (Figs 3C and EV2E and F).

**Figure EV3:**
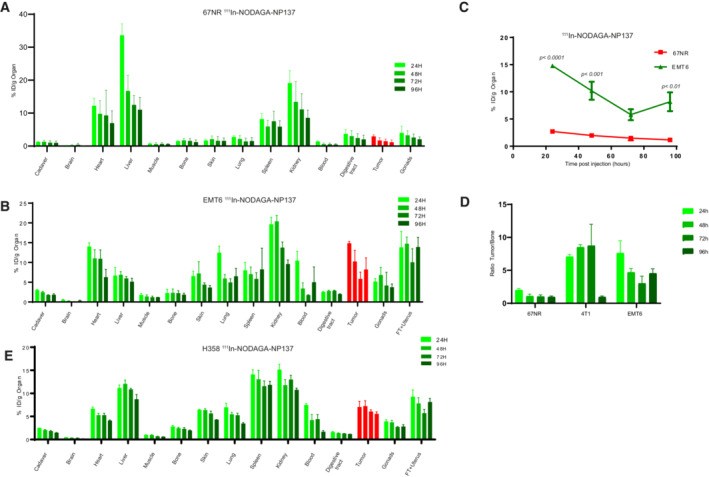
Biodistribution analysis of radiolabeled NP137 ABiodistribution properties of NP137‐NODAGA‐^111^In in a Balb/cJ mouse bearing 67NR xenografts at 24 h (3 mice), 48 h (4 mice), 72 h (4 mice), and 96 h (4 mice) and measured for all organs. Radioactivity incorporation was quantified by the percentage of the injected dose per gram of organ. Error bars indicate s.d.BBiodistribution properties of NP137‐NODAGA‐^111^In in a Balb/cJ mouse bearing EMT6 xenografts at 24 h (3 mice), 48 h (3 mice), 72 h (3 mice), and 96 h (3 mice) and measured in all organs. Radioactivity incorporation was quantified by the percentage of the injected dose *per* gram of organ. Error bars indicate s.d.CTumor biodistribution ratio of NP137‐NODAGA‐^111^In in Balb/cJ mice bearing EMT6 xenografts versus 67NR xenografts at 24 h (3 mice), 48 h (4 67NR mice and 3 EMT6 mice), 72 h (4 67NR mice and 3 EMT6 mice), and 96 h (4 67NR mice and 3 EMT6 mmice); Two‐way ANOVA. Error bars indicate s.e.m.DRatio of tumor versus bone with the percentage of injected dose per gram of organ of NP137‐NODAGA‐^111^In in Balb/cJ mice bearing 67NR, 4T1 or EMT6 xenografts at 24 h (5 67NR mice, 4 4T1 mice, and 3 EMT6 mice), 48 h (5 67NR mice, 4 4T1 mice, and 3 EMT6 mice), 72 h (5 67NR mice, 3 4T1 mice, and 3 EMT6 mice), and 96 h (4 67NR mice, 4 4T1 mice, and 3 EMT6 mice). Error bars indicate s.e.m.ETumor biodistribution profile of NP137‐NODAGA‐^111^In in NMRI Nude mice bearing H358 tumors at 24 h (5 mice), 48 h (5 mice), 72 h (5 mice), and 96 h (4mice). Error bars indicate s.d. Source data are available online for this figure.

Finally, we confirmed the accumulation of NP137‐^111^In in another mouse tumor model, EMT6, and in a human xenograft model H358 both expressing netrin‐1 at different levels with tumoral uptakes in both models (Figs 3E and F, and EV3A–E). The specific accumulation of NP137 within tumors was further assessed by *ex vivo* autoradiography showing peaks of signal within 4T1 and EMT6 tumor models and corroborating the *in vivo* imaging results (Fig 3G). To verify the level of toxicity of NP137‐^111^In compound, we also analyzed the ratio of radioactivity measured between the tumor and bone, which is the niche of hematopoiesis and a prime location for the sequestration of drugs in patients, and observed a 6‐ and 4‐fold higher radioactivity capture in EMT6 and 4T1 tumors, respectively, than in the bone suggesting minimal off‐target accumulation (Fig 3H).

### NP137‐^177^Lu as a new theranostic compound to target aggressive tumors

**Figure 4:**
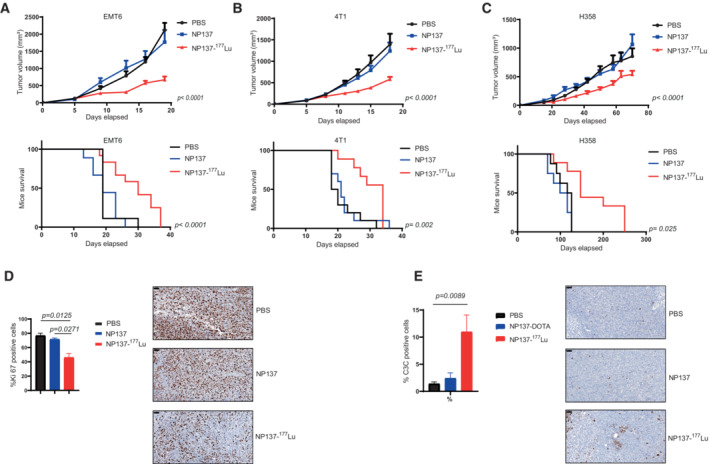
A new radiolabeled NP137‐based anticancer therapy A(Top) Balb/cJ mice were engrafted with EMT6 cells by subcutaneous injection of 1 million cells. After 5 days, animals were treated by IV injection of PBS, DOTA‐NP137, or NP137‐^177^Lu. *n* = 9 animals/group for PBS and DOTA‐NP137; *n* = 12 animals/group for NP137‐^177^Lu; *P* < 0.001 between PBS and NP137‐^177^Lu and between DOTA‐NP137 and NP137‐^177^Lu (Two‐way ANOVA) between PBS and NP137‐^177^Lu and between DOTA‐NP137 and NP137‐^177^Lu. Error bars indicate s.e.m. (Bottom) NP137‐^177^Lu survival of mice engrafted with EMT6 cells. Kaplan–Meier survival curves of mice treated or not with DOTA‐NP137. Mantel–Cox test; *n* = 9 animals/group for PBS and DOTA‐NP137; *n* = 12 animals/group considered as dead at a tumor volume of 2,000 mm^3^ for NP137‐^177^Lu; *P* < 0.001 between PBS and NP137‐^177^Lu and between DOTA‐NP137 and NP137‐^177^Lu.B(Top) Balb/c mice were engrafted with 4T1 cells by subcutaneous injection of 1 million cells. After 8 days, animals received IV injections of PBS, DOTA‐NP137, or NP137‐^177^Lu; *n* = 10 animals/group for PBS and DOTA‐NP137; *n* = 9 animals/group for NP137‐^177^Lu; *P* < 0.001 between PBS and NP137‐^177^Lu and between DOTA‐NP137 and NP137‐^177^Lu (Two‐way ANOVA). Error bars indicate s.e.m. (Bottom) NP137‐^177^Lu survival of mice engrafted with 4T1 cells. Kaplan–Meier survival curves of mice treated or not with DOTA‐NP137. Mantel–Cox test; *n* = 10 animals/group for PBS and DOTA‐NP137; *n* = 9 animals/group considered as dead at a tumor volume of 2,000 mm^3^ for NP137‐^177^Lu; *P* = 0.002 between PBS and NP137‐^177^Lu and between DOTA‐NP137 and NP137‐^177^Lu.C(Top) NMRI nude mice were engrafted with H358 cells by subcutaneous injection of 5 million cells and considered as dead when tumor volume reached 2,000 mm^3^. After 15 days, animals were treated by IV injection of PBS, DOTA‐NP137, or NP137‐^177^Lu; *n* = 8 animals/group for PBS and DOTA‐NP137; *n* = 9 animals/group for NP137‐^177^Lu; *P* < 0.001 between PBS and NP137‐^177^Lu and between DOTA‐NP137 and NP137‐^177^Lu (2‐way ANOVA) between PBS and NP137‐^177^Lu and between DOTA‐NP137 and NP137‐^177^Lu. Error bars indicate s.e.m. (Bottom) NP137‐^177^Lu survival of mice engrafted with H358 cells. Kaplan–Meier survival curves of mice treated or not with DOTA‐NP137. Mantel–Cox test; *n* = 8 animals/group for PBS and DOTA‐NP137; *n* = 9 animals/group for NP137‐^177^Lu; *P* = 0.025 between PBS and NP137‐^177^Lu and between DOTA‐NP137 and NP137‐^177^Lu.D(Left) Quantification of Ki67 expression in EMT6 tumors harvested in Balb/cJ mice 7 days post injection of PBS, DOTA‐NP137, or NP137‐^177^Lu. (Right) Representative bright field micrographs of Ki67 immunohistochemistry in formalin‐fixed paraffin‐embedded EMT6 tumors harvested in Balb/cJ mice 7 days post injection of PBS, DOTA‐NP137, or NP137‐^177^Lu; *n* = 4 mice per groups; One‐way ANOVA. Error bars indicate s.e.m. Scale bar: 50 μm.E(Left) Quantification of cleaved caspase‐3 expression in EMT6 tumors harvested in Balb/cJ mice 7 days post injection of PBS, DOTA‐NP137, or NP137‐^177^Lu. (Right) Representative bright field micrographs of cleaved caspase‐3 immunohistochemistry in formalin‐fixed paraffin‐embedded EMT6 tumor harvested in Balb/cJ mice 7 days post injection of PBS, DOTA‐NP137, or NP137‐^177^Lu; *n* = 4 mice *per* groups; One‐way ANOVA. Error bars indicate s.e.m. Scale bar: 50 μm. Source data are available online for this figure.

**Figure EV4:**
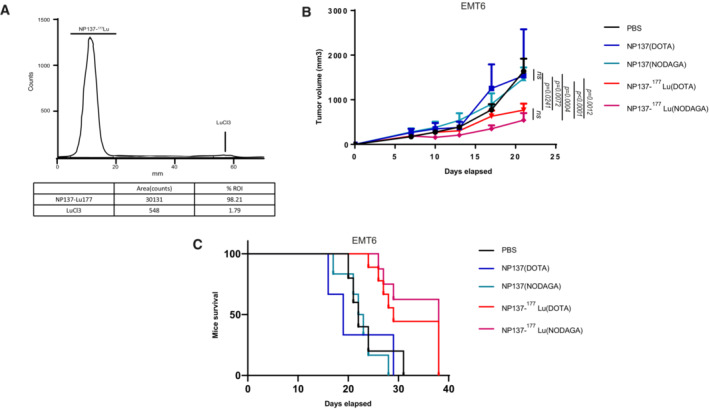
Analysis of chelation of ^177^Lu with DOTA or NODAGA ARadiochemical purity evaluation of NP137‐^177^Lu incubated 7 days in human plasma samples.BBalb/cJ mice were engrafted with EMT6 cells by subcutaneous injection of 1 million cells. After 5 days, animals were treated by IV injection of PBS; DOTA‐NP137; NODAGA‐NP137; DOTA‐NP137‐^177^Lu; or NODAGA‐NP137‐^177^Lu. *n* = 5 animals/group for PBS; *n* = 3 animals/group for DOTA‐NP137; *n* = 6 animals/group for NODAGA‐NP137; *n* = 9 animals/group for DOTA‐NP137‐^177^Lu; *n* = 7 animals/group for NODAGA‐NP137‐^177^Lu. *P* = 0.0241 between DOTA‐NP137 and DOTA‐NP137‐^177^Lu.; *P* < 0.001 between NODAGA‐NP137 and NODAGA‐NP137‐^177^Lu. Turkey's multiple comparison test. Error bars indicate s.e.m.CNo difference in survival between DOTA‐NP137‐^177^Lu and NODAGA‐NP137‐^177^Lu in mice engrafted with EMT6. Kaplan–Meier survival curves of mice treated or not with NP137. Mantel–Cox test; *n* = 5 animals/group for PBS; *n* = 3 animals/group for DOTA‐NP137; *n* = 6 animals/group for NODAGA‐NP137; *n* = 9 animals/group for DOTA‐NP137‐^177^Lu; *n* = 7 animals/group for NODAGA‐NP137‐^177^Lu. Source data are available online for this figure.

Based on our findings on ^111^In‐radiolabeled NP137 uptake by tumors, we wondered whether the antibody could be coupled to a therapeutic agent. Indeed, even though NP137 has shown encouraging antitumoral results in clinical settings, imaging data shown here supported the use of NP137 as a potential vector to deliver radiotherapy. Given that the incorporation of the antibody into these tumors was rapid, specific, and that it accumulated in the extracellular matrix (ECM) of these cells, we developed a radiolabeled molecule comprising the anti‐netrin‐1 antibody NP137 fused to the radionuclide lutetium‐177. NP137‐DOTA‐^177^Lu (or thereafter NP137‐^177^Lu), that emits beta radiation (half‐life of 6.7 days), was shown to damage/kill cancer cells in prostate and neuroendocrine tumors (Tagawa *et al*, 2013; Strosberg *et al*, 2017). We observed that ^177^Lu binding to the anti‐netrin‐1 mAb does not alter the stability of the antibody via a radiolysis phenomenon. NP137‐^177^Lu was stable for over 7 days in a human plasma sample without the release of potentially toxic metal ions (Fig EV4A). To analyze the tumor growth inhibitory capacity of this compound, we treated mice engrafted with 4T1 and EMT6 tumors, using a single dose of NP137‐DOTA, as a control, or NP137‐^177^Lu. Single injection of NP137‐^177^Lu (< 10 MBq/mouse) significantly decreased tumor growth associated with a two‐fold increase in mouse disease‐free survival (*P* < 0.001; Fig 4A and B). Comparison of the chelators DOTA or NODAGA for loading of lutetium‐177 and their effect on NP137‐^177^Lu‐mediated tumor growth inhibition and survival did not show any difference suggesting that both chelators had an equal performance (Fig EV4B and C). Therapeutic efficacy of NP137‐^177^Lu was further assessed in the human lung cancer xenograft model H358 where, once again, the treatment significantly reduced the rate of tumor growth corroborating a clear antitumoral effect (*P* < 0.001; Fig 4C). The reduction in tumor growth was shown to be mediated by an increase in tumor cell apoptosis, as detected by caspase‐3 cleavage, and a decrease in tumor proliferation, as detected by Ki67 staining (Fig 4D and E).

### Radiolabeled NP137 as new diagnostic tools for early tumor detection and treatment of a transgenic cancer model

**Figure 5:**
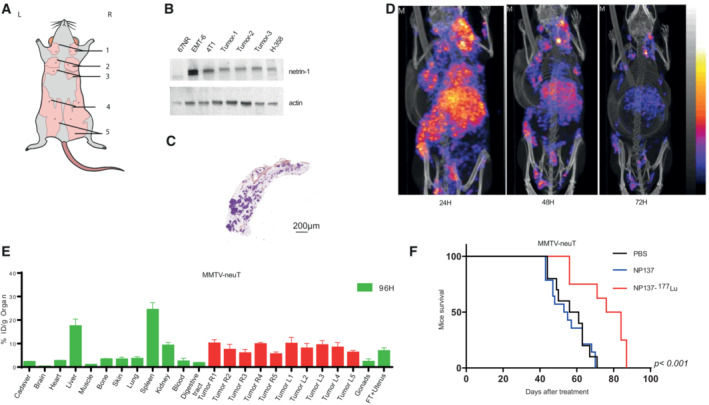
Detection of early tumor lesions and treatment of physiological tumors ASchematic representation and location of the 10 mammary glands in mice.BDetection of netrin‐1 expression in the MMTV/neuT model by immunoblots (Tumor 1 to 3).CH&S immunohistochemistry in formalin‐fixed paraffin‐embedded mammary glands of MMTV/neuT mice. Scale bar represents 200 μm.DMaximum Intensity Projection of Tomographic scintigraphy and X‐ray CT of the whole body of a transgenic MMTV/neuT mouse to develop mammary tumors, acquired 24, 48, and 72 h after injection of NP137‐NODAGA‐^111^In.EBiodistribution properties of NP137‐NODAGA‐^111^In in MMTV/neuT mice at 96 h (3 mice) and measured for all organs. Radioactivity incorporation was quantified by the percentage of the injected dose *per* gram of organ. Error bars indicate s.d.FNP137‐^177^Lu enhances MMTV/neuT mouse survival. Mice were treated at 3.5 months with PBS, DOTA‐NP137, or NP137‐^177^Lu. Kaplan–Meier survival curves of mice treated or not with NP137 and considered as dead when cumulative tumors volume reached 2,000 mm^3^. Mantel–Cox test; *n* = 10 animals/group for PBS; *n* = 14 animals/group for DOTA‐NP137; *n* = 8 animals/group for NP137‐^177^Lu; *P* < 0.001 between PBS and NP137‐^177^Lu and between DOTA‐NP137 and NP137‐^177^Lu. Source data are available online for this figure.

Xenografts and patient‐derived xenografts (PDXs) are highly important models for studying human disease, but they are limited in their capacity to reflect the pathophysiological conditions linked to tumor development and its microenvironment, including the vascularization and the immune microenvironment. In order to assess the value of radiolabeled NP137 in detecting and treating tumors in an *in vivo* setting reflecting human tumor development, we tested our radiotracers in the transgenic model of spontaneously developing luminal mammary cancer MMTV‐NeuT (Guy *et al*, 1992), which expressed netrin‐1 as illustrated by immunoblotting (Fig 5A and B). SPECT/CT imaging using NP137‐^111^In of *in situ* lesions showed a strong staining in fat pad tissues in all 10 mammary glands with incorporation peaking 48 h post injection (Fig 5C and D). A large tumor uptake close to 10% of ID/tumor at 96 h postinjection was measured in all tumors highlighting the sensitivity of the nuclear medicine contrast agent for netrin‐1‐positive tumors. The MMTV‐NeuT model is known to develop a number of mammary tumor foci in all mammary fat pads and it is very interesting to note that some tumors were visible even before their detection by mammary palpation, advocating for the use of NP137‐^111^In as a potential tool for early cancer detection and to monitor the appearance of metastatic lesions derived from netrin‐1‐overexpressing primary tumors (Fig 5D and E). As this model nicely mimics human disease, we therefore performed similar experiments for targeted radionuclide therapy with NP137‐^177^Lu in this transgenic mouse model that resulted in an increase in the overall survival, highlighting the strong antitumoral effect of the NP137‐^177^Lu radioimmunoconjugate (*P* < 0.001; Fig 5F). Taken together, the use of radiolabeled NP137 for theranostic purpose proved feasible. Netrin‐1‐positive tumors could be reliably identified and then subjected to targeted radiotherapy slowing down tumor growth and prolonging survival.

## Discussion

Radiolabeling is a very promising tool to trace, visualize, and detect a protein that is secreted, albeit trapped in the ECM as shown in the present proof‐of‐concept study for the axon guidance cue netrin‐1. However, other tumor‐specific matrix‐embedded proteins could probably be detected by similar strategies as long as these specific proteins—for example, axon guidance ligands, morphogens, and cellular secreted ligands—are specifically re‐expressed in cancer settings.

Here, we demonstrated that netrin‐1 was not freely diffusible in the cell microenvironment but adheres to the ECM. Indeed, netrin‐1 was initially described for its role in neural development as a secreted molecule that generates a diffusible gradient in the surrounding microenvironment (Serafini *et al*, 1996; Kennedy, 2006). This dogma was recently questioned, as netrin‐1 was shown to be actively transported during brain formation (Dominici *et al*, 2017; Varadarajan *et al*, 2017), and is corroborated by our results demonstrating that netrin‐1 is retained in the ECM by interacting with matrix proteins. We not only clearly observed its radiolabeled accumulation in the tumor (and probably ECM) but were also unable to detect netrin‐1 in the serum of patients and mice (by ELISA or mass spectrometry). Further studies are required to understand the transport/dissemination of netrin‐1 in order to provide a complete and more dynamic picture of the mechanisms in which it is involved, such as inflammation (Barnault *et al*, 2022).

Based on this lack of diffusibility, we elaborated a new companion test for the *in vivo* detection of netrin‐1. Netrin‐1 has been characterized as a therapeutic target in several types of cancers currently evaluated in several clinical phase Ia/b and II trials (NCT04652076; NCT05605496; NCT05546853; NCT05546879; Cassier *et al*, 2019). However, because of the lack of a conventional test (*i.e*., serum detection with ELISA, mass spectrometry, and robustness of pathological analyses quantifying netrin‐1 expression in FFPE samples), there is a need for an innovative, simple, and robust companion test to detect netrin‐1 expression in tumors. Having identified netrin‐1 as being immobilized and accessible in the ECM of tumor cells, we elaborated a new companion test for noninvasive *in vivo* imaging by a nuclear medicine SPECT/CT approach. Previously, an alternative imaging approach for netrin‐1 was explored using netrin‐1‐targeted microbubbles for ultrasound molecular imaging (Wischhusen *et al*, 2018). However, ultrasound molecular imaging is still in its infancy, and targeted microbubble contrast agents are not readily available for clinical translation, while 3D reconstructed SPECT/CT images are already the standard practice for patient care and can be used to visually identify uptake and perform image‐based quantification, as in current ^177^Lu‐based therapies (peptide receptor or PSMA; Strosberg *et al*, 2017; Sartor *et al*, 2021). Therefore, we selected SPECT/CT imaging as a novel imaging modality, which also provided us with the perspective of moving forward to targeted radiotherapy. To identify the best molecule for SPECT imaging, we designed three different agents based on the clinically used NP137 antibody: a complete NP137‐IgG1, NP137‐F(ab′)_2_, and NP137‐Fab. Even though all three molecules bound strongly to netrin‐1, a significant difference in their *in vivo* accumulation within tumors was observed; notably, a higher penetration of NP137‐IgG1 and NP137‐F(ab')_2_. This difference was likely due to the half‐life and stability of the molecules when injected into the bloodstream. The complete NP137‐IgG1 form displayed the best accumulation within tumors and the most promising results for transfer to the clinic as a companion test for the ongoing clinical trial to perform therapy response prediction to NP137 and monitor netrin‐1 expression during therapy. This tumor uptake is globally linked also to netrin‐1 protein levels, the antibody is not retained by 67NR tumors that do not express the target, whereas it is concentrated in the 3 tumor lines tested, that express netrin‐1 at different levels. Other parameters will have to be analyzed in future studies; tumor uptake is higher in 4T1 than in EMT6, which expresses more netrin‐1 *in vitro*. We can assume that the composition of the extracellular matrix plays a role in the retention and diffusion of netrin‐1. Other parameters also influence the binding of radiolabeled antibodies, such as the tumor microenvironment, and perfusion by blood and lymphatic vessels. It has also been described that the genetic background of animal models, in particular in immunodeficient animals, severely modifies tumor uptake, probably because the lack of circulating antibodies in these animals causes an injected antibody to be taken up by Fc receptors, and therefore strongly lowers its half‐life, diffusion and thus tumor incorporation (Sharma *et al*, 2018). This hypothesis may explain why we see less uptake of NP137‐^111^In in the human H358 model. Based on our results, we can state that there is a minimal off‐target signal with NP137‐^111^In in our preclinical models, as evidenced by the low tumor incorporation in the netrin‐1‐negative tumor model 67NR and in other organs. These results are in line with the fact that netrin‐1 is not widely expressed in adult noncerebral tissues and a knockdown of netrin‐1 in adult mice does not show any drastic phenotype. Instead, netrin‐1 seems specifically re‐expressed in tumoral tissues. The axonal guidance cue netrin‐1 is expressed in the adult central nervous system. However, selecting the full‐length antibody may have prevented penetration through the blood–brain barrier as there was no radioactivity detected in the brains of the animal models employed, suggesting a prevention of brain toxicity. More generally, targeting of proteins expressed by developmental genes that are re‐expressed during tumor formation seems to be key to improving the specificity and reliability of tumor imaging.

Based on the strong tumor incorporation of anti‐netrin‐1 mAb in netrin‐1‐positive tumors, we designed a new molecule, in which we fused NP137‐DOTA with lutetium‐177 to form NP137‐^177^Lu. Lutetium‐177 is a *ß* ^−^ emitter able to deliver a strong dose of radiation in a range of 1.8 mm within the tumor tissue (Strosberg *et al*, 2017; Sartor *et al*, 2021). Upon the specific accumulation of NP137‐^177^Lu in the netrin‐1‐positive tumor models, we noted a significant decrease in tumor growth, which was correlated with better survival of tumor‐bearing mice and confirmed that our strategy based on localized delivery of radiation to netrin‐1‐positive tumors in living animals was effective for tumor treatment. Of interest, in some of the models used here, for example, the EMT6‐engrafted model, NP137 as a single agent failed to show antitumor activity while a single NP137‐^177^Lu injection was associated with tumor growth‐inhibiting effects. The phase I and now ongoing phase II clinical trials of NP137 showed an excellent safety profile of NP137 and basically no toxicity related to target inhibition. Studies have shown that ^177^Lu‐based radiopharmaceuticals also have generally low side effects compatible with clinical development. Therefore, the combination of NP137 and ^177^Lu to generate the radiotherapeutic compound NP137‐^177^Lu provides an optimal treatment approach with high potential for clinical translation for use in patients with refractory tumors. Although our study showed no clear difference between DOTA and NODAGA, the choice of a single chelator will be important for faster clinical development to perform the imaging and therapy phases with the same NP137‐DOTA/NODAGA GMP batch.

Our study therefore provides important proof‐of‐concept data on the multifaceted use of NP137 not only for diagnostic but also therapeutic purpose and the immediate possibility to transfer ^111^In‐ and ^177^Lu‐radiolabeled NP137 to humans.

## Materials and Methods

### Human tumor samples

The usage of all patient tumor samples was conducted according to the rules of WMA Declaration of Helsinki. The Belmont Report and the Ethic Committee of the Medical Board (CHU Lyon) agreed to these investigations. Informed consent was obtained from all patients. The collection of samples has been performed on primary tumors from patients treated at the Hospices civils de Lyon and specimens are conserved in the pathology department.

### Tumor cell lines

4T1 and 67NR murine mammary carcinoma cells and human pulmonary adenocarcinoma H358 cells were obtained from ATCC and cultured in RPMI‐1640 (ATCC) medium supplemented with 10% fetal bovine serum (FBS, Gibco) and antibiotics (streptomycin and penicillin). EMT6 murine mammary carcinoma cells were obtained from ATCC and cultured in Eagle Minimum Essential Medium (EMEM, ATCC) supplemented with 10% FBS and antibiotics (streptomycin and penicillin). The cells were maintained in culture at 37°C under a humidified atmosphere composed of 20% O_2_ and 5% CO_2_ as previously described (Gibert *et al*, 2014).

The cells have been tested for mycoplasma infections for use in mice but were not authenticated prior to the submission of the article.

### Western blots

Confluent cells were washed with cold PBS and lysed in lysis buffer (Tris 10 mM pH 7.6; SDS 5%; glycerol 10%; Triton X‐100 1%, DTT 100 mM). After sonication, proteins were assayed using the Pierce 660 nm protein assay reagent (Thermo Fisher Scientific), run on 4–15% SDS–polyacrylamide gels (Bio‐Rad), and transferred to nitrocellulose membranes using the Trans‐Blot Turbo Transfer (Bio‐Rad). The membranes were blocked for 1 h at room temperature with 5% fat‐free milk powder for primary and with 5% bovine serum albumin (BSA). Staining was performed overnight with a primary antibody: netrin‐1 antibody (1/1000 dilution; Ab126729, Abcam); actin antibody (1/2500 dilution Sigma, A3854). After washing, the membranes were incubated with a secondary rabbit anti‐goat antibody coupled with horse radish peroxidase (HRP) for 1 h at room temperature. The West Dura (Pierce) chemiluminescence system was used to intensify the signal. Imaging was performed using Chemidoc Touch (Bio‐Rad).

For the binding of netrin‐1 in cellular matrix, 1 × 10^6^ cells were plated in a 100 mm^3^ culture dish. 24 h after, cells were treated with 200 μg/ml of heparin sodium salt from porcine intestinal mucosa (H3147‐100KU, Sigma) diluted in 4 ml of medium without FBS. After one night of incubation, the supernatant was collected. Centricons centrifugal filters were used to concentrate the protein in the collected supernatant. Pierce 660 nm protein assay reagent (22660, Thermo Fisher Scientific) was then used to determine the concentration of protein, 30 μg of proteins was loaded on immunoblots.

### *In vivo* preclinical models

The human monoclonal antibody NP137 (anti‐netrin‐1) was kindly provided by Netris Pharma (Lyon, France). Female Balbc/J mice, 8 weeks old, were obtained from Janvier Laboratories (Le Genest‐Saint‐Isle, France). All syngeneic breast cancer cells, 1 × 10^6^ of EMT6, 5 × 10^5^ of 4T1, and 1 × 10^6^ of 67‐NR, were subcutaneously transplanted into the dorsal flank of 8‐week‐old female Balbc/J mice. The mice were maintained under specific pathogen‐free conditions (Anican, Lyon, France, and Imthernat facility, HCL Lyon, France) and stored in sterilized cages with filter lids. Their care and accommodation were in accordance with European and French institutional guidelines as defined by the local CECCAP Ethics Committee (CECCAPP_CLB_2020_024; Jiang *et al*, 2021). The human cell lines H358 (1 × 10^6^ cells) were grafted into 8‐week‐old female NMRI immunocompromised mice and maintained under the same conditions.

Tumor volumes were assessed by measuring two perpendicular tumor diameters with a caliper three times a week. Individual tumor volumes were calculated as follows: *V* = (a*b ^2^)/2; a being the largest diameter, b the smallest. When tumors reached a volume of 200–400 mm^3^, mice were randomly separated into groups of animals and treated with either ^111^In‐NODAGA‐NP137, ^111^In‐NODAGA‐NP137‐Fab, ^111^In‐NODAGA‐NP137‐F(ab′)_2_, or ^111^In‐DOTA‐NP137 (see Materials and Methods below for their production) and subjected to live imaging. For all experiments, mice were anesthetized using a gas protocol (isoflurane/oxygen [2.5%/2.5%]).

Anticancer treatments with either PBS, NP137‐DOTA, or NP137‐DOTA‐^177^Lu (called hereafter NP137‐^177^Lu) were conducted in one single injection (when tumors reached 50–100 mm^3^) with 7–10 MBq/mouse of lutetium‐177. Tumor growth was monitored until tumor volume reached 2,000 mm^3^, which was the endpoint of the protocol for sacrifice and *ex vivo* analysis. The animals were at this stage considered dead in all the Kaplan–Meier survival analyses.

For immunohistochemistry (IHC) analysis, tumors were collected 10 days after treatment, embedded in paraffin, and sectioned into 10 μm slices. Tumor histology was studied after hematoxylin‐phloxinB‐saffron, Ki67, and cleaved caspase‐3 staining of tumor slides at the Pathology‐Research Platform (CRCL‐France).

MMTV‐NeuT mice were generated in the facilities of Anican (Lyon), France. Females spontaneously developed numerous tumor foci in the mammary glands (Guy *et al*, 1992). Imaging was performed under the same conditions as above. For the therapy part, mice were treated with either PBS, control antibody, or NP137‐^177^Lu 3 months after birth. Survival was determined when the cut‐off point of 2,000 mm^3^ of cumulative tumor volume was reached.

### Conjugation

1 ml of the anti‐netrin‐1 antibody, NP137 (10 mg/ml), was transferred to an Amicon Ultra‐15 50 k (UFC905096). Diafiltration against 0.1 M phosphate buffer (pH 8.0) treated with 1.2 g/l of Chelex 100 was then performed. This step was repeated seven times using 10 ml of 0.1 M phosphate buffer (pH 8.0) solution with a 25 min centrifugation at 4,900 rpm between each wash. NP137 concentration was then calculated with a nanodrop. The concentration of the antibody was adjusted in order to reach 50 μM. Stock solution of DOTA‐NHS ester or NODAGA (1,4,7‐triazacyclononane, 1‐glutaric acid‐44,7 acetic acid)‐NHS ester (CheMatech (C084)) was dissolved in ultrapure water at a concentration of 10 mg/ml (= 13.13 mM). 50 μM of NP137 was combined with required DOTA‐NHS or NODAGA‐NHS solution at a ratio of 1:25. Reactions were conducted at room temperature for 4 h and transferred to 4°C for continuous end‐over‐end mixing overnight. Diafiltration against PBS (Chelex) was then performed. This step was repeated seven times using 10 ml of PBS (Chelex) with a 25 min centrifugation at 4,900 rpm between each wash. DOTA/NODAGA‐NP137 concentration was calculated with a nanodrop.

### Generation of Fab and F(ab′)_2_ fragments and synthesis of DOTA and NODAGA‐immunoconjugates

Proteolytic fragments of NP137 were generated using Pierce™ Fab and F(ab′)_2_ Preparation kits according to the manufacturer's instructions (ref 44985 and 44988, Thermofisher, France). For conjugation of DOTA or NODAGA to surface lysine residues, NP137 and its fragments were conjugated at a molar ratio of 25:1 chelate:antibody with DOTA‐NHS‐ester or NODAGA‐NHS ester (Chematech, Dijon, France) in metal‐free buffers prepared using Chelex‐100 resin (Bio‐Rad, 142‐1253). Briefly, 50 μM antibodies were exchanged by diafiltration against 0.1 M phosphate buffer (pH 8.0), then mixed with 1.25 mM DOTA‐NHS‐ester or NODAGA‐NHS‐ester for 4 h at 25°C on a rotator. The reaction was placed at 4°C for continuous end‐over‐end mixing overnight. Excess chelator was removed by diafiltration against PBS. Immunoconjugates were stored at 4°C.

### Radiolabeling

NODAGA‐NP137, NODAGA‐NP137‐Fab, or NODAGA‐NP137‐F(ab′)_2_ (40–70 μl, 5 mg/ml) were radiolabeled by adding 400 μl of acetate buffer 0.1 M pH 5.5 and 40–400 MBq of high purity ^111^InCl_3_ in hydrochloric acid solution (Curium, Petten, The Netherlands). The mixture was incubated for 30 min at 37°C. The reaction was stopped with 100 μl of a 1 mM aqueous solution of DTPA. Free ^111^In was removed using a PD‐10 column (GE Healthcare, GE17‐0851‐01). The column was first washed with 15 ml of 0.1 M pH 5.5 acetate buffer, the labeled mixture was then loaded onto the column and eluted with acetate buffer. ^111^In‐NODAGA‐NP137, ^111^In‐NODAGA‐NP137‐Fab or ^111^In‐NODAGA‐NP137‐F(ab′)_2_ were first eluted. The radiochemical purity (RCP) of each 0.5 ml fraction was evaluated using ITLC‐SG (Biodex, Tec‐control black) and 50 mM citrate buffer (pH 5.5) as the mobile phase and was assayed with a gamma isotope TLC analyzer (Scan‐Ram, Lablogic, Villebon‐sur‐Yvette, France). Radiolabeled NP137 remained at the origin while unbound ^111^In^3+^ or ^111^In‐DTPA complex migrated with an Rf of 0.9–1. The fractions with the highest radiochemical purity (RCP) were pooled.

For stability testing, an aliquot of radiolabeled ^111^In ‐NODAGA‐NP137, ^111^In‐NODAGA‐NP137‐Fab or ^111^In‐NODAGA‐NP137‐F(ab′)_2_ was incubated at 37°C in 2 ml PBS (pH 7.4) and the RCP was evaluated using ITLC‐SG and 0.1 M citrate buffer pH 5.5 as the mobile phase.

^177^Lu‐radiolabeled DOTA was obtained according to the procedure described above starting from ^177^LuCl_3_ in hydrochloric acid (Endolucin®, ITM, Germany).

### Biodistribution studies

1–10 MBq of radiolabeled ^111^In‐NODAGA‐NP137, ^111^In‐NODAGA‐NP137‐Fab, ^111^In‐NODAGA‐NP137‐F(ab′)_2_ or ^111^In DOTA‐NP137, in a maximum volume of 100 μl were injected intravenously (IV) into tumor‐bearing mice (*n* = 3 or 4 for each group). Mice were then sacrificed at defined times: 4, 24, 48, 72, and 96 h after injection by cervical dislocation. Tissues of interest (blood, heart, lungs, gonads, bones, spleen, kidneys, muscle, digestive tract, brain, and skin) were removed, weighed and the radioactivity was counted for 5 min in a gamma scintillation counter (Wizard® gamma counter, Perkin Elmer, USA). Urine and feces were collected in an individual metabolic cage for housing and counting. Tissue distribution was expressed as a percentage of the injected dose *per* gram (%ID/g). Renal and hepatobiliary eliminations were expressed as cumulative radioactivity under the total activity injected.

### Imaging

Image acquisition was performed using a Nano‐SPECT/CT system for small animals (Bioscan, Washington, DC, USA). This system consists of four detectors (215 × 230 mm^2^ NaI, 33 PMTs) equipped with interchangeable multi‐pinhole collimators. SPECT/CT acquisitions were performed after IV injection of 10–15 MBq of radiolabeled molecules at different times: 24, 48, 72, and 96 h. CT (55 kVp tube voltage, 500 ms exposure time, and 180 projections) and SPECT/CT acquisitions were done in tumor‐bearing anesthetized mice in a pron position, placed on a temperature‐controlled bed (Minerve, Esternay, France), in order to maintain body temperature (set at 37°C). Image acquisition was performed for 40 min with two 15% windows centered on the two peaks 171 and 245 keV of ^111^In. All images were reconstructed and analyzed using the InVivoscope software (Bioscan, Washington, DC, USA).

### BioLayer interferometry

The affinity of the antibody fragments for netrin‐1 was determined by biolayer interferometry using the OctetRed96 system (FortéBio) at 30°C with constant shaking at 1,000 rpm in PBS, 0.02% Tween‐20, 0.1% BSA (binding buffer or BB). Briefly, recombinant human netrin‐1 (R&D)‐coated HIS1K biosensors were incubated with different concentrations of antibody or fragments, and the association was observed for 5 min. Biosensors were then incubated further in BB for 5 min to observe dissociation of the complex. Binding kinetics were evaluated with ForteBio Octet RED Evaluation software 6.1 using a 1:1 binding model to derive K_on_, K_off_, and K_d_ values.

For netrin‐1 binding within matrix proteins, all interactions were analyzed at 30°C with constant shaking at 1,000 rpm in BB. Netrin‐1‐coated‐HIS1K biosensors were incubated with an increasing concentration series of m‐Laminin‐I (3.12, 6.25, 12.5, 25, 50, 100 nM) and binding was observed for 5 min at 30°C. Netrin‐1‐coated‐AHC biosensors were incubated with an increasing concentration series of hIntegrin, hGlypican‐1, hFibronectin, hVitronectin, hLumican, and hFLRT‐1 (3.12, 6.25, 12.5, 25, 50, 100 nM) and binding was observed for 5 min at 30°C. Biosensors were then incubated further in BB for 5 min to observe dissociation of the complex.

### Autoradiography

NP137‐^111^In uptake in tumors was analyzed by autoradiography in Balbc/j mice bearing 4T1 and EMT6 xenografts. 72 h after intravenous injection of NP137‐^111^In (7.67 ± 3.03 MBq), mice (EMT6 mice *n* = 3, 4T1 mice *n* = 5) were sacrificed using CO_2_ chambers. Whole bodies were immediately frozen in liquid nitrogen. Sections (40 μm) were cut at 100‐μm intervals; sagittal slices were exposed after drying to a Biospace β Imager (Biospace Mesures, Paris, France) for 6 h. Autoradiography imaging was performed at “*In Vivo* Imaging in Auvergne” (IVIA) facility (https://doi.org/10.18145/ivia).

### Stability assays

For stability testing, an aliquot of the purified NP137‐^177^Lu was incubated at 37°C in 2 ml PBS (pH 7.4). RCP was evaluated using ITLC‐SG (biodex, Tec‐control black) and citrate buffer 100 mM (pH 5.5) as the mobile phase. NP137‐^177^Lu remained at the origin whereas unbound ^177^Lu^3+^ migrated with an Rf of 0.9–1.

### Statistical analyses

Randomization was done for the animals to homogenize the groups around 100 mm^3^ of tumor volume for the NP137‐^177^Lu treatments. There were no exclusion criteria, except absence of tumors. The experiments were not blinded.

All statistical analyses were performed using the GraphPad Prism software (San Diego, California). Statistical (non/parametric) tests: Mann–Whitney (*U*‐test), ANOVA, or unpaired Student *T*‐test were specified within the text. Survival curves were produced according to the Kaplan–Meier method. Data were analyzed with a Mantel–Cox test. *n* defines the total number of replicates. All statistical tests were two‐sided and are specified in the text of the manuscript or in figure legends.

**The paper explained**

**Problem**

In oncology, despite their name, targeted therapies are often given without patient prescreening. Immunohistochemistry, the gold standard for tumor diagnosis, is largely conducted on the primary tumor, and this diagnosis may vary or evolve once metastases appear at a much later stage, including when no primary tumor is present anymore. Near‐real‐time diagnostic approaches are therefore urgently needed.

**Results**

Using netrin‐1 and its blocking antibody as a proof‐of‐concept for tumor therapy, we were able to visualize this tumor cell‐specific protein by nuclear imaging. We used this finding to design a near‐real‐time companion test for netrin‐1 therapy. Tumor incorporation of anti‐netrin‐1 antibody being highly efficient, we created a second therapeutic molecule by fusing this antibody to Lutetium 177, a radioelement capable of destroying cancer cells upon exposure. Mechanistically, we demonstrated that netrin‐1, although secreted, is not as diffusible as previously described but remains trapped in the extracellular matrix and microenvironment of cancer cells, justifying its potential as a target for vectorized radiotherapy.

**Impact**

Netrin‐1 and more generally matrix‐embedded proteins are promising targets for both molecular imaging and radioimmunotherapy.

## Author contributions

**Patrick Mehlen:** Supervision; writing – original draft; writing – review and editing. **David Kryza:** Formal analysis; supervision; funding acquisition; investigation; project administration. **Jennifer Wischhusen:** Conceptualization; data curation; formal analysis; investigation; writing – original draft; writing – review and editing. **Mathieu Richaud:** Data curation; formal analysis; investigation. **Maëva Hervieu:** Data curation; formal analysis; investigation. **Jacqueline Sidi Boumedine:** Resources; data curation; formal analysis; methodology. **Jean‐Guy Delcros:** Data curation; formal analysis. **Sophie Besse:** Formal analysis; investigation. **Thomas Baudier:** Data curation; funding acquisition. **Pierre‐Alexandre Laval:** Data curation; funding acquisition; investigation. **Silvia Breusa:** Data curation; investigation. **Erwan Boutault:** Data curation; investigation; methodology. **Hugo Clermidy:** Data curation; formal analysis. **Nicolas Rama:** Formal analysis; investigation. **Benjamin Ducarouge:** Data curation; investigation. **Mojgan Devouassoux‐Shisheboran:** Resources; investigation. **Jean‐Michel Chezal:** Conceptualization; supervision; investigation. **Anne‐Laure Giraudet:** Methodology. **Thomas Walter:** Methodology. **David Sarrut:** Conceptualization; data curation; formal analysis; investigation; methodology; project administration. **Benjamin Gibert:** Conceptualization; supervision; funding acquisition; writing – original draft; project administration; writing – review and editing.

## Disclosure and competing interests statement

BD and PM declare to have a conflict of interest as, respectively, employee (BD) and shareholder (PM) of Netris Pharma.

## Supporting information



Expanded View Figures PDF

Source Data for Expanded View

PDF+

Source Data for Figure 1

Source Data for Figure 2

Source Data for Figure 3

Source Data for Figure 4

Source Data for Figure 5

## Data Availability

This study includes no data deposited in external repositories.
