# Supplementary material for: From netrin‐1‐targeted SPECT/CT to internal radiotherapy for management of advanced solid tumors
Source: EMBO Mol Med. 2023 Mar 6;15(4):e16732. doi: 10.15252/emmm.202216732 (PMC10086585; doi:10.15252/emmm.202216732)
Supplement: Supplementary file 1 — Expanded View Figures PDF [file EMMM-15-e16732-s002.pdf]

Expanded View Figures

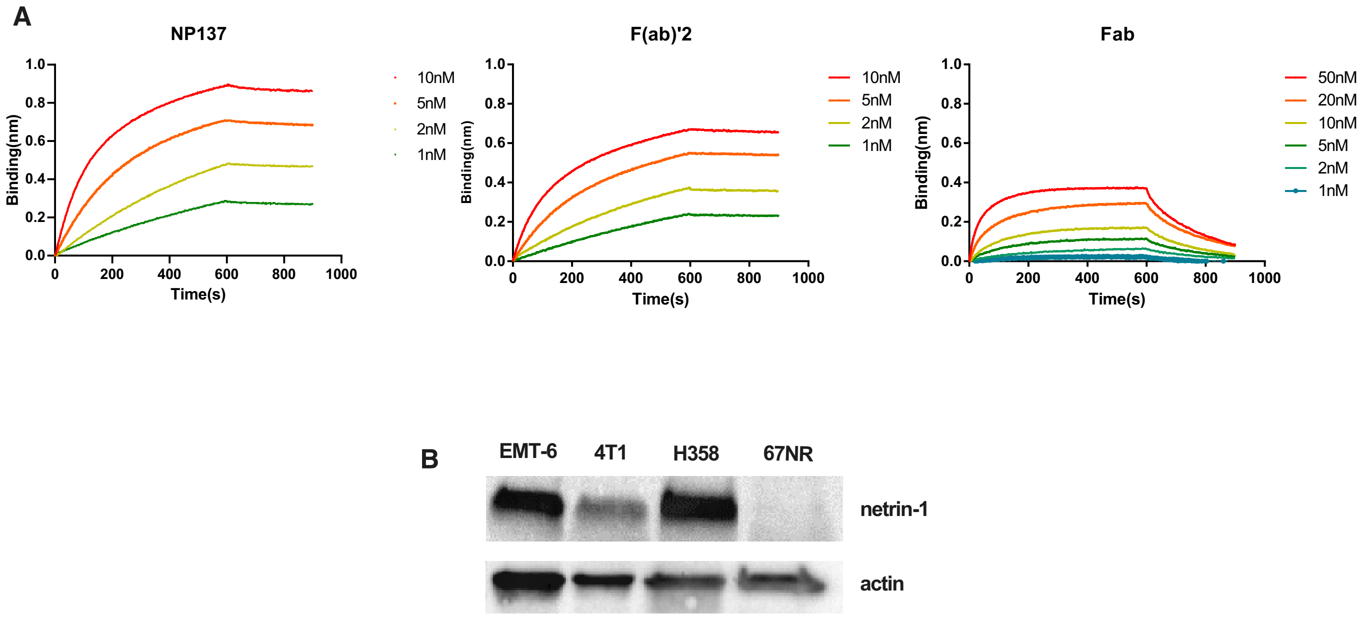

Figure EV1. NP137 fragments analysis and netrin-1 expression in tumor cell models.

A Biolayer interferometry kinetic analysis of NP137 and its F(ab′)$_2$ and Fab fragments on netrin-1. Colors indicate the concentration of NP137 and derivates.
B Quantification by immunoblots of netrin-1 expression in the cell lines used in the study.

Source data are available online for this figure.

 © 2023 The Authors

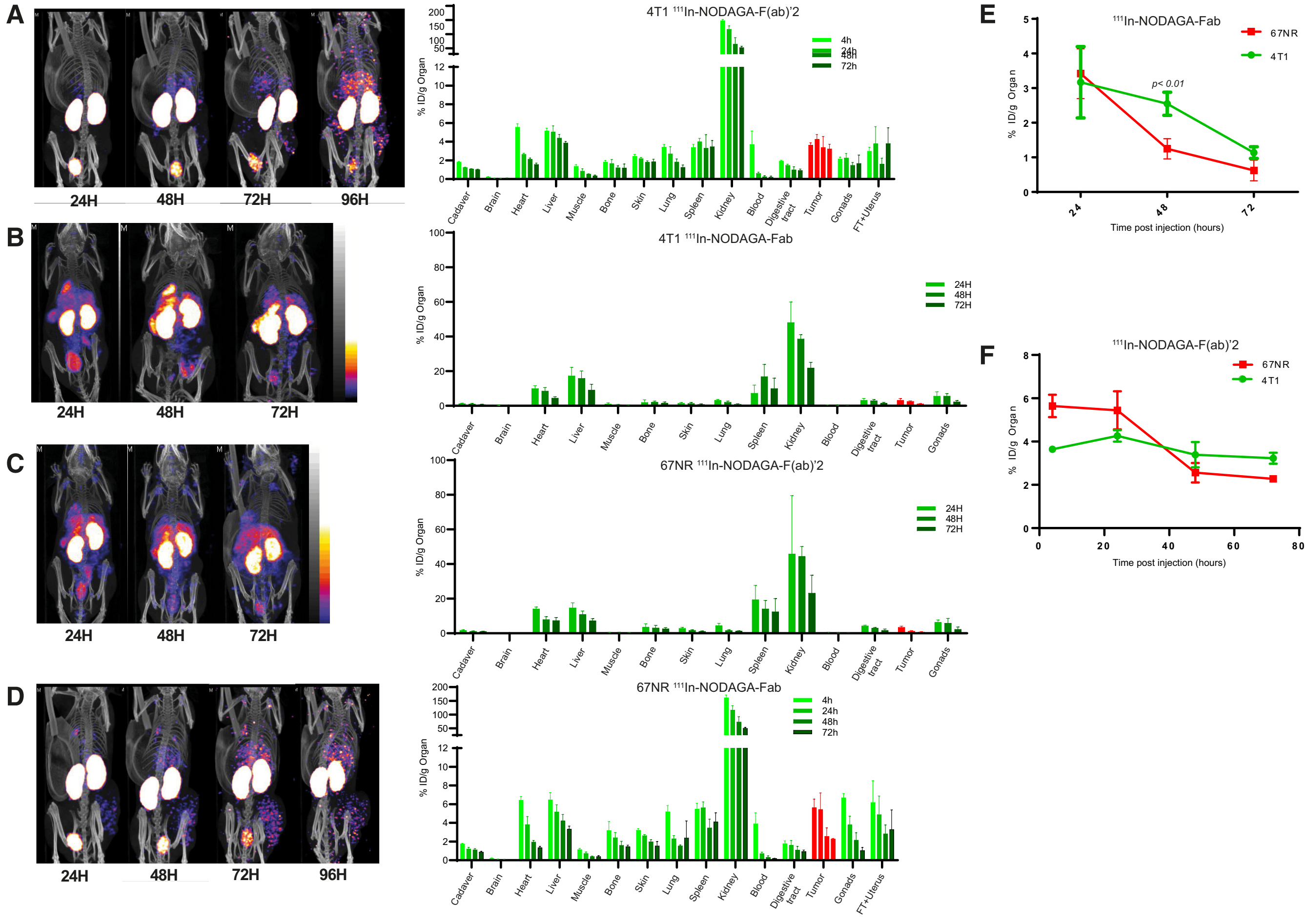

Figure EV2. ***In vivo*** **detection of netrin-1 using NP137-Fab or NP-137-F(ab')$_2$ constructs.**

A (Left) Maximum Intensity Projection of Tomographic scintigraphy and X-ray CT of the whole body of a Balb/c mouse bearing a 4T1 tumor (netrin-1-negative), acquired 24, 48h, 72, and 96 h after IV injection of Fab-NODAGA-^{111}In. (Right) Biodistribution properties of Fab-NODAGA-^{111}In in Balb/cJ mouse bearing 4T1 xenografts at 4 h (3 mice), 24 h (4 mice), 48 h (4 mice), and 72 h (4 mice) and measured for all organs. Radioactivity incorporation was quantified by the percentage of the injected dose per gram of organ. Error bars indicate s.d.

B (Left) Maximum Intensity Projection of Tomographic scintigraphy and X-ray CT of the whole body of a Balb/c mouse bearing a 4T1 (netrin-1-positive) tumor, acquired 4, 24, 48, and 72 h after IV injection of F(ab)'$_2$-NODAGA-^{111}In. (Right) Biodistribution properties of F(ab')$_2$ -NODAGA-^{111}In in a Balb/cJ mouse bearing 4T1 xenografts at 4 h (5 mice), 24 h (5 mice), 48 h (5 mice), and 72 h (5 mice) and measured in all organs. Radioactivity incorporation was quantified by the percentage of the injected dose *per* gram of organ. Error bars indicate s.d.

C (Left) Maximum Intensity Projection of Tomographic scintigraphy and X-ray CT of the whole body of a Balb/c mouse bearing a 67NR tumor (netrin-1-negative), acquired 24, 48, and 72 h after IV injection of F(ab')$_2$-NODAGA-^{111}In. (Right) Biodistribution properties of F(ab')$_2$-NODAGA-^{111}In in a Balb/cJ mouse bearing 67NR xenografts at 24 h (3 mice), 48 h (4 mice), and 72 h (4 mice) and measured for all organs. Radioactivity incorporation was quantified by the percentage of the injected dose *per* gram of organ. Error bars indicate s.d.

D (Left) Maximum Intensity Projection of Tomographic scintigraphy and X-ray CT of the whole body of a Balb/c mouse bearing a 67NR (netrin-1-negative) tumor, acquired 24, 48, and 72 h after IV injection of Fab-NODAGA-^{111}In. (Right) Biodistribution properties of Fab-NODAGA-^{111}In in a Balb/cJ mouse bearing 67NR xenografts at 4 h (3 mice), 24 h (4 mice), 48 h (4 mice), and 72 h (3 mice) and measured in all organs. Radioactivity incorporation was quantified by the percentage of the injected dose per gram of organ. Error bars indicate s.d.

E Tumor biodistribution ratio of Fab-NODAGA-^{111}In in Balb/cJ mice bearing 4T1 xenografts versus 67NR xenografts at 4 h (5 4T1 mice and 3 67NR mice), 24 h (5 4T1 mice and 4 67NR mice), 48 h (5 4T1 mice and 4 67NR mice), and 72 h (5 4T1 mice and 4 67NR mice); Two-way ANOVA. Error bars indicate s.d.

F Tumor biodistribution ratio of F(ab')$_2$-NODAGA-^{111}In in Balb/cJ mice bearing 4T1 xenografts versus 67NR xenografts at 4 h (3 mice), 24 h (4 mice), 48 h (4 mice), and 72 h (4 mice); Two-way ANOVA; Error bars indicate s.d.

Source data are available online for this figure.

© 2023 The Authors

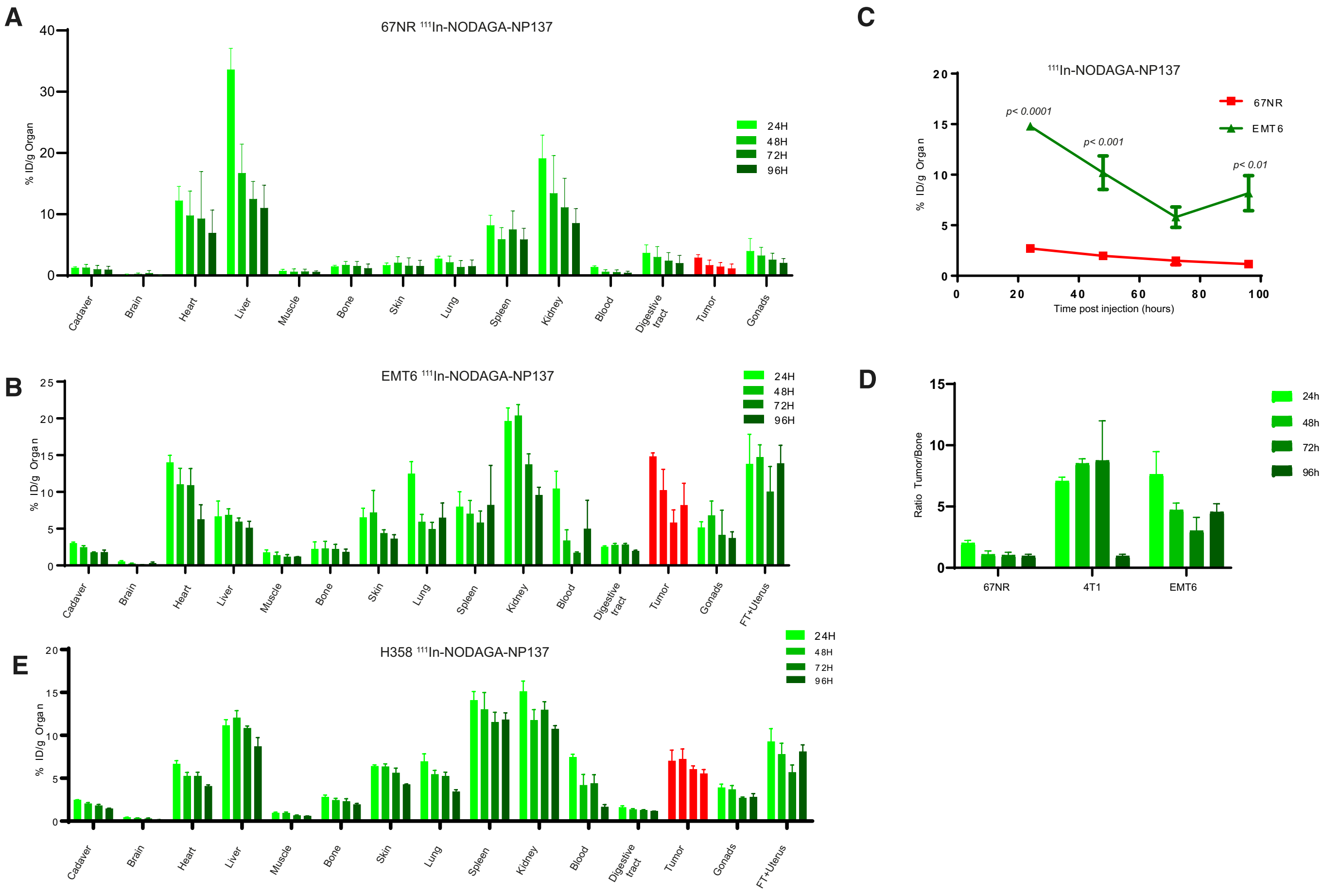

Figure EV3. Biodistribution analysis of radiolabeled NP137.

A Biodistribution properties of NP137-NODAGA-^{111}In in a Balb/cJ mouse bearing 67NR xenografts at 24 h (3 mice), 48 h (4 mice), 72 h (4 mice), and 96 h (4 mice) and measured for all organs. Radioactivity incorporation was quantified by the percentage of the injected dose per gram of organ. Error bars indicate s.d.

B Biodistribution properties of NP137-NODAGA-^{111}In in a Balb/cJ mouse bearing EMT6 xenografts at 24 h (3 mice), 48 h (3 mice), 72 h (3 mice), and 96 h (3 mice) and measured in all organs. Radioactivity incorporation was quantified by the percentage of the injected dose *per* gram of organ. Error bars indicate s.d.

C Tumor biodistribution ratio of NP137-NODAGA-^{111}In in Balb/cJ mice bearing EMT6 xenografts versus 67NR xenografts at 24 h (3 mice), 48 h (4 67NR mice and 3 EMT6 mice), 72 h (4 67NR mice and 3 EMT6 mice), and 96 h (4 67NR mice and 3 EMT6 mmice); Two-way ANOVA. Error bars indicate s.e.m.

D Ratio of tumor versus bone with the percentage of injected dose per gram of organ of NP137-NODAGA-^{111}In in Balb/cJ mice bearing 67NR, 4T1 or EMT6 xenografts at 24 h (5 67NR mice, 4 4T1 mice, and 3 EMT6 mice), 48 h (5 67NR mice, 4 4T1 mice, and 3 EMT6 mice), 72 h (5 67NR mice, 3 4T1 mice, and 3 EMT6 mice), and 96 h (4 67NR mice, 4 4T1 mice, and 3 EMT6 mice). Error bars indicate s.e.m.

E Tumor biodistribution profile of NP137-NODAGA-^{111}In in NMRI Nude mice bearing H358 tumors at 24 h (5 mice), 48 h (5 mice), 72 h (5 mice), and 96 h (4mice). Error bars indicate s.d.

Source data are available online for this figure.

© 2023 The Authors

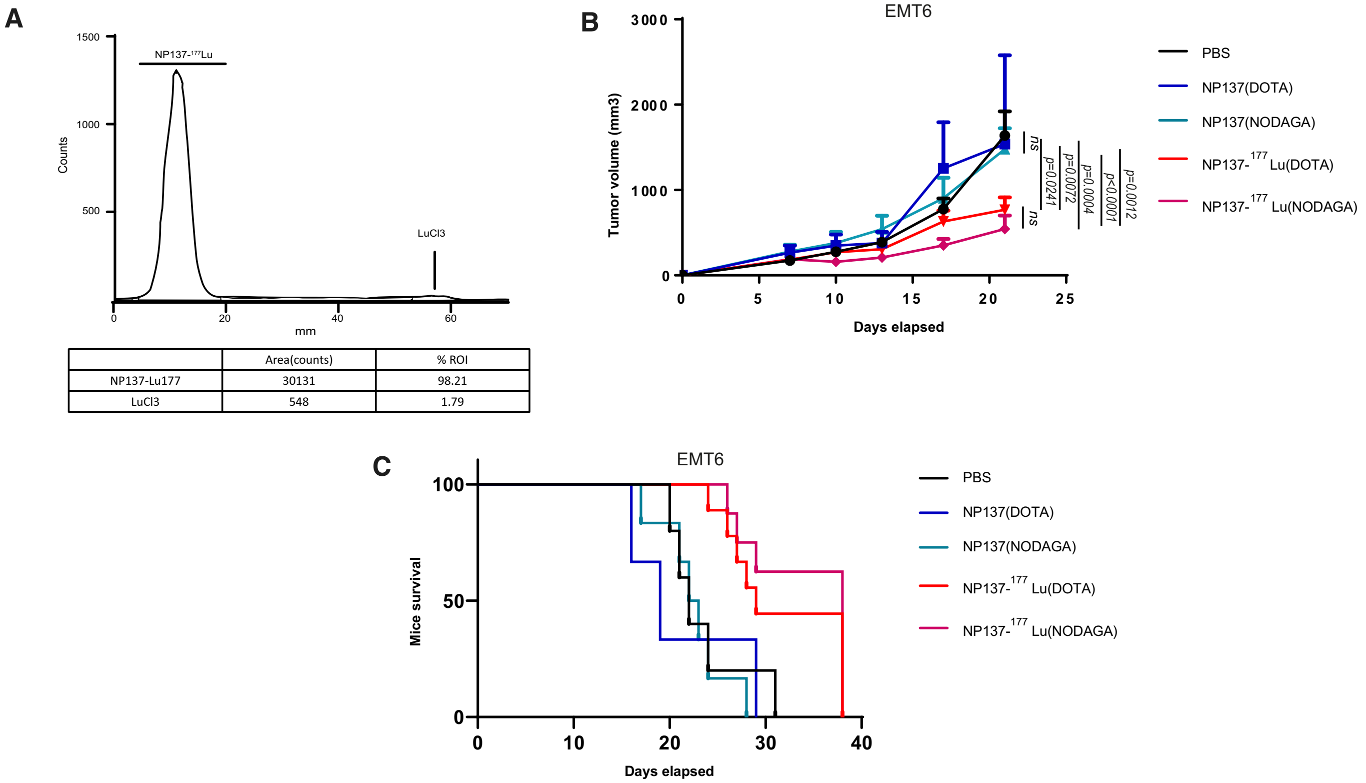

	Area(counts)	% ROI
NP137-Lu177	30131	98.21
LuCl3	548	1.79

Figure EV4. Analysis of chelation of ^{177}Lu with DOTA or NODAGA.

A Radiochemical purity evaluation of NP137-^{177}Lu incubated 7 days in human plasma samples.

B Balb/cJ mice were engrafted with EMT6 cells by subcutaneous injection of 1 million cells. After 5 days, animals were treated by IV injection of PBS; DOTA-NP137; NODAGA-NP137; DOTA-NP137-^{177}Lu; or NODAGA-NP137-^{177}Lu. $n = 5$ animals/group for PBS; $n = 3$ animals/group for DOTA-NP137; $n = 6$ animals/group for NODAGA-NP137; $n = 9$ animals/group for DOTA-NP137-^{177}Lu; $n = 7$ animals/group for NODAGA-NP137-^{177}Lu. $P = 0.0241$ between DOTA-NP137 and DOTA-NP137-^{177}Lu.; $P < 0.001$ between NODAGA-NP137 and NODAGA-NP137-^{177}Lu. Turkey's multiple comparison test. Error bars indicate s.e.m.

C No difference in survival between DOTA-NP137-^{177}Lu and NODAGA-NP137-^{177}Lu in mice engrafted with EMT6. Kaplan–Meier survival curves of mice treated or not with NP137. Mantel–Cox test; $n = 5$ animals/group for PBS; $n = 3$ animals/group for DOTA-NP137; $n = 6$ animals/group for NODAGA-NP137; $n = 9$ animals/group for DOTA-NP137-^{177}Lu; $n = 7$ animals/group for NODAGA-NP137-^{177}Lu.

Source data are available online for this figure.
