# Supplementary material for: From netrin‐1‐targeted SPECT/CT to internal radiotherapy for management of advanced solid tumors
Source: EMBO Mol Med. 2023 Mar 6;15(4):e16732. doi: 10.15252/emmm.202216732 (PMC10086585; doi:10.15252/emmm.202216732)
Supplement: Supplementary file 4 — Source Data for Figure 1 [file EMMM-15-e16732-s003.zip › Figure 1/1E/Quantification of netrin-1, Blot.pptx]

## Slide 1
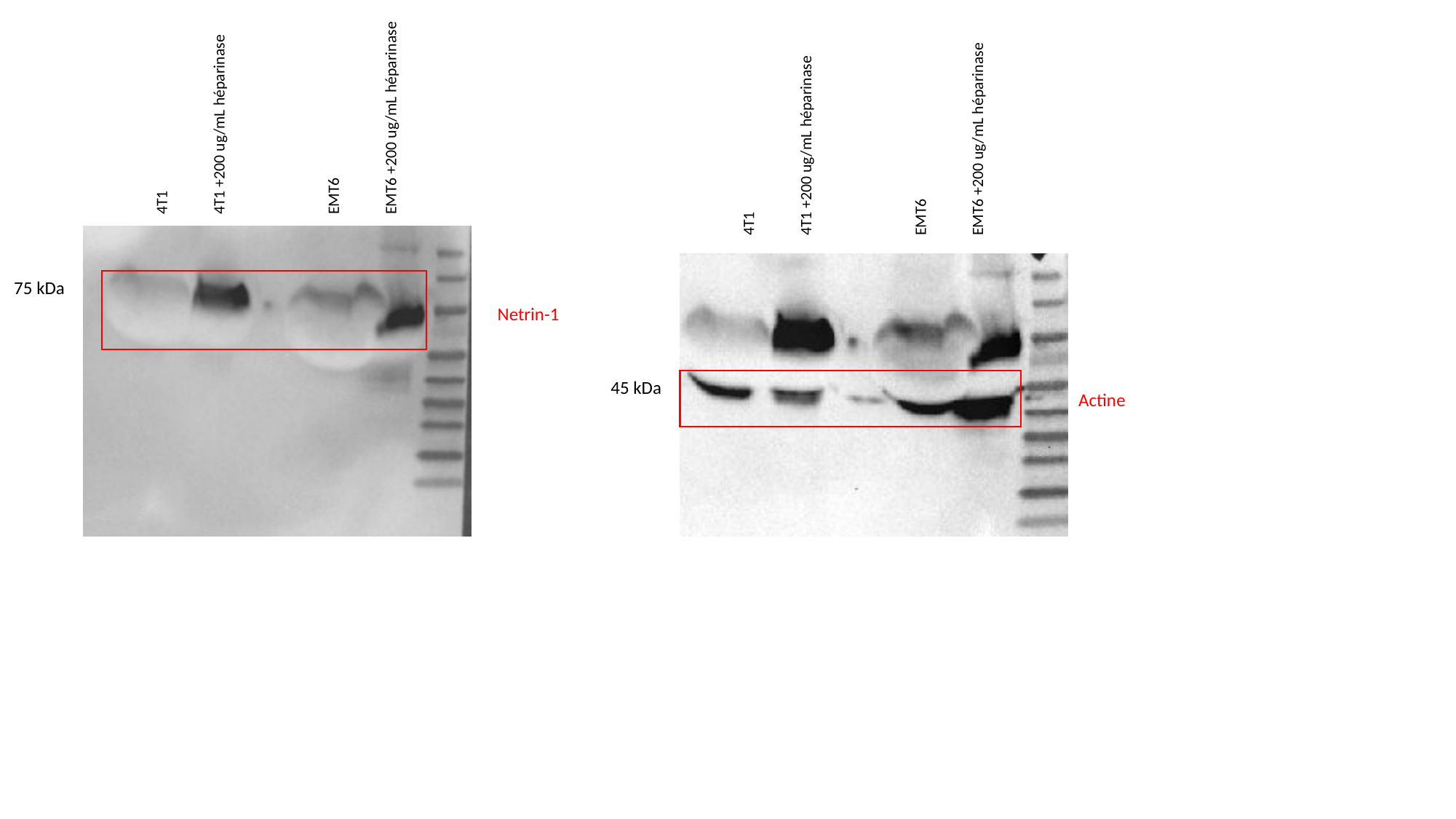

4T1
4T1 +200 ug/mL héparinase
EMT6
EMT6 +200 ug/mL héparinase
4T1
4T1 +200 ug/mL héparinase
EMT6
EMT6 +200 ug/mL héparinase
75 kDa
Netrin-1
45 kDa
Actine
