# Supplementary figures and images for: From netrin‐1‐targeted SPECT/CT to internal radiotherapy for management of advanced solid tumors
Source: EMBO Mol Med. 2023 Mar 6;15(4):e16732. doi: 10.15252/emmm.202216732 (PMC10086585; doi:10.15252/emmm.202216732)

## Slide 1
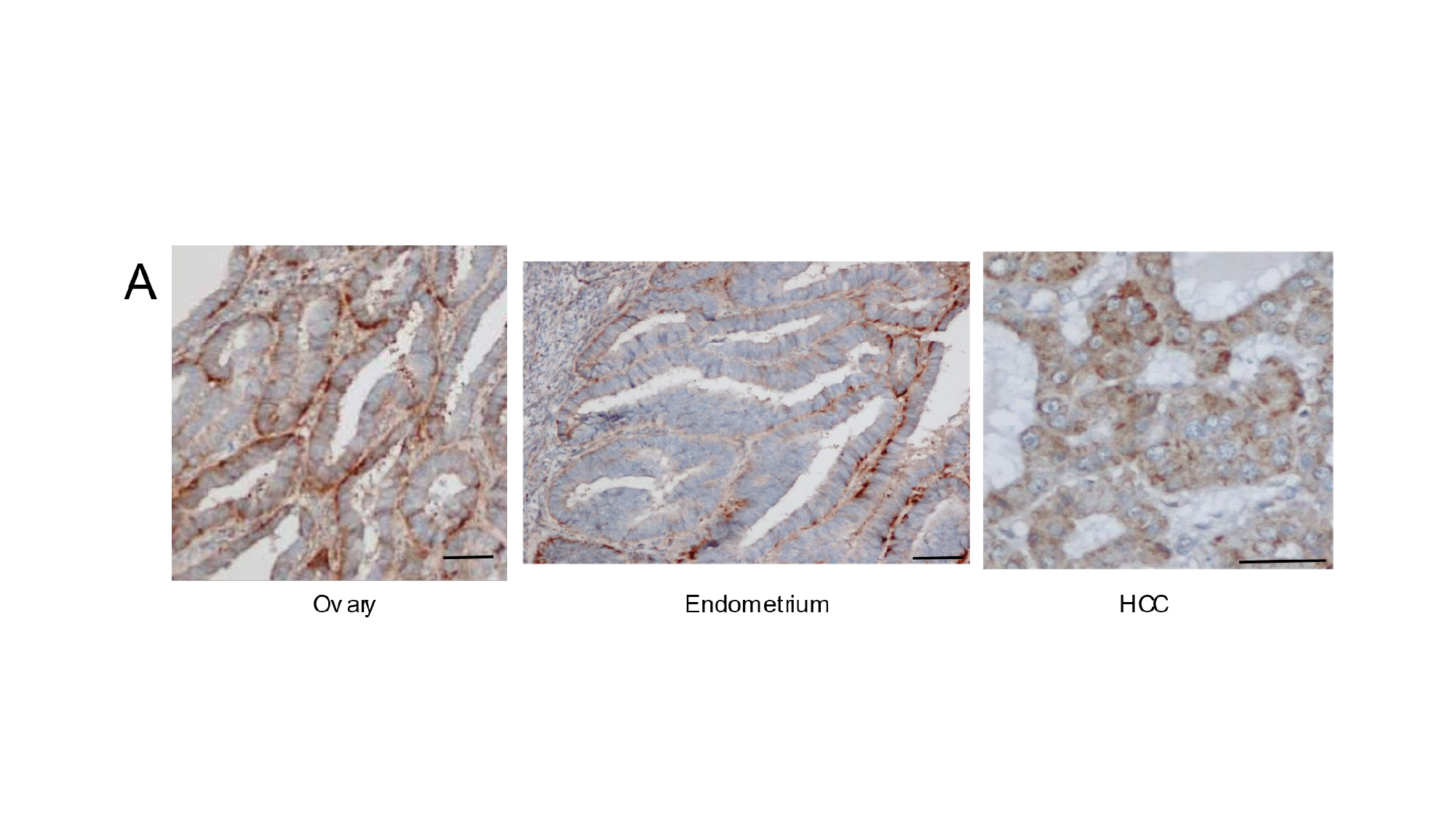

Supplement: Supplementary file 4 — Source Data for Figure 1 [file EMMM-15-e16732-s003.zip › Figure 1/1A/Netrin-1 , Micr.image IHC.pptx]

## Slide 1
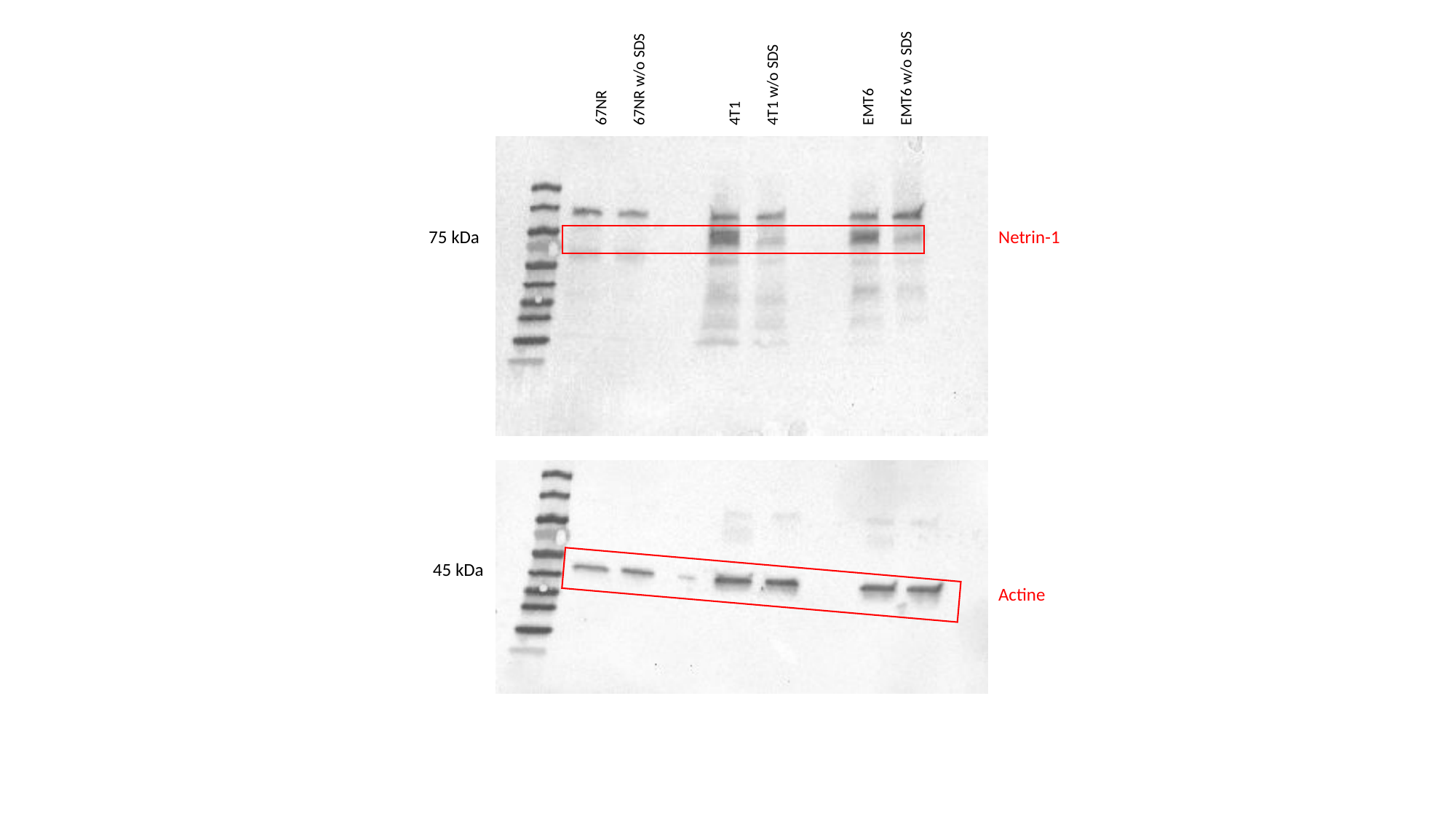

67NR
67NR w/o SDS
4T1
4T1 w/o SDS
EMT6
EMT6 w/o SDS
75 kDa
Netrin-1
45 kDa
Actine

Supplement: Supplementary file 4 — Source Data for Figure 1 [file EMMM-15-e16732-s003.zip › Figure 1/1D/Quantification of netrin-1, Blot.pptx]

## Slide 1
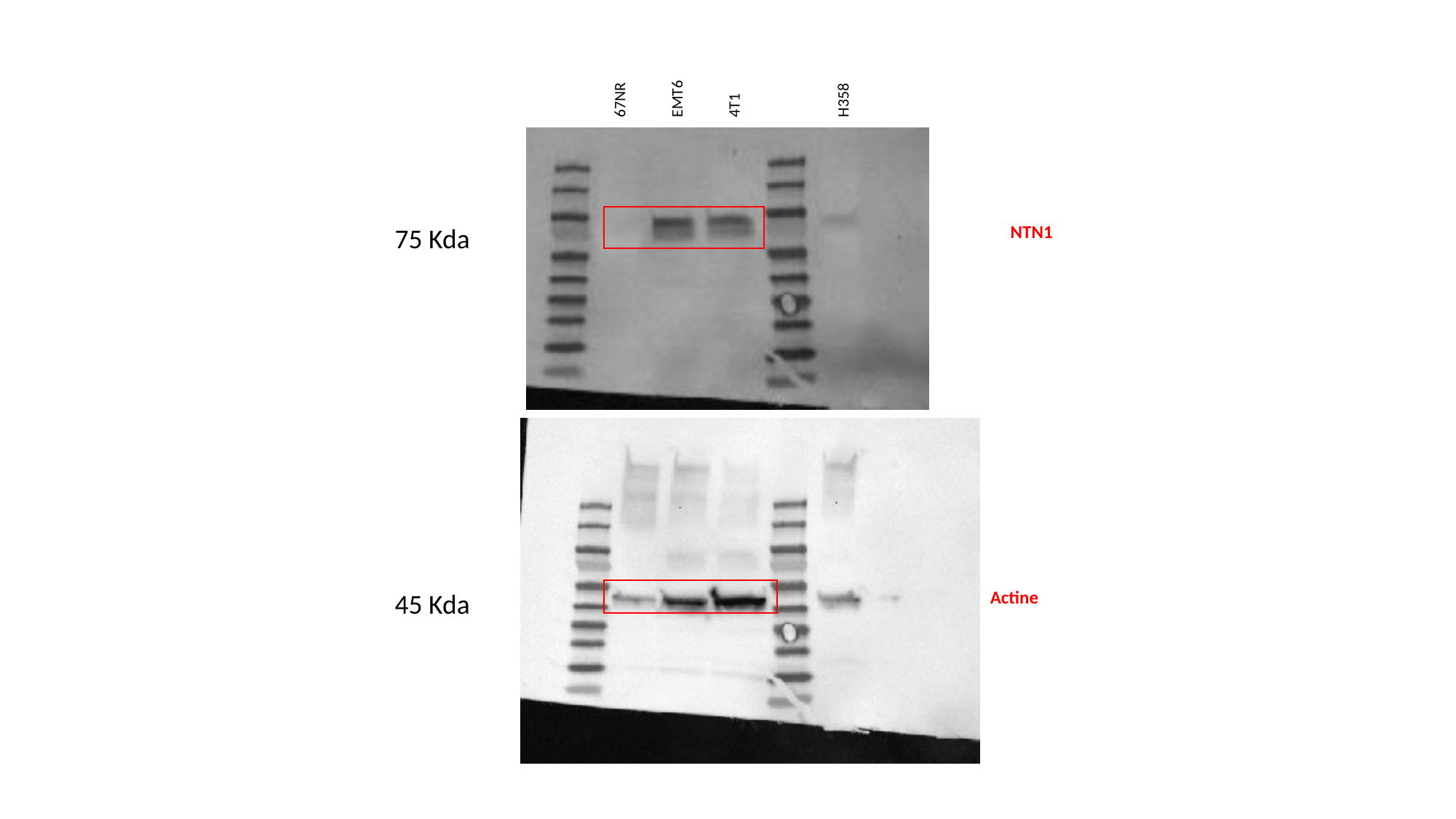

67NR
EMT6
4T1
H358
NTN1
75 Kda
Actine
45 Kda

Supplement: Supplementary file 4 — Source Data for Figure 1 [file EMMM-15-e16732-s003.zip › Figure 1/1C/Models expressing netrin-1 + actin, Blot.pptx]

## Slide 1
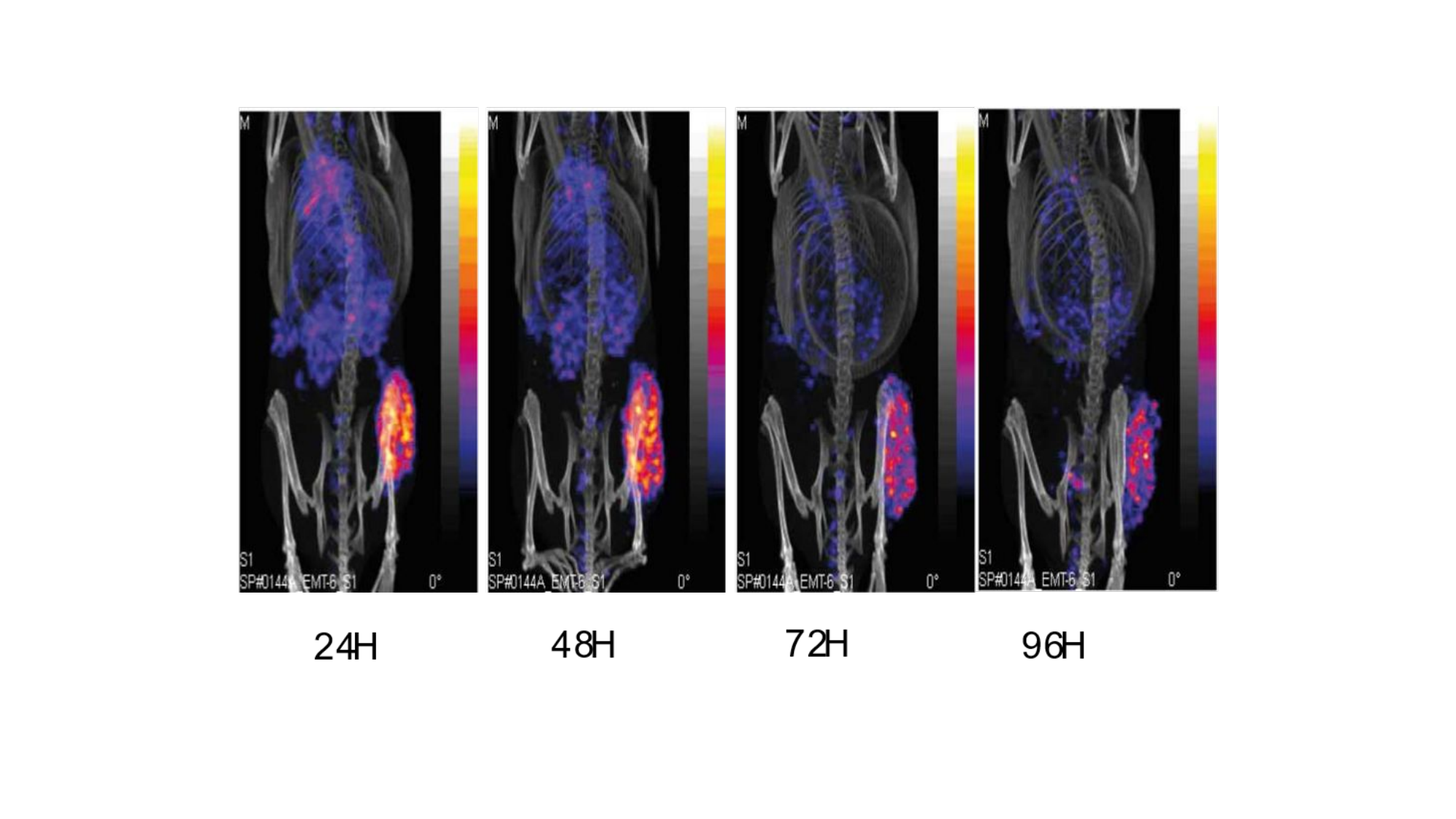

Supplement: Supplementary file 6 — Source Data for Figure 3 [file EMMM-15-e16732-s006.zip › Figure 3/3E/Tomographic scintigraphy and X-ray CT, Combi.pptx]

## Slide 1
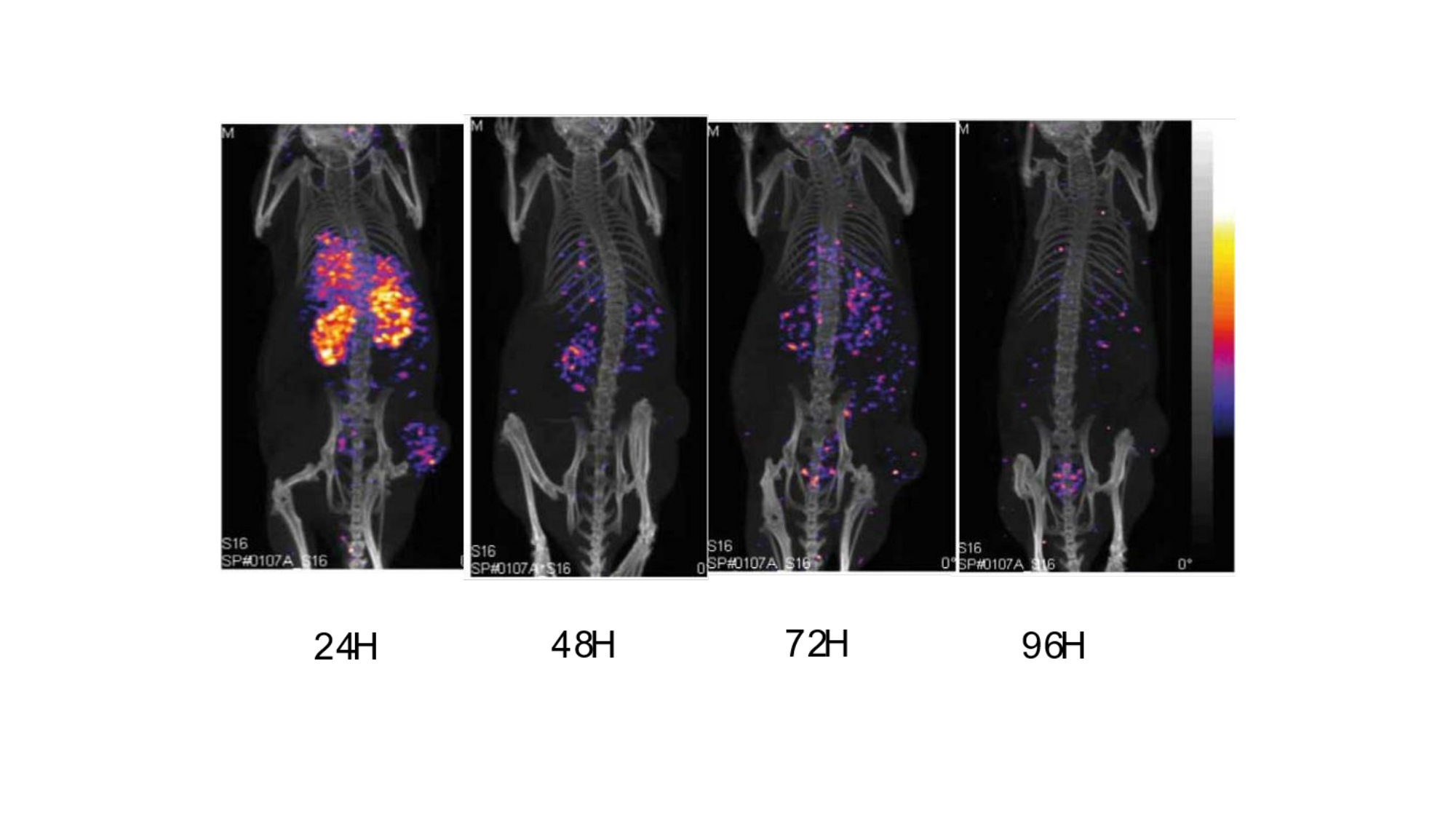

Supplement: Supplementary file 6 — Source Data for Figure 3 [file EMMM-15-e16732-s006.zip › Figure 3/3B/Tomographic scintigraphy and X-ray CT, Combi.pptx]

## Slide 1
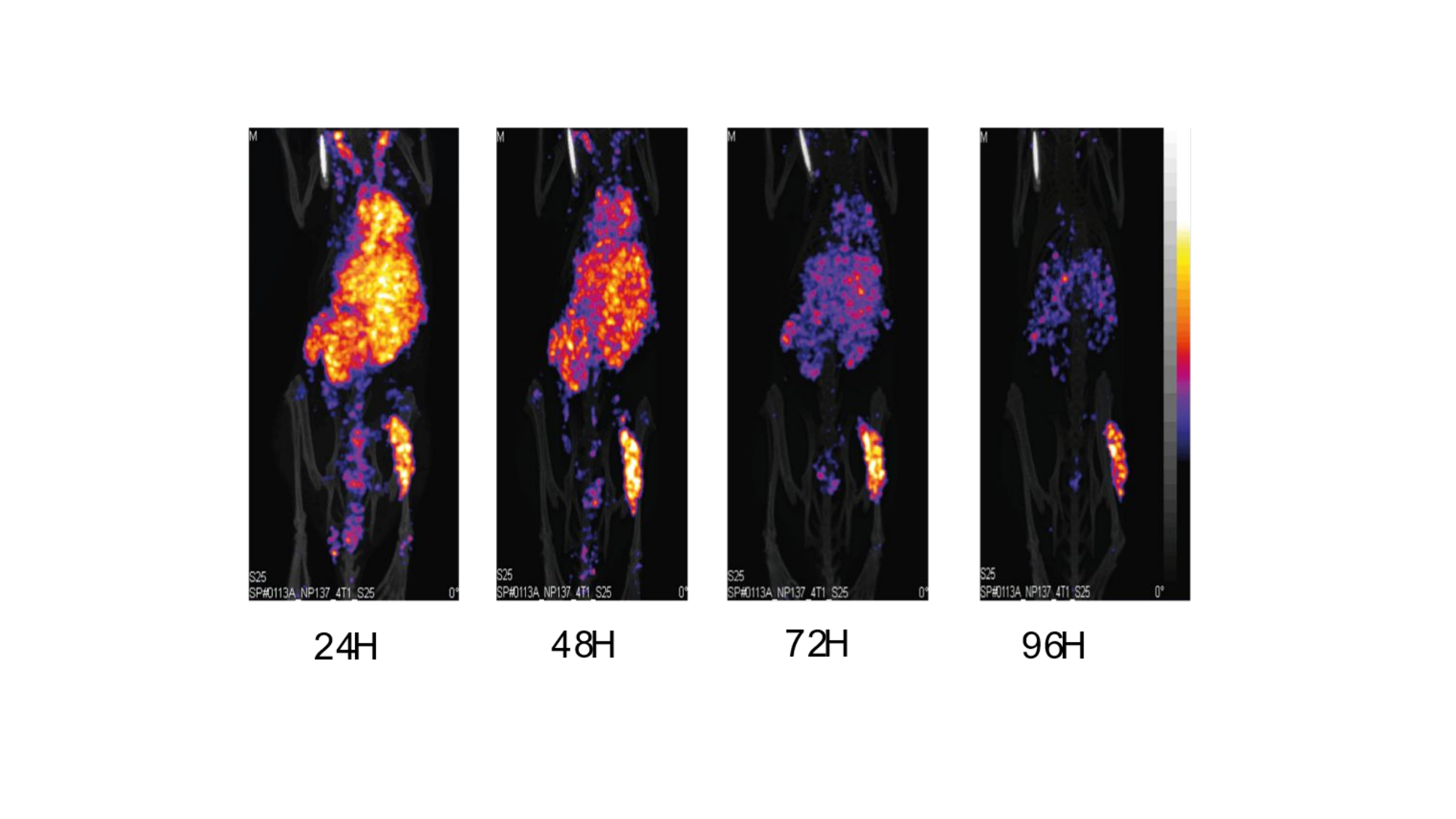

Supplement: Supplementary file 6 — Source Data for Figure 3 [file EMMM-15-e16732-s006.zip › Figure 3/3A/Tomographic scintigraphy and X-ray CT, Combi.pptx]

## Slide 1
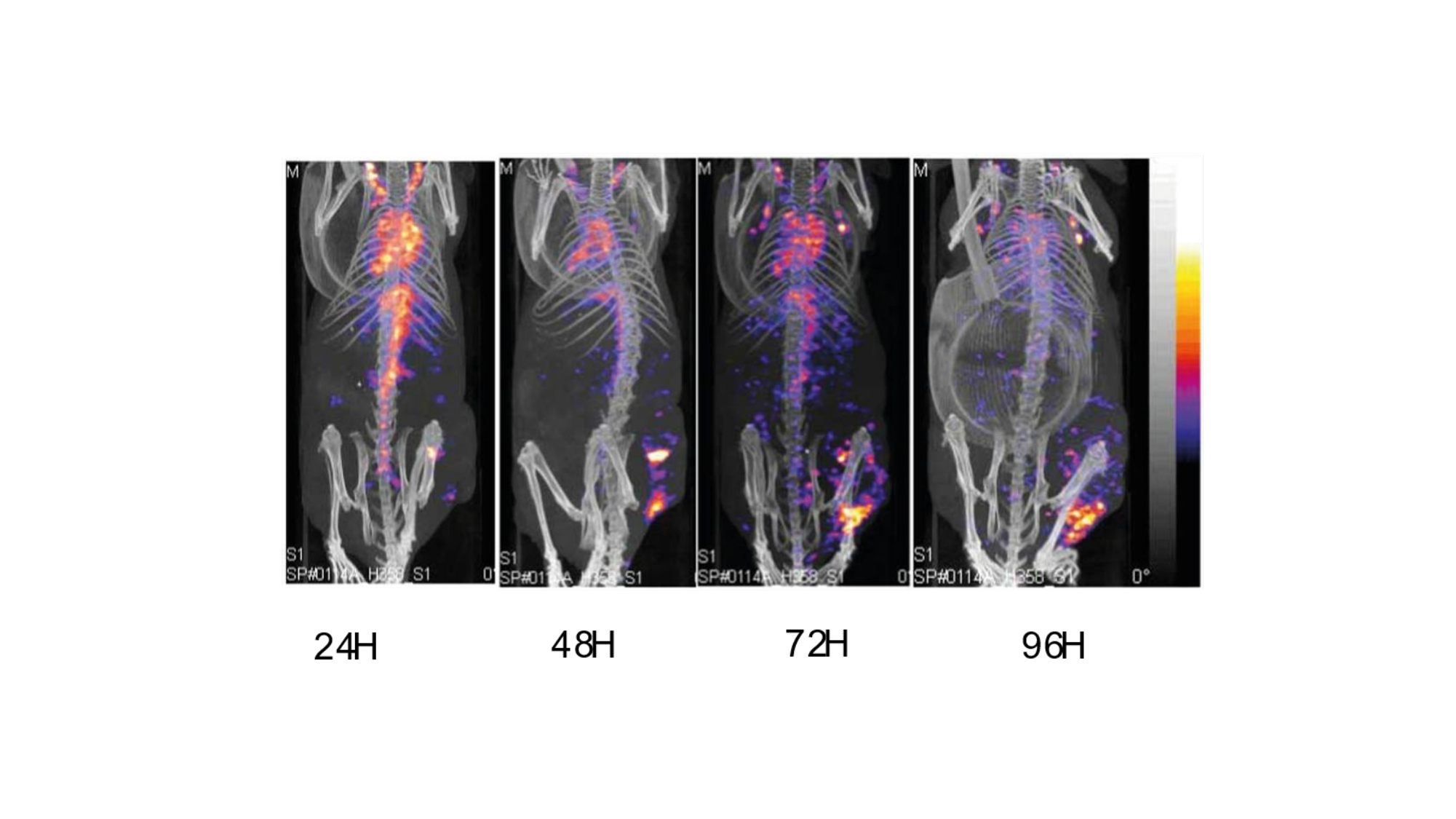

Supplement: Supplementary file 6 — Source Data for Figure 3 [file EMMM-15-e16732-s006.zip › Figure 3/3F/Tomographic scintigraphy and X-ray CT, Combi.pptx]

## Slide 1
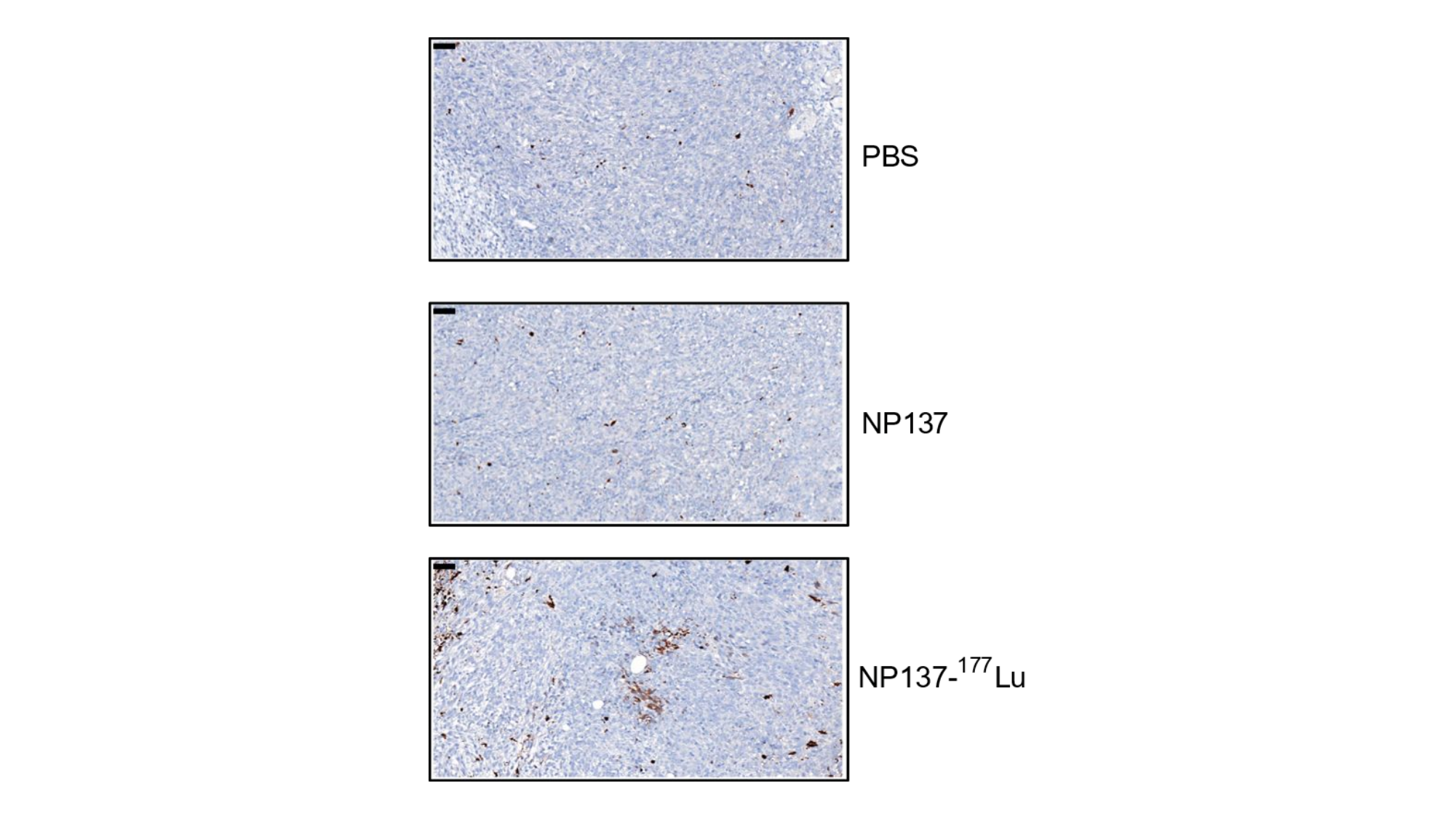

Supplement: Supplementary file 7 — Source Data for Figure 4 [file EMMM-15-e16732-s009.zip › Figure 4/4E/Cleaved caspase-3 expression, Micr.image.pptx]

## Slide 1
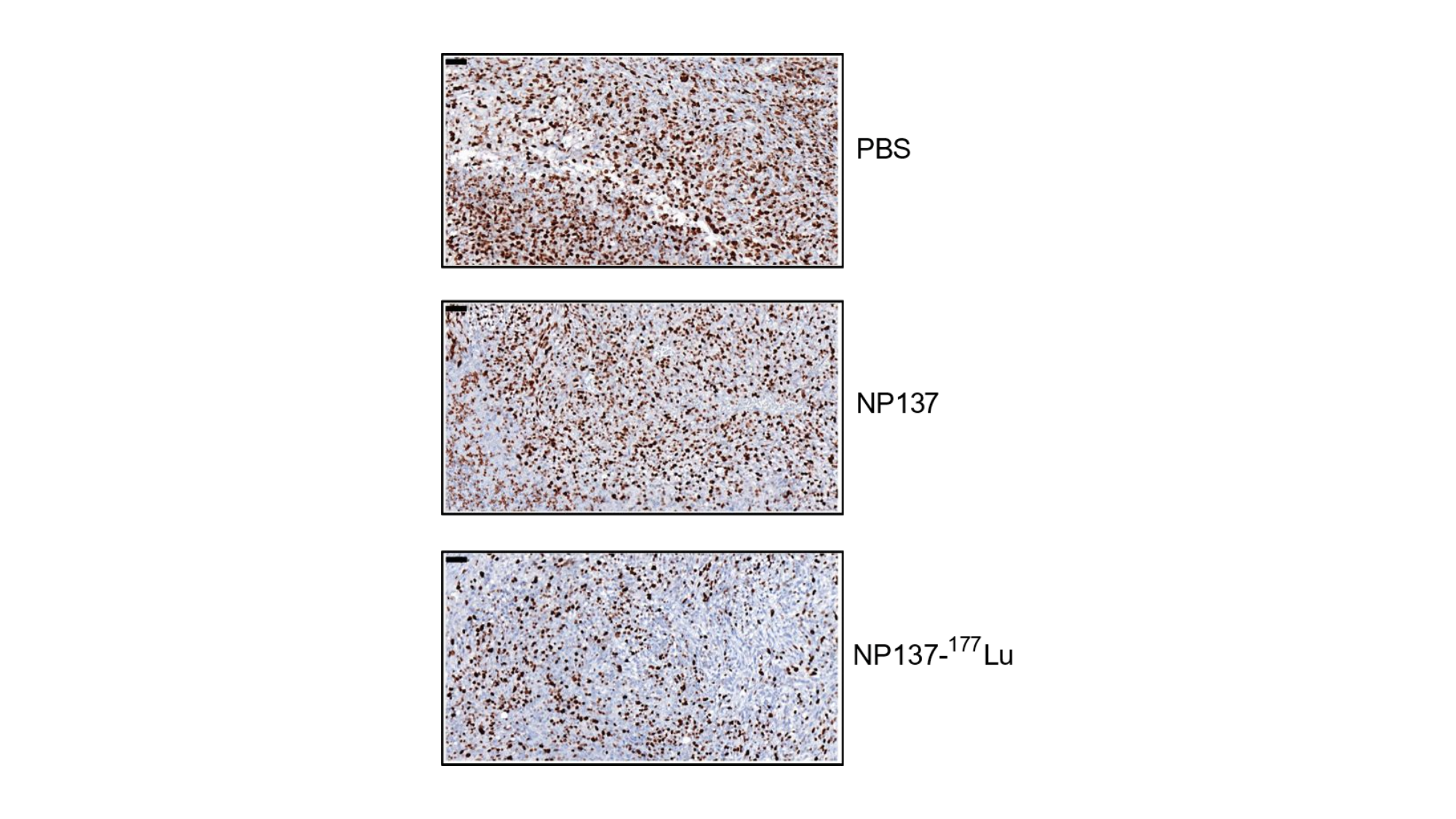

Supplement: Supplementary file 7 — Source Data for Figure 4 [file EMMM-15-e16732-s009.zip › Figure 4/4D/Ki-67 staining, Micr.image.pptx]

## Slide 1
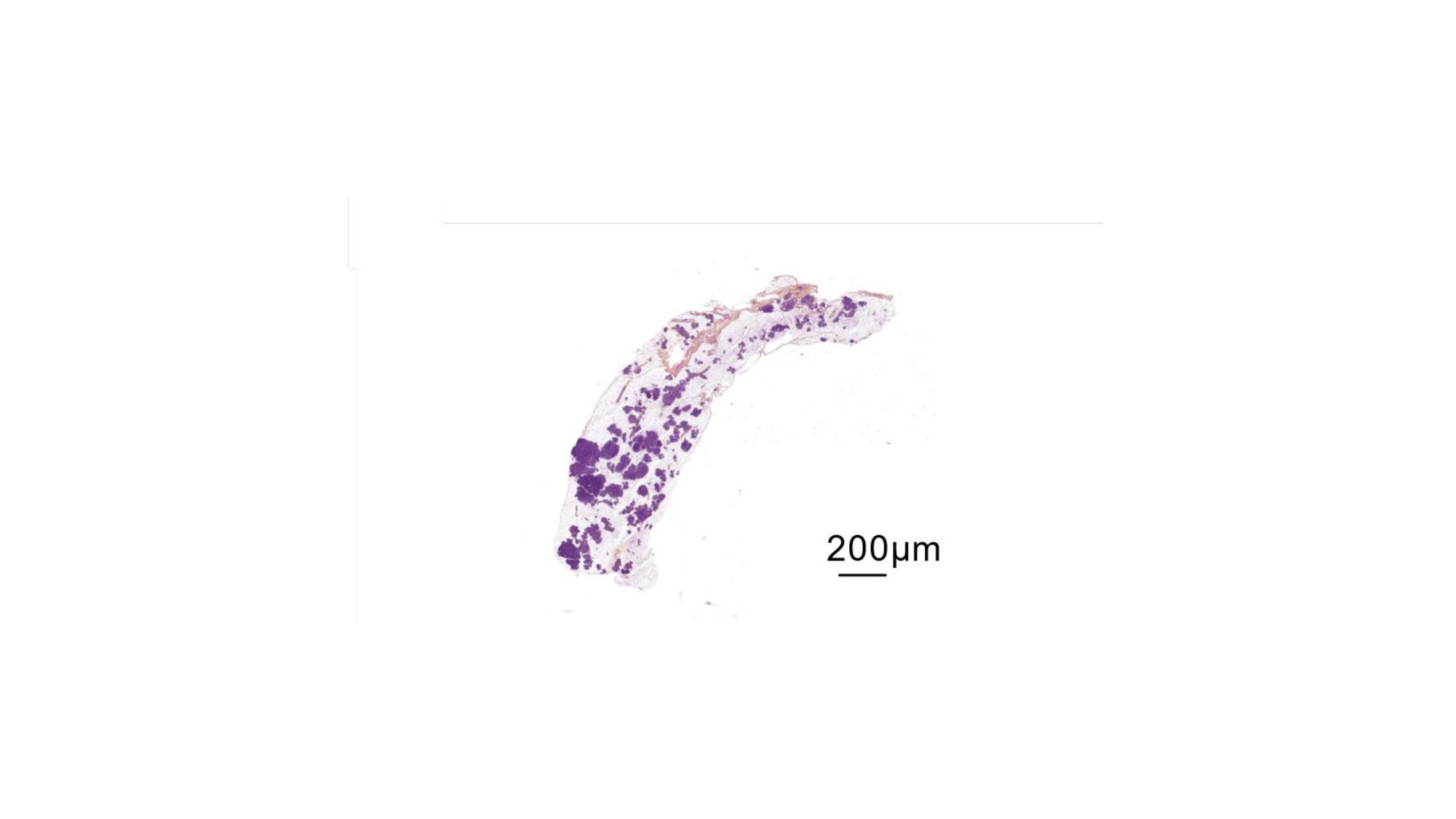

Supplement: Supplementary file 8 — Source Data for Figure 5 [file EMMM-15-e16732-s007.zip › Figure 5/5C/H&S immunohistochemistry, Micr.image IHC.pptx]

## Slide 1
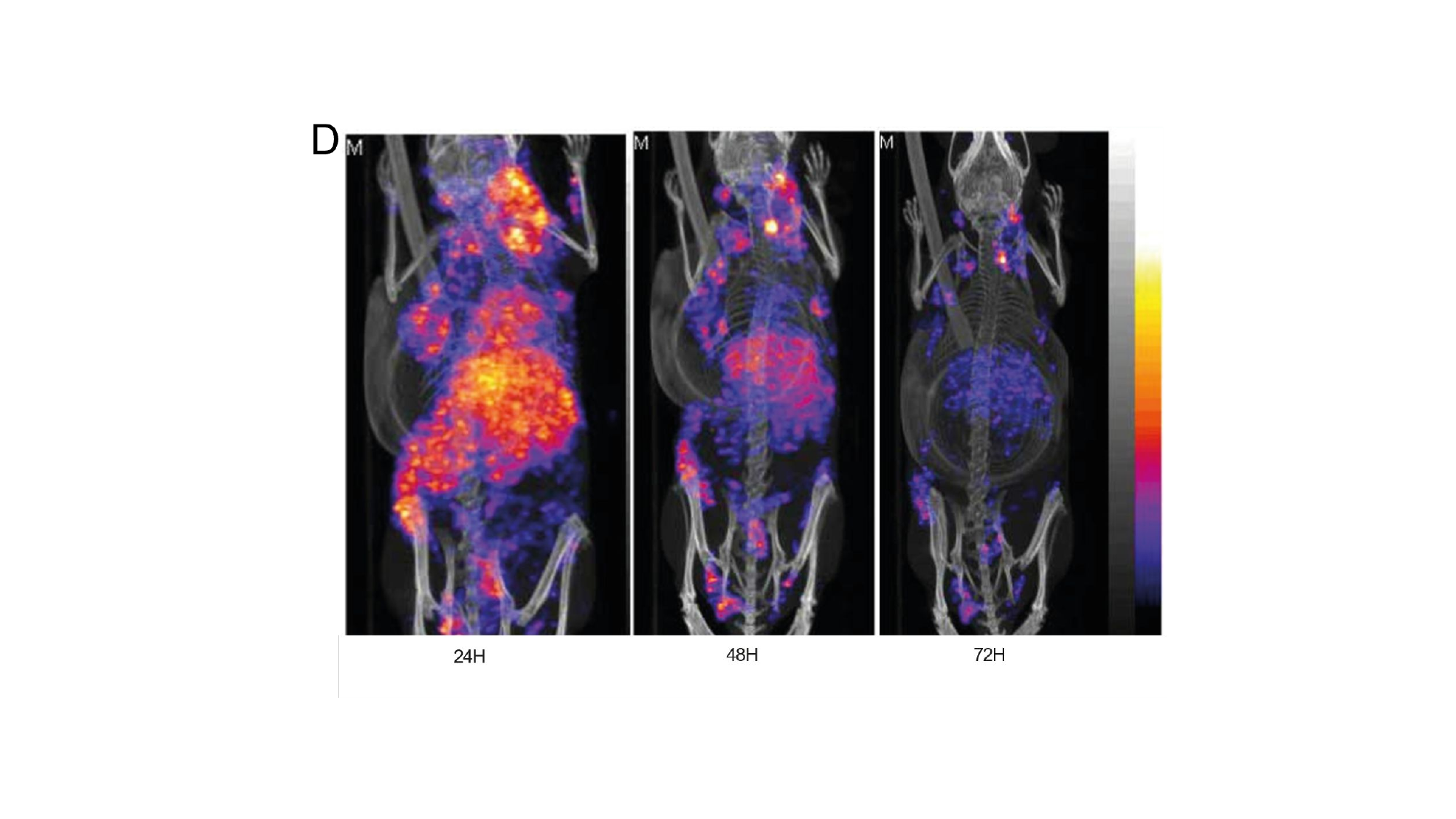

Supplement: Supplementary file 8 — Source Data for Figure 5 [file EMMM-15-e16732-s007.zip › Figure 5/5D/Tomographic scintigraphy and X-ray CT, Combi.pptx]

## Slide 1
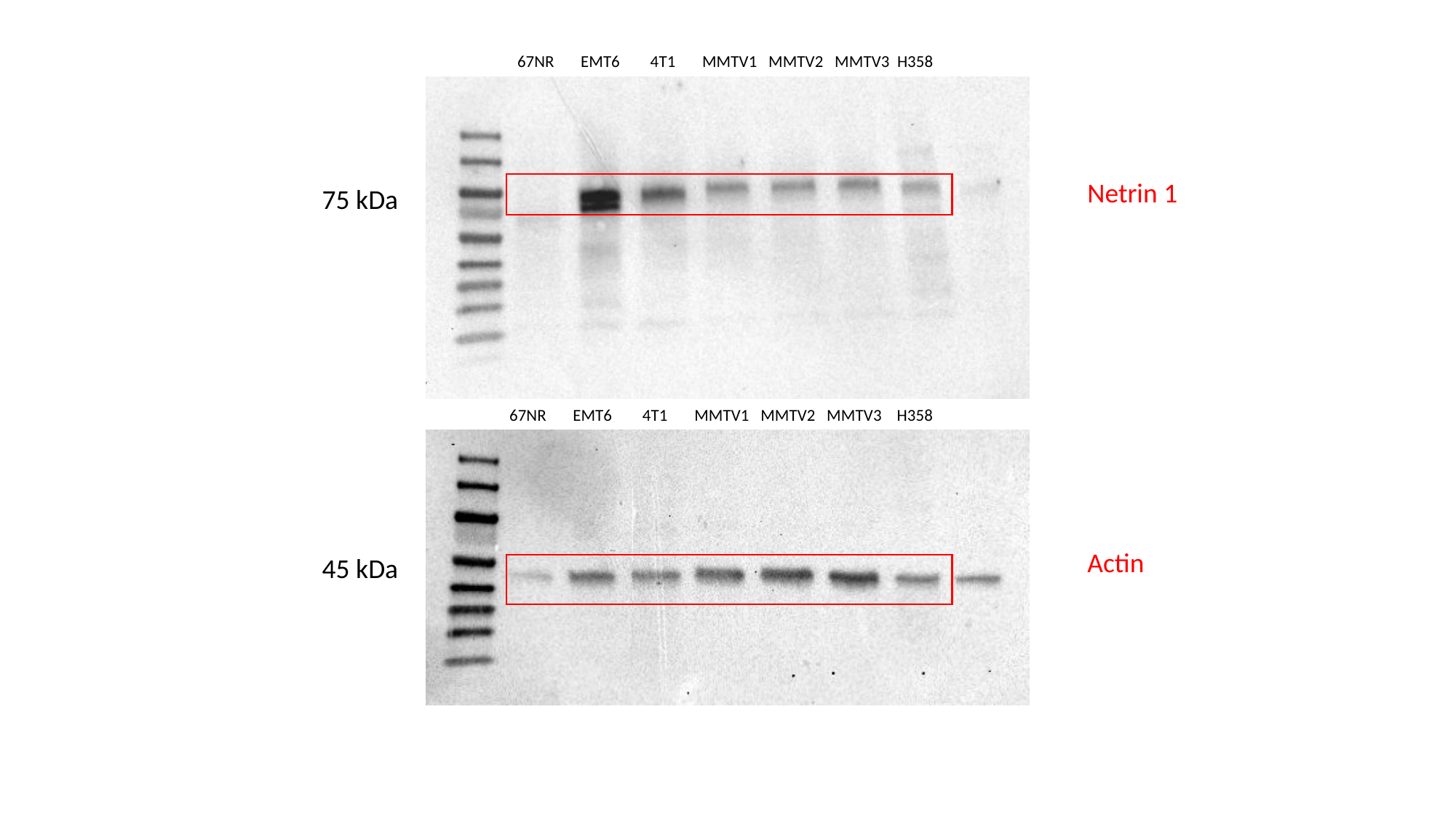

67NR EMT6 4T1 MMTV1 MMTV2 MMTV3 H358
Netrin 1
75 kDa
67NR EMT6 4T1 MMTV1 MMTV2 MMTV3 H358
Actin
45 kDa

Supplement: Supplementary file 8 — Source Data for Figure 5 [file EMMM-15-e16732-s007.zip › Figure 5/5B/Detection of netrin-1 expression, Blot.pptx]
