# Supplementary material for: From netrin‐1‐targeted SPECT/CT to internal radiotherapy for management of advanced solid tumors
Source: EMBO Mol Med. 2023 Mar 6;15(4):e16732. doi: 10.15252/emmm.202216732 (PMC10086585; doi:10.15252/emmm.202216732)
Supplement: Supplementary file 5 — Source Data for Figure 2 [file EMMM-15-e16732-s008.zip › Figure 2/2C/NP137 fragments, Blot.pptx]

## Slide 1
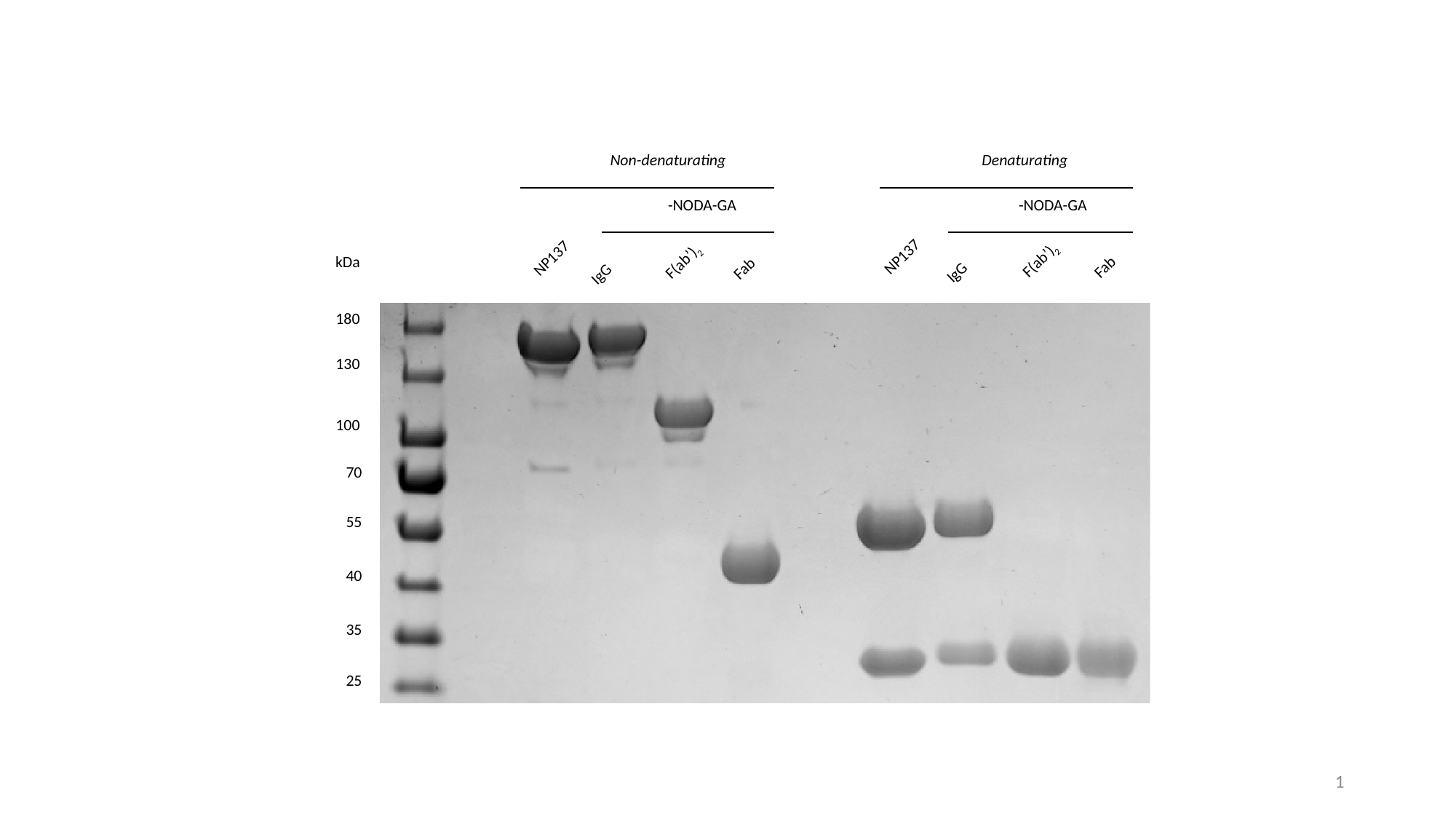

Non-denaturating
Denaturating
-NODA-GA
-NODA-GA
NP137
NP137
F(ab’)2
F(ab’)2
kDa
Fab
Fab
IgG
IgG
180
130
100
70
55
40
35
25
Verification of sizes (SDS-PAGE)
1
