# Supplementary material for: From netrin‐1‐targeted SPECT/CT to internal radiotherapy for management of advanced solid tumors
Source: EMBO Mol Med. 2023 Mar 6;15(4):e16732. doi: 10.15252/emmm.202216732 (PMC10086585; doi:10.15252/emmm.202216732)
Supplement: Supplementary file 6 — Source Data for Figure 3 [file EMMM-15-e16732-s006.zip › Figure 3/3G/Autoradiography of NP137-NODAGA-111, Radiography.pptx]

## Slide 1
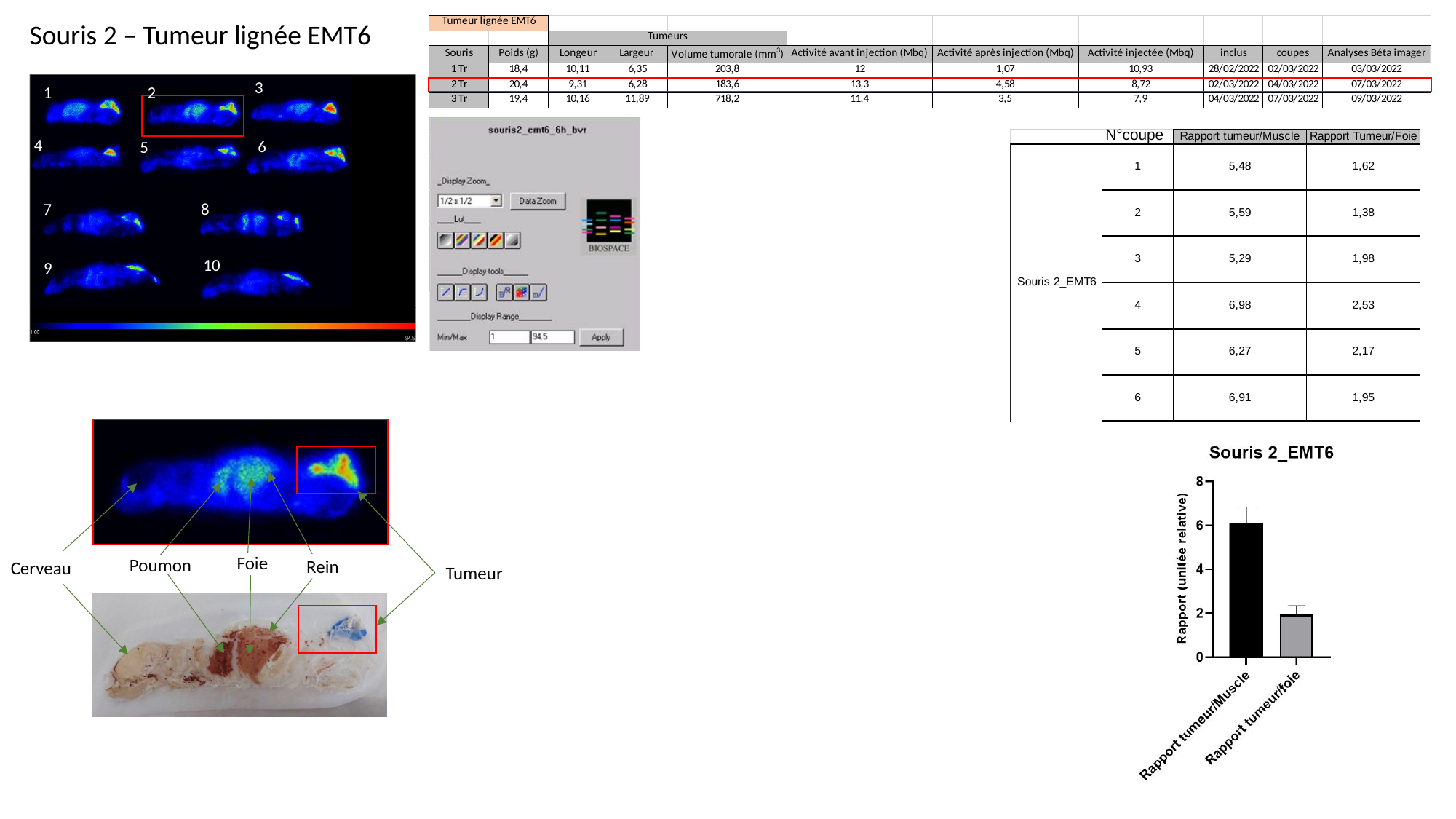

Souris 2 – Tumeur lignée EMT6
3
1
2
N°coupe
4
6
5
7
8
10
9
Foie
Poumon
Rein
Cerveau
Tumeur

## Slide 2
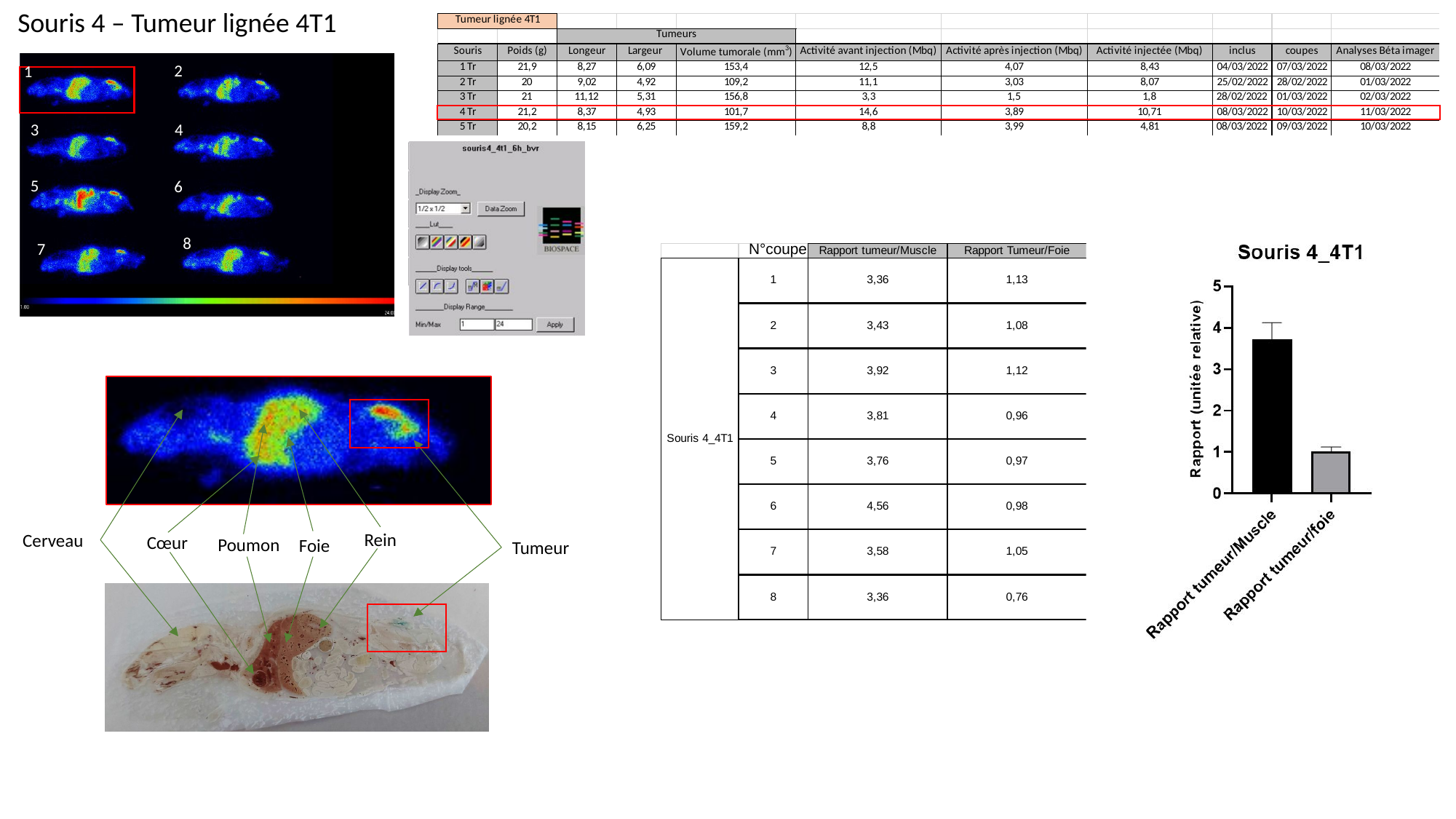

Souris 4 – Tumeur lignée 4T1
2
1
3
4
5
6
8
7
N°coupe
Rein
Cerveau
Cœur
Poumon
Foie
Tumeur
