# Supplementary material for: From netrin‐1‐targeted SPECT/CT to internal radiotherapy for management of advanced solid tumors
Source: EMBO Mol Med. 2023 Mar 6;15(4):e16732. doi: 10.15252/emmm.202216732 (PMC10086585; doi:10.15252/emmm.202216732)
Supplement: Supplementary file 9 [file EMMM-15-e16732-s005.pdf]

EMBO Molecular Medicine

From netrin-1-targeted SPECT/CT to internal radiotherapy for management of advanced solid tumors

David Kryza, Jennifer Wischhusen, Mathieu Richaud, Maëva Hervieu, Jacqueline Sidi Boumedine, Jean-Guy Delcros, Sophie Besse, Thomas Baudier, Pierre Alexandre Laval, Silvia Breusa, Erwan Boutault, Hugo Clermidy, Nicolas Rama, Benjamin Ducarouge, Mojgan Devouassoux-Shisheboran, Jean-Michel Chezal, Anne-Laure Giraudet, Thomas Walter, Patrick Mehlen2*, David Sarrut and Benjamin Gibert.
DOI: 10.15252/emmm.202216732

Corresponding authors: Patrick Mehlen (mehlen@lyon.fnclcc.fr) , Benjamin Gibert (benjamin.gibert@lyon.unicancer.fr), David Kryza (david.kryza@chu-lyon.fr), David Sarrut (david.sarrut@creatis.insa-lyon.fr)

Review Timeline:

Submission Date:	6th Sep 22
Editorial Decision:	13th Oct 22
Revision Received:	30th Dec 22
Editorial Decision:	23rd Jan 23
Revision Received:	8th Feb 23
Accepted:	10th Feb 23

Editor: Lise Roth

Transaction Report:

(Note: With the exception of the correction of typographical or spelling errors that could be a source of ambiguity, letters and reports are not edited. Depending on transfer agreements, referee reports obtained elsewhere may or may not be included in this compilation. Referee reports are anonymous unless the Referee chooses to sign their reports.)

13th Oct 2022

Dear Patrick,

Thank you for the submission of your manuscript to EMBO Molecular Medicine, and please accept my apologies for the delay in getting back to you as I was waiting for the last referee's report. We have now received feedback from two of the three reviewers who agreed to evaluate your manuscript. Given that referee #3 has not yet returned his/her report, and that both referees #1 and #2 provide similar recommendations, we prefer to make a decision now in order to avoid further delay in the process. As you will see from the reports below, the referees acknowledge the interest of the study and are overall supporting publication of your work pending appropriate revisions.
Should referee #3 provide a report, we will send it to you, with the understanding that we will not ask you extensive experiments in addition to the ones required in the enclosed reports from referee #1 and #2.

Addressing the reviewers' concerns in full will be necessary for further considering the manuscript in our journal, and acceptance of the manuscript will entail a second round of review. EMBO Molecular Medicine encourages a single round of revision only and therefore, acceptance or rejection of the manuscript will depend on the completeness of your responses included in the next, final version of the manuscript. For this reason, and to save you from any frustrations in the end, I would strongly advise against returning an incomplete revision.

Revised manuscripts should be submitted within three months of a request for revision; they will otherwise be treated as new submissions, except under exceptional circumstances in which a short extension is obtained from the editor.

When submitting your revised manuscript, please carefully review the instructions that follow below. We perform an initial quality control of all revised manuscripts before re-review; failure to include requested items will delay the evaluation of your revision.

We require:

1) A .docx formatted version of the manuscript text (including legends for main figures, EV figures and tables). Please make sure that the changes are highlighted to be clearly visible.

2) Individual production quality figure files as .eps, .tif, .jpg (one file per figure). For guidance, download the 'Figure Guide PDF' (https://www.embopress.org/page/journal/17574684/authorguide#figureformat).

3) At EMBO Press we ask authors to provide source data for the main and EV figures. Our source data coordinator will contact you to discuss which figure panels we would need source data for and will also provide you with helpful tips on how to upload and organize the files.
Numerical data should be provided as individual .xls or .csv files (including a tab describing the data). For blots or microscopy, uncropped images should be submitted (using a zip archive if multiple images need to be supplied for one panel). Additional information on source data and instruction on how to label the files are available at
.

4) A .docx formatted letter INCLUDING the reviewers' reports and your detailed point-by-point responses to their comments. As part of the EMBO Press transparent editorial process, the point-by-point response is part of the Review Process File (RPF), which will be published alongside your paper.

5) A complete author checklist, which you can download from our author guidelines (https://www.embopress.org/page/journal/17574684/authorguide#submissionofrevisions). Please insert information in the checklist that is also reflected in the manuscript. The completed author checklist will also be part of the RPF.

6) Please note that all corresponding authors are required to supply an ORCID ID for their name upon submission of a revised manuscript. ORCID identifiers are currently missing for D. Kryza and D. Sarrut. We note that you currently have together with you, a total of 4 co-corresponding authors. Is that correct? Do you confirm equal contribution of these 4 people, able to take full responsibility for the paper and its content? While there is no limit per se to the number of co-corresponding authors, 3 is rare, 4 even more so, and may not reflect as intended to the community.

7) It is mandatory to include a 'Data Availability' section after the Materials and Methods. Before submitting your revision, primary datasets produced in this study need to be deposited in an appropriate public database, and the accession numbers and database listed under 'Data Availability'. Please remember to provide a reviewer password if the datasets are not yet public (see https://www.embopress.org/page/journal/17574684/authorguide#dataavailability).
In case you have no data that requires deposition in a public database, please state so in this section. Note that the Data Availability Section is restricted to new primary data that are part of this study.

8) For data quantification: please specify the name of the statistical test used to generate error bars and P values, the number (n) of independent experiments (specify technical or biological replicates) underlying each data point and the test used to calculate p-values in each figure legend. The figure legends should contain a basic description of n, P and the test applied. Graphs must include a description of the bars and the error bars (s.d., s.e.m.). Please provide exact p values.

9) Our journal encourages inclusion of *data citations in the reference list* to directly cite datasets that were re-used and obtained from public databases. Data citations in the article text are distinct from normal bibliographical citations and should directly link to the database records from which the data can be accessed. In the main text, data citations are formatted as follows: "Data ref: Smith et al, 2001" or "Data ref: NCBI Sequence Read Archive PRJNA342805, 2017". In the Reference list, data citations must be labeled with "[DATASET]". A data reference must provide the database name, accession number/identifiers and a resolvable link to the landing page from which the data can be accessed at the end of the reference. Further instructions are available at .

10) We replaced Supplementary Information with Expanded View (EV) Figures and Tables that are collapsible/expandable online. A maximum of 5 EV Figures can be typeset. EV Figures should be cited as 'Figure EV1, Figure EV2" etc... in the text and their respective legends should be included in the main text after the legends of regular figures.
- For the figures that you do NOT wish to display as Expanded View figures, they should be bundled together with their legends in a single PDF file called *Appendix*, which should start with a short Table of Content. Appendix figures should be referred to in the main text as: "Appendix Figure S1, Appendix Figure S2" etc.
- Additional Tables/Datasets should be labeled and referred to as Table EV1, Dataset EV1, etc. Legends have to be provided in a separate tab in case of .xls files. Alternatively, the legend can be supplied as a separate text file (README) and zipped together with the Table/Dataset file.
See detailed instructions here:

11) The paper explained: EMBO Molecular Medicine articles are accompanied by a summary of the articles to emphasize the major findings in the paper and their medical implications for the non-specialist reader. Please provide a draft summary of your article highlighting
- the medical issue you are addressing,
- the results obtained and
- their clinical impact.
This may be edited to ensure that readers understand the significance and context of the research. Please refer to any of our published articles for an example.

12) For more information: There is space at the end of each article to list relevant web links for further consultation by our readers. Could you identify some relevant ones and provide such information as well? Some examples are patient associations, relevant databases, OMIM/proteins/genes links, author's websites, etc...

13) Author contributions: CRediT has replaced the traditional author contributions section because it offers a systematic machine readable author contributions format that allows for more effective research assessment. Please remove the Authors Contributions from the manuscript and use the free text boxes beneath each contributing author's name in our system to add specific details on the author's contribution. More information is available in our guide to authors.

14) Conflict of interest: We updated our journal's competing interests policy in January 2022 and request authors to consider both actual and perceived competing interests. Please review the policy https://www.embopress.org/competing-interests and update your competing interests if necessary.

15) Every published paper now includes a 'Synopsis' to further enhance discoverability. Synopses are displayed on the journal webpage and are freely accessible to all readers. They include a short stand first (maximum of 300 characters, including space) as well as 2-5 one-sentences bullet points that summarizes the paper. Please write the bullet points to summarize the key NEW findings. They should be designed to be complementary to the abstract - i.e. not repeat the same text. We encourage inclusion of key acronyms and quantitative information (maximum of 30 words / bullet point). Please use the passive voice. Please attach these in a separate file or send them by email, we will incorporate them accordingly.

Please also suggest a striking image or visual abstract to illustrate your article as a PNG file 550 px wide x 300-600 px high.

16) As part of the EMBO Publications transparent editorial process initiative (see our Editorial at http://embomolmed.embopress.org/content/2/9/329), EMBO Molecular Medicine will publish online a Review Process File (RPF) to accompany accepted manuscripts.
In the event of acceptance, this file will be published in conjunction with your paper and will include the anonymous referee reports, your point-by-point response and all pertinent correspondence relating to the manuscript. Let us know whether you agree with the publication of the RPF and as here, if you want to remove or not any figures from it prior to publication.
Please note that the Authors checklist will be published at the end of the RPF.

EMBO Molecular Medicine has a "scooping protection" policy, whereby similar findings that are published by others during review or revision are not a criterion for rejection. Should you decide to submit a revised version, I do ask that you get in touch after three months if you have not completed it, to update us on the status.

I look forward to receiving your revised manuscript.

With kind regards,

Lise

Lise Roth, PhD
Senior Editor
EMBO Molecular Medicine

***** Reviewer's comments *****

Referee #1 (Comments on Novelty/Model System for Author):

Although, the different models were used to test the theranostic potential of anti netrin1 antibody, the choice of models is not always clear. see also section remarks for more information.

Referee #1 (Remarks for Author):

Kryza et al. investigate the theranostic potential of anti netrin1 by using available anti-netrin1 monoclonal antibody. They investigate the biomarker potential by combing it to indium-111 and detecting it with SPECT and the therapeutic potential by combing it lutetium 117. The manuscript is well written, however the following points should be considered.
The authors use different models to test the detection and therapeutic potential. However, the choice of models or cell lines is not clear. Is there a correlation with netrin1 expression and choice of cell lines? What is the expression of netrin1 in H358 cells? And does the degree of expression correlates with signal intensity and/or response to NP137 (NP137-111/NP137-177)?
It looks like in EMT6 cells the signal to other organs (heart, kidney, uterus) is higher than the tumor (Fig SF3), why is this the case? And what does it say about the specificity and response to treatment? Also the authors showed in their previous paper that the NP137 can be used as a target for other netrins, what is the consequence of this non-specificity? Can the authors also provide the biodistribution data for H358 mice?
It is also not clear, why the authors opted to test several chelators for their radionuclide? Why not use for both the SPECT and radiation therapy setting the same chelator? Can the author elaborate on this? Is it possible to use one type of radionuclide that can be used both for imaging and as therapeutic agent?
The authors previously mention that NP137 (NP137-DOTA) shows anti-tumor activity, however, in all the tested models this is lacking; how can this be explained? Did the authors checked the tumor tissue for netrin1 expression, is there an inhibition? NP137 is known to restore apoptosis in the tissues, but only a limited increase in apoptotic cells can be seen in Figure 4E in NP137 conditions. Is it because of the selected model? What is the increase in apoptosis in the other tested models?
The apoptotic fraction is given as number of cells/mm^2, can the authors provide percentages? This would make the interpretation easier.
Minor:
Reference to Fig SF3 is missing in the text.
Elaborate how the analysis of autoradiography was done in Figure 3H.
Elaborate which is defined as an end point in Kaplan-Meier curves in Figure 4.

Referee #2 (Comments on Novelty/Model System for Author):

Overall, this is a strong manuscript. It is well written and the data are novel, well presented, and mostly critically discussed. I have several suggestions that should help to improve the paper.

In summary, I recommend the publication of the article if the authors are able to address my queries.

Referee #2 (Remarks for Author):

The paper entitled "From netrin-1-targeted SPECT/CT to internal radiotherapy for management of advanced solid tumors" is a well-written, clear and concise article on the use of netrin-1 as a potential target for radiotherapy with utility as a theranostic reagent.

Netrin-1 is a protein involved in axonal guidance in nervous system development. It is also implicated in cell adhesion, cell motility, proliferation and also in the protection of cells from apoptosis. For these reasons it is being considered as a target for the treatment of cancers and a human antibody to netrin-1 is in clinical development as a blocker of the netrin-1/dependence receptors such as DCC and UNCSH.

In the current paper the authors first provide evidence that netrin-1 is a soluble factor secreted by tumour cells that binds to particular receptors in the tumor microenvironment. Here the authors show that netrin-1 is bound to matrix-forming proteins laminin-1, lumican, fibronectin leucine rich transmembrane protein, integrin alpha6beta4, glypican-1 and vitronectin.
They then test the tumor localization and biodistribution of 111In-labelled intact anti-netrin-1 antibody (NP137) and its F(ab')2 and Fab fragments. Using netrin-1 positive syngeneic tumor models- 4T1, EMT6 and human xenograft model H358 it was shown that the intact NP137 antibody had specific tumour uptake of 10-28% inj dose/gm compared to the antibody fragments and low uptake in netrin negative 67NR tumors.

Comments for further improvements of the submitted manuscript:

Please label molecular weight standards in Fig 2c.

In these studies, even though high accumulation of label is seen in tumour, uptake in liver, heart spleen are still quite significant relative to tumour. Is this not a major limitation as a potential theranostic agent? The authors need to discuss this appropriately.

Are the authors' findings restricted to only certain types of tumors? Clearly different levels of tumor uptake is seen in the different models. Is this related to the amount of netrin-1 expressed by the individual tumor? Different accumulation in TME? Again, the authors need to discuss this or even better provide additional data.

Fig 2e. It might be useful to show numerical affinities of unmodified Ab and fragments (from Sup 1e) in the same Table.
As an extension to the biodistribution studies the authors labeled the NP137 antibody with the beta-emitter 177Lu for use as a therapeutic agent for treatment of EMT-6, 4T1 and H358. Even though significant tumor inhibition was observed the results are not impressive considering therapeutic effects of antibody drug conjugates. Please consider limitations - how was the radioactive dose determined (were dose response studies performed?). Would multiple doses enable better therapeutic effect without toxicity?
The authors indicate that the labelled antibody was stable in serum however, the materials and methods indicate that stability was determined in PBS!

Assessment of therapeutic efficacy in the more "realistic" MMTV-NeuT transgenic model is very appropriate. The characterization of the netrin-1 expression levels biochemically and immunohistochemically in the mammary tumors is excellent. The levels in the tumors were not as high as in EMT6 and 4T1 and could be due to the lower expression of netrin-1. Even though therapeutic effects were seen and localization was evident activity in liver and spleen was large.

I think the introduction could be improved with reference to theranostics and also some information about immunoscintigraphy and radiotherapy with radioisotopes.

Also, it is worth emphasising that netrin-1 is a potential target for antibody drug conjugates targeting the tumour microenvironment in combination with targeted extracellular drug release. This should be discussed in the context of targeting antigens such as fibroblast activatory protein [Lindner et al., Targeting of activated fibroblasts for imaging and therapy. EJNMMI Radiopharmacy and Chemistry 4: 16 (2019); Fabre et al., OMTX705, a Novel FAP-Targeting ADC Demonstrates Activity in Chemotherapy and Pembrolizumab-Resistant Solid Tumor Models. Clin Cancer Res 2020;26:3420-30], tenascin-C [Dal Corso et al., A Non-internalizing Antibody-Drug Conjugate Based on an Anthracycline Payload Displays Potent Therapeutic Activity in vivo. J Control Release. 2017 Oct 28; 264: 211-218], fibronectin and platelets [May Lin Yap et al. Activated platelets in the tumor microenvironment for targeting of antibody-drug conjugates to tumors and metastases. Theranostics 9:1154-1169 (2019)].

Referee #1 (Comments on Novelty/Model System for Author):

Although, the different models were used to test the theranostic potential of anti netrin1 antibody, the choice of models is not always clear. see also section remarks for more information.

We apologize for the lack of clarity in our manuscript and we have now clarified the points that were raised. Concerning the models used, we used four models based on cancer cells engraftment (three of them syngeneic, one was xenograft), 3 positives for netrin-1 expression: H358, 4T1 and EMT-6 which express netrin-1 at different levels and 67NR which does not express netrin-1 (**see new figure extended view 3E**) and which serves as a negative incorporation control. We also used a model of spontaneous mammary tumorigenesis the MMTV-NeuT that presents the advantage to mimic intrinsic tumor progression **and is now presented in Figure 5B**. This model is more physiological and pathophysiological for the intrinsic contribution of blood and lymphatic vessels, immune infiltrate or fibroblasts.

The use of these models has been clarified in the text of the new version of the manuscript.

Referee #1 (Remarks for Author):

Kryza et al. investigate the theranostic potential of anti netrin1 by using available anti-netrin1 monoclonal antibody. They investigate the biomarker potential by combing it to indium-111 and detecting it with SPECT and the therapeutic potential by combing it lutetium 117. The manuscript is well written; however, the following points should be considered.
We thank Reviewer 1 for her/his kind comments on our study and we hope that we have responded to all of his/her concerns.

The authors use different models to test the detection and therapeutic potential. However, the choice of models or cell lines is not clear. Is there a correlation with netrin1 expression and choice of cell lines? What is the expression of netrin1 in H358 cells? And does the degree of expression correlates with signal intensity and/or response to NP137 (NP137-111/NP137-177)?
A new immunoblot analysis has now been added as a new **extended View 1B** which gathers in the same figure netrin-1 expression of all our models. Netrin-1 levels can predict response which seems to be the case in our three models: the more the tumors express netrin-1, the more the incorporation of the radiolabeled antibody is important *i.e.*, 25% of the dose for 4T1 which are highly positive, 15% and 8-10% for EMT-6 and H358 intermediate and less than 3% for 67NR which does not express the target. The link between netrin-1 expression and the therapeutic effects is less marked but there are obviously other points to take into account such as the intrinsic radio resistance of the tumor cell lines, the perfusion through the blood and lymphatic vessels. These points are now addressed in the discussion section.

It looks like in EMT6 cells the signal to other organs (heart, kidney, uterus) is higher than the tumor (Fig SF3), why is this the case? And what does it say about the specificity and response to treatment? Also, the authors showed in their previous paper that the NP137 can be used as a target for other netrins, what is the consequence of this non-specificity? Can the authors also provide the biodistribution data for H358 mice?
As raised by the referee we already described that the NP137 can bind netrin-1 but also netrin-3. Netrin-1 and -3 have mutually exclusive expressions in many cancer lines and are for example only co-expressed in 4 out of 675 cancer lines (Jiang et al, 2021). The set of models tested therefore does not express netrin-3 which strongly reduces a possible interference in our results. Moreover, netrin-1 and -3 are not/limitedly expressed in adult tissues except in the central nervous system. Having chosen a whole antibody (and not a Fab or F(ab)'2 fragment) should prevent crossing the blood brain barrier and avoid possible toxicity. Concerning other netrins, there is no conservation of the NP137 epitope in the other netrin-4, -5 -G1 and -G2 (Jiang et al, 2021). A tissue cross reactivity study was performed during the reglementary preclinical development of the NP137 (Grandin et al., Cancer Cell, 2016) and did not reveal any non-specific organ/tissues binding in human specimens (with the exception of the brain). Phase I data performed on patient with advanced solid cancer in monotherapy also revealed an excellent safety profile with basically no toxicity of the compound (Cassier et al. 2019).

Radiolabeled NP137 is injected into the tail vein of mice and diffuses *via* the bloodstream. In the field of nuclear imaging, radiolabeled compounds are often - if not always- found in organs that are highly vascularized like the liver, spleen and lungs, or with high blood flow such as the heart. Concerning the detection in the kidneys, the free chelated radionuclides and/or metabolites are eliminated by the urine and it is normal to find them during elimination. The uterus was slightly marked during this experiment but we did not find this accumulation in our other experiments with other models. The non-specificity in general is of course a problem but it is largely counterbalanced by the gain in general survival of the patients and solutions have already been put in place in the clinic such as injecting patients with an amino acid solution to desaturate the radio-elements from the kidneys for example in Lutathera® therapy, or injecting the cold antibody before the radio-element treatment to saturate the Fc receptors in the liver and the hematopoietic cells.
The H358 biodistribution has been performed to respond to this question and is now added in the **new Extended View 3E**.
Specificity and toxicity are now discussed in the new version of the manuscript.

It is also not clear, why the authors opted to test several chelators for their radionuclide? Why not use for both the SPECT and radiation therapy setting the same chelator? Can the author elaborate on this? Is it possible to use one type of radionuclide that can be used both for imaging and as therapeutic agent?
As noted by reviewer 1, we used multiple chelators, DOTA and NODAGA in our manuscript. When we started the study in 2018, we thought we were only doing a netrin-1 imaging study using NODAGA to chelate ^{111}In. The therapy part with Lutetium 177 came next, once the results showed a very high tumor uptake (higher than 25% in 4T1 cells) which makes NP137 a molecule of interest according to the standards even with clinically approved molecules which are more frequently around 10% tumor uptake in animals.
For clinical radiotherapy, DOTA is the standard chelator. In order to facilitate clinical translation of our approach, we therefore opted for DOTA as the chelator for Lutetium 177. In order to understand whether the chelator affects radiotherapy, we compared differences between NODAGA and DOTA, and we could not observe any additional effect of either chelator (**Expended View 4B, C**). The reviewer is totally right when she/he highlighted that for the future development and in particular the clinical passage, we should try to characterize a single chelator to make a simple isotope switch (between Indium 111 and Lutetium 177) and thus develop at the clinical GMP grade only one molecule. This point is now discussed in the new version of the manuscript.
With regard to the question whether one type of radionuclide can be used both for imaging and therapy, we would like to clarify, that the radiation energy emitted by Indium 111 and Lutetium 177 is fundamentally different. There is unfortunately not one perfect radionuclide which would allow to perform high resolution SPECT imaging and high energy radiotherapy at the same time (Lutetium 177 can be imaged but with a very poor resolution).

The authors previously mention that NP137 (NP137-DOTA) shows anti-tumor activity, however, in all the tested models this is lacking; how can this be explained? Did the authors check the tumor tissue for netrin1 expression, is there an inhibition? NP137 is known to restore apoptosis

in the tissues, but only a limited increase in apoptotic cells can be seen in Figure 4E in NP137 conditions. Is it because of the selected model? What is the increase in apoptosis in the other tested models?
Reviewer 1 is totally right when he/she points out the lack of efficacy of NP137-DOTA in our 4T1, EMT6, H358 models. However, we used here concentrations of NP137 that are 20 folds lower and we injected NP137 only once (While in the previous studies investigating anti-tumor activity of netrin-1 interfering agent, it was injected two or three times a week). We have corrected this lack of clarity in the new version of the text.

The apoptotic fraction is given as number of cells/mm², can the authors provide percentages? This would make the interpretation easier.
This point has now been addressed in the revised version of the manuscript.

Minor:

Reference to Fig SF3 is missing in the text.
We apologize for this mistake. The text has been corrected accordingly.

Elaborate how the analysis of autoradiography was done in Figure 3H.
This has been amended in the revised form.

Elaborate which is defined as an end point in Kaplan-Meier curves in Figure 4.
As requested by reviewer 1, this point has been added in the figure legend of **Figure 4**. It is the ethical end point of tumor growth (2000 mm^3) and not the actual death of animals, as stipulated in the French and European ethical guidelines.

Referee #2 (Comments on Novelty/Model System for Author):

Overall, this is a strong manuscript. It is well written and the data are novel, well presented, and mostly critically discussed. I have several suggestions that should help to improve the paper.

In summary, I recommend the publication of the article if the authors are able to address my queries.
We thank Reviewer 2 for his/her kind comments on our study and thank him/her for recommending the publication of our work. We hope we will respond to all of his/her concerns to improve the paper.

Referee #2 (Remarks for Author):

The paper entitled "From netrin-1-targeted SPECT/CT to internal radiotherapy for management of advanced solid tumors" is a well-written, clear and concise article on the use of netrin-1 as a potential target for radiotherapy with utility as a theranostic reagent.

Netrin-1 is a protein involved in axonal guidance in nervous system development. It is also implicated in cell adhesion, cell motility, proliferation and also in the protection of cells from apoptosis. For these reasons it is being considered as a target for the treatment of cancers and a human antibody to netrin-1 is in clinical development as a blocker of the netrin-1/dependence receptors such as DCC and UNCSH. In the current paper the authors first provide evidence that netrin-1 is a soluble factor secreted by tumour cells that binds to particular receptors in the tumor microenvironment. Here the authors show that netrin-1 is bound to matrix-forming proteins laminin-1, lumican, fibronectin leucine rich transmembrane protein, integrin alpha6beta4, glypican-1 and vitronectin. They then test the tumor localization and biodistribution of 111In-labelled intact anti-netrin-1 antibody (NP137) and its F(ab')2 and Fab fragments. Using netrin-1 positive syngeneic tumor models- 4T1, EMT6 and human xenograft model H358 it was shown that the intact NP137 antibody had specific tumour uptake of 10-28% inj dose/gm compared to the antibody fragments and low uptake in netrin negative 67NR tumors.
We thank Reviewer 2 for her/his kind comments on our manuscript.

Comments for further improvements of the submitted manuscript:

Please label molecular weight standards in Fig 2c.
The labeling of **Figure 2C** is now provided in the new version of the manuscript.

In these studies, even though high accumulation of label is seen in tumour, uptake in liver, heart spleen are still quite significant relative to tumour. Is this not a major limitation as a potential theranostic agent? The authors need to discuss this appropriately.
This is a very interesting point. Antibodies in general diffuse through the bloodstream (in our case injected into the tail vein of the animals). The presence of the compound in the heart, the spleen and the liver, which are all three organs with a high blood flow, is therefore normal, and it is valid for any antibody, radio-coupled or not. Concerning toxicity, similar molecules have already been approved for use in humans. The liver is a relatively radio-resistant organ, the toxicities detected in humans are essentially medullary with aplasia related to the cytotoxicity of the compounds. Although real, these effects, reversible by clinical management, are largely outweighed by the clinical anti-tumor gains in patient survival.
This point is now addressed in the discussion part of the manuscript.

Are the authors' findings restricted to only certain types of tumors? Clearly different levels of tumor uptake is seen in the different models. Is this related to the amount of netrin-1 expressed by the individual tumor? Different accumulation in TME? Again, the authors need to discuss this or even better provide additional data.
As pointed out by the reviewer 2, in our study we showed that our compound was active in several tumor types (breast and lung cancer models). In theory, our NP137-^{177}Lu molecule could be effective on all tumor types expressing netrin-1. The clinical reality is of course much more complex. Some cancer subtypes are more intrinsically radio-resistant than others (like kidney cancer). Others are poorly perfused tumors with strong infiltration of cancer-associated fibroblasts that form a physical barrier around the tumor cells and prevent the diffusion of the

compounds. We will therefore try to focus on other tumor types for future development and in particular for studies in humans. The advantage of coupling our therapy with imaging is that only patients with fixating tumors will be treated, which should increase the number of anti-cancer responses observed.

Fig 2e. It might be useful to show numerical affinities of unmodified Ab and fragments (from Sup 1e) in the same Table.
The figures have been fused in the new version of the manuscript **in a new Figure 2E**.

As an extension to the biodistribution studies the authors labeled the NP137 antibody with the beta-emitter ^{177}Lu for use as a therapeutic agent for treatment of EMT-6, 4T1 and H358. Even though significant tumor inhibition was observed the results are not impressive considering therapeutic effects of antibody drug conjugates. Please consider limitations - how was the radioactive dose determined (were dose response studies performed?). Would multiple doses enable better therapeutic effect without toxicity?
In radioimmunology, the standard is to treat only once; primarily to avoid toxicity in animals and to avoid suffering. Thus, toxicity in animals appear mainly in hematopoietic cells and usually dissipate in 3/4 weeks. The models used in our study such as 4T1 are aggressive and the transplanted animals reach the ethical endpoint (2000 mm^3 tumors) in 3 weeks, which therefore does not allow for multiple treatments. Multiple dose studies will be performed on less aggressive predisposing models growing in several months to avoid toxicity. In the patient, cycles of 2 radioimmunotherapy treatments are generally performed to achieve maximum efficiency.

The authors indicate that the labelled antibody was stable in serum however, the materials and methods indicate that stability was determined in PBS!
We apologize for this mistake. The text has been corrected accordingly.

Assessment of therapeutic efficacy in the more "realistic" MMTV-NeuT transgenic model is very appropriate. The characterization of the netrin-1 expression levels biochemically and immunohistochemically in the mammary tumors is excellent. The levels in the tumors were not as high as in EMT6 and 4T1 and could be due to the lower expression of netrin-1. Even though therapeutic effects were seen and localization was evident activity in liver and spleen was large.
We thank reviewer 2 for his/her comments on the suitability of our models.

I think the introduction could be improved with reference to theranostics and also some information about immunoscintigraphy and radiotherapy with radioisotopes. Also, it is worth emphasising that netrin-1 is a potential target for antibody drug conjugates targeting the tumour microenvironment in combination with targeted extracellular drug release. This should be discussed in the context of targeting antigens such as fibroblast activatory protein [Lindner et al., Targeting of activated fibroblasts for imaging and therapy. EJNMMI Radiopharmacy and Chemistry 4: 16 (2019); Fabre et al., OMTX705, a Novel FAP-Targeting ADC Demonstrates Activity in Chemotherapy and Pembrolizumab-Resistant Solid Tumor Models. Clin Cancer Res 2020;26:3420-30], tenascin-C [Dal Corso et al., A Non-internalizing Antibody-Drug Conjugate

Based on an Anthracycline Payload Displays Potent Therapeutic Activity in vivo. J Control Release. 2017 Oct 28; 264: 211-218], fibronectin and platelets [May Lin Yap et al. Activated platelets in the tumor microenvironment for targeting of antibody-drug conjugates to tumors and metastases. Theranostics 9:1154-1169 (2019)].

As requested by the reviewer 2, the introduction has been modified to include these comments on the targeting of the tumor microenvironment by ADCs and all the proposed references.

References:

- Cassier P, Eberst L, Garin G, Courbebaisse Y, Terret C, Robert M, Frenel J-S, Depil S, Delord J-P, Perol D, et al (2019) A first in human, phase I trial of NP137, a first-in-class antibody targeting netrin-1, in patients with advanced refractory solid tumors. Annals of Oncology 30: v159

- Ducarouge B, Redavid AR, Victoor C, Fonseca A, Hervieu M, Bergé R, Lengrand J, Vieugué P, Neves D, Goddard I, Richaud M, Laval PA, Rama N, Goldschneider D, Paradisi A, Gourdin N, Chabaud S, Treilleux I, Gadot N, Ray-Coquart I, Decaudin D, Némati F, Marangoni E, Mery-Lamarche E, Génestie C, Tabone-Eglinger S, Devouassoux M, Moore KJ, Gibert B, Mehlen P and Bernet A. *Cell* Netrin-1 blockade inhibits tumor associated Myeloid-derived suppressor cells, cancer stemness and alleviates resistance to chemotherapy and immune checkpoint inhibitor. *Death and Differentiation* 2022 In press.

- Grandin M, Meier M, Delcros JG, Nikodemus D, Reuten R, Patel TR, Goldschneider D, Orriss G, Krahn N, Boussouar A, *et al* (2016) Structural Decoding of the Netrin-1/UNC5 Interaction and its Therapeutical Implications in Cancers. *Cancer Cell* 29: 173–185

- Jiang S, Richaud M, Vieugué P, Rama N, Delcros JG, Siouda M, Sanada M, Redavid AR, Ducarouge B, Hervieu M, Breusa S, Manceau A, Gattolliat CH, Gadot N, Combaret V, Neves D, Ortiz-Cuaran S, Saintigny P, Meurette O, Walter T, Janoueix-Lerosey I, Hofman P, Mulligan P, Goldshneider G, Mehlen P and Gibert B.Targeting netrin-3 in Small Cell Lung Cancer and Neuroblastoma. *EMBO Mol Med.* 2021 Mar 15:e12878. doi: 10.15252/emmm.202012878.

We are grateful to the reviewers for their comments, which we believe have strengthened the manuscript.

If we should send additional information, please let me know. We thank you in advance for your consideration of the revised manuscript.

Sincerely yours,

Patrick Mehlen, Ph.D.
Member of the French Academy of Sciences

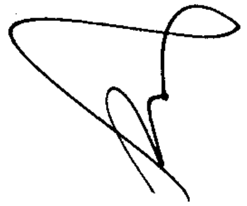

23rd Jan 2023

Dear Patrick,

Thank you for the submission of your revised manuscript to EMBO Molecular Medicine. We have received the enclosed reports from the two referees who re-reviewed your study. As you will see, they are now supportive of publication, and we will therefore be able to accept your manuscript once the following points will be addressed:

1/ Referees' comments:
- Please address the remaining minor concerns from referee #1.

2/ Main manuscript text:
- Please address the queries from our data editors in track changes mode in the main manuscript file labelled 'Data edited MS file'. Please use this file for any further modification (in track changes mode, remove the red text).
- Please provide up to 5 keywords.
- Author contributions: We note that you currently have together with you, a total of 4 co-corresponding authors. Is that correct? Do you confirm equal contribution of these 4 people, able to take full responsibility for the paper and its content? While there is no limit per se to the number of co-corresponding authors, 3 is rare, 4 even more so, and may not reflect as intended to the community.
- Materials and methods:
o This section should be place after the Discussion.
o Cell culture: please indicate whether the cells were authenticated and tested for mycoplasma contamination.
o Western blots: please provide the antibody dilutions.
o Statistics: please include a statement about randomization, blinding and exclusion criteria.
o The Data Availability section should be place after the Materials and Methods.
- Please replace "Conflicts of interest" by "Disclosure statement and competing interests". We updated our journal's competing interests policy in January 2022 and request authors to consider both actual and perceived competing interests. Please review the policy https://www.embopress.org/competing-interests and update your competing interests if necessary.
- Acknowledgements: please make sure that the funding information provided in the manuscript and the submission system match.

2/ Figures:
- We added the EV figure legends to the manuscript. The figures should be renamed "Figure EV1" etc. in the figure files. The heading of the legends should be changed to "Expanded View Figure Legends".

3/ Thank you for providing Source Data. Please also upload the completed SD checklist. There is a Biodistribution file that we were not sure to which figure to attribute, could you please clarify?

4/ Checklist:
- Please fill in the section "Newly Created Materials"
- Please fill the section about blinding (statistics)
- Please fill in the Data availability section, primary datasets ("The study includes no data deposited in external repositories").

5/ I included some amendments to your Paper Explained. Please let me know if you agree with the following or amend as you see fit:

Problem
In oncology, despite their name, targeted therapies are often given without patient pre-screening. Immunohistochemistry, the gold standard for tumor diagnosis, is largely conducted on the primary tumor and this diagnosis may vary or evolve once metastases appear at a much later stage, including when no primary tumor is present anymore. Near-real-time diagnostic approaches are therefore urgently needed.
Results
Using netrin-1 and its blocking antibody as a proof-of-concept for tumor therapy, we were able to visualize this tumor cell-specific protein by nuclear imaging. We used this finding to design a near-real-time companion test to netrin-1 therapy. Tumor incorporation of anti-netrin-1 being highly efficient, we created a second therapeutic molecule by fusing this antibody to Lutecium 177, a radioelement capable of destroying cancer cells upon exposure. Mechanistically, we demonstrated that netrin-1, although secreted, is not as diffusible as previously described but remains trapped in the extracellular matrix and microenvironment of cancer cells, justifying its potential as a target for vectorized radiotherapy.
Impact
Netrin-1 and more generally matrix embedded proteins are promising targets for both molecular imaging and radio-immunotherapy.

6/ I slightly edited your synopsis text to fit our format, please let me know if you agree with the following or amend as you see fit:
During development, netrin-1 is released and diffusible in the microenvironment. We showed that in cancer, netrin-1 accumulates in the extracellular matrix. We used this property to follow its in vivo accumulation by radiolabeling and to target netrin-1-expressing tumor cells with radioimmunotherapy.
• In cancer tissues, netrin-1 is secreted but not diffusible.
• Netrin-1 interacts with many components of the extracellular matrix and accumulates in the tumor intercellular space.
• Anti-netrin-1 monoclonal antibody can be radiolabeled to detect in vivo the presence of netrin-1 as a diagnostic test.
• Anti-netrin-1 monoclonal antibody can be conjugated with Lutecium 117 to cargo the radioelement and destroy tumor cells in vivo.

7/ As part of the EMBO Publications transparent editorial process initiative (see our Editorial at http://embomolmed.embopress.org/content/2/9/329), EMBO Molecular Medicine will publish online a Review Process File (RPF) to accompany accepted manuscripts.
This file will be published in conjunction with your paper and will include the anonymous referee reports, your point-by-point response and all pertinent correspondence relating to the manuscript. Let us know whether you agree with the publication of the RPF and as here.
Please note that the Authors checklist will be published at the end of the RPF.

I look forward to receiving your revised manuscript.

With kind regards,

Lise

Lise Roth, PhD
Senior Editor
EMBO Molecular Medicine

***** Reviewer's comments *****

Referee #1 (Comments on Novelty/Model System for Author):

The authors sufficiently explained the use of the chosen models.

Referee #1 (Remarks for Author):

The authors have addressed the raised points.
However, it is not clear on which figure the authors based their observation about the correlation between expression of netrin-1 levels and radio labeled antibody uptake? The extended figure 1B which shows the expression of netrin-1 levels of all the models,shows a lower expression in 4T1 cells (cells with highest uptake) compared to EMT-6 and H358 cells. Could the authors check this statement? In addition, the author state in point by point their reply that they discuss the use of single chelators, can the authors check if this is missing in the revised manuscript?

Referee #2 (Remarks for Author):

The authors have addressed my previous comments appropriately and have substantially improved the manuscript.

The authors have addressed the raised points.
However, it is not clear on which figure the authors based their observation about the correlation between expression of netrin-1 levels and radio labeled antibody uptake? The extended figure 1B which shows the expression of netrin-1 levels of all the models, shows a lower expression in 4T1 cells (cells with highest uptake) compared to EMT-6 and H358 cells. Could the authors check this statement?

The referee is right when he/she points out that the netrin-1 levels are not as clear as we have interpreted them. The following sentence was corrected in the latest version of the manuscript as followed:
“This tumor incorporation was also linked with netrin-1 levels i.e., the more netrin-1 was expressed in the tumor model, the stronger was the radioactive signal enhancement in the tumor: i.e., 20-25% of the dose for 4T1 which are highly positive for netrin-1, 15% and 8-10%

for EMT6 and H358 with intermediate expression levels, and less than 3% for 67NR tumors, which do not express the target.” has been replaced by:
“This tumor uptake is globally linked also to netrin-1 protein levels, the antibody is not incorporated by 67NR tumors that do not express the target, whereas it is concentrated in the 3 tumor lines tested, that express it at different levels. Other parameters will have to be analyzed in future studies; tumor uptake is higher in 4T1 than in EMT6 which expresses more netrin-1 *in vitro*. We can imagine that the composition of the extracellular matrix plays a role in the retention and diffusion of netrin-1.”

In addition, the author state in point by point their reply that they discuss the use of single chelators, can the authors check if this is missing in the revised manuscript?
Although our study did not reveal a clear difference between DOTA and NODAGA during the therapeutic phase, the choice of a single chelator as point by the reviewer, will be important for future clinical development, in order to perform imaging and therapeutic phases with the same molecule in a single GMP batch. We apologize for this oversight, so the following sentence has been added to the discussion section: “Although our study showed no clear difference between DOTA and NODAGA, the choice of a single chelator will be important for faster clinical development to perform the imaging and therapy phases with the same NP137-DOTA/NODAGA GMP batch."

10th Feb 2023

Dear Patrick,

Thank you for sending your revised manuscript.
I am pleased to inform you that your manuscript is accepted for publication and is now being sent to our publisher to be included in the next available issue of EMBO Molecular Medicine!

Congratulations on your interesting work,

With kind regards,
Lise

Lise Roth, Ph.D
Senior Editor
EMBO Molecular Medicine

Follow us on Twitter @EmboMolMed
Sign up for eTOCs at embopress.org/alertsfeeds

*** *** *** IMPORTANT INFORMATION *** *** ***

SPEED OF PUBLICATION
The journal aims for rapid publication of papers, using using the advance online publication "Early View" to expedite the process: A properly copy-edited and formatted version will be published as "Early View" after the proofs have been corrected. Please help the Editors and publisher avoid delays by providing e-mail address(es), telephone and fax numbers at which author(s) can be contacted.

Should you be planning a Press Release on your article, please get in contact with embomolmed@wiley.com as early as possible, in order to coordinate publication and release dates.

LICENSE AND PAYMENT:

All articles published in EMBO Molecular Medicine are fully open access: immediately and freely available to read, download and share.

EMBO Molecular Medicine charges an article processing charge (APC) to cover the publication costs. You, as the corresponding author for this manuscript, should have already received a quote with the article processing fee separately. Please let us know in case this quote has not been received.

Once your article is at Wiley for editorial production you will receive an email from Wiley's Author Services system, which will ask you to log in and will present you with the publication license form for completion. Within the same system the publication fee can be paid by credit card, an invoice, pro forma invoice or purchase order can be requested.

Payment of the publication charge and the signed Open Access Agreement form must be received before the article can be published online.

PROOFS

You will receive the proofs by e-mail approximately 2 weeks after all relevant files have been sent o our Production Office. Please return them within 48 hours and if there should be any problems, please contact the production office at embopressproduction@wiley.com.

Please inform us if there is likely to be any difficulty in reaching you at the above address at that time. Failure to meet our

deadlines may result in a delay of publication.

All further communications concerning your paper proofs should quote reference number EMM-2022-16732-V3 and be directed to the production office at embopressproduction@wiley.com.

EMBO Press Author Checklist

Corresponding Author Name: Patrick Mehlen and Benjamin Gibert

Journal Submitted to: EMBO Molecular Medicine
Manuscript Number: EMM-2022-16732

USEFUL LINKS FOR COMPLETING THIS FORM
The EMBO Journal - Author Guidelines
EMBO Reports - Author Guidelines
Molecular Systems Biology - Author Guidelines
EMBO Molecular Medicine - Author Guidelines

Reporting Checklist for Life Science Articles (updated January 2022)
This checklist is adapted from Materials Design Analysis Reporting (MDAR) Checklist for Authors. MDAR establishes a minimum set of requirements in transparent reporting in the life sciences (see Statement of Task: 10.31222/osf.io/9sm4x). Please follow the journal's guidelines in preparing your manuscript.
Please note that a copy of this checklist will be published alongside your article.

Abridged guidelines for figures
1. Data
The data shown in figures should satisfy the following conditions:
- the data were obtained and processed according to the field's best practice and are presented to reflect the results of the experiments in an accurate and unbiased manner.
- ideally, figure panels should include only measurements that are directly comparable to each other and obtained with the same assay.
- plots include clearly labeled error bars for independent experiments and sample sizes. Unless justified, error bars should not be shown for technical replicates.
- if n<5, the individual data points from each experiment should be plotted. Any statistical test employed should be justified.
- Source Data should be included to report the data underlying figures according to the guidelines set out in the authorship guidelines on Data Presentation.

2. Captions
Each figure caption should contain the following information, for each panel where they are relevant:
- a specification of the experimental system investigated (eg cell line, species name).
- the assay(s) and method(s) used to carry out the reported observations and measurements.
- an explicit mention of the biological and chemical entity(ies) that are being measured.
- an explicit mention of the biological and chemical entity(ies) that are altered/varied/perturbed in a controlled manner.
- the exact sample size (n) for each experimental group/condition, given as a number, not a range;
- a description of the sample collection allowing the reader to understand whether the samples represent technical or biological replicates (including how many animals, litters, cultures, etc.).
- a statement of how many times the experiment shown was independently replicated in the laboratory.
- definitions of statistical methods and measures:
  - common tests, such as t-test (please specify whether paired vs. unpaired), simple χ2 tests, Wilcoxon and Mann-Whitney tests, can be unambiguously identified by name only, but more complex techniques should be described in the methods section;
  - are tests one-sided or two-sided?
  - are there adjustments for multiple comparisons?
  - exact statistical test results, e.g., P values = x but not P values < x;
  - definition of 'center values' as median or average;
  - definition of error bars as s.d. or s.e.m.

Please complete ALL of the questions below.
Select "Not Applicable" only when the requested information is not relevant for your study.

Materials

Newly Created Materials	Information included in the manuscript?	In which section is the information available? (Reagents and Tools Table, Materials and Methods, Figures, Data Availability Section)
New materials and reagents need to be available; do any restrictions apply?	Yes	All molecules can be requested directly from the laboratories.

Antibodies	Information included in the manuscript?	In which section is the information available? (Reagents and Tools Table, Materials and Methods, Figures, Data Availability Section)
For **antibodies** provide the following information:<br>- Commercial antibodies: RRID (if possible) or supplier name, catalogue number and or/clone number<br>- Non-commercial: RRID or citation	Yes	Material and Methods

DNA and RNA sequences	Information included in the manuscript?	In which section is the information available? (Reagents and Tools Table, Materials and Methods, Figures, Data Availability Section)
**Short novel DNA or RNA including primers, probes**: provide the sequences.	Not Applicable	

Cell materials	Information included in the manuscript?	In which section is the information available? (Reagents and Tools Table, Materials and Methods, Figures, Data Availability Section)
**Cell lines:** Provide species information, strain. Provide accession number in repository **OR** supplier name, catalog number, clone number, and/**OR** RRID.	Yes	Material and Methods
**Primary cultures:** Provide species, strain, sex of origin, genetic modification status.	Not Applicable	
Report if the cell lines were recently **authenticated** (e.g., by STR profiling) and tested for mycoplasma contamination.	Yes	Tests for mycoplama infection were realized

Experimental animals	Information included in the manuscript?	In which section is the information available? (Reagents and Tools Table, Materials and Methods, Figures, Data Availability Section)
**Laboratory animals or Model organisms:** Provide species, strain, sex, age, genetic modification status. Provide accession number in repository **OR** supplier name, catalog number, clone number, **OR** RRID.	Yes	Material and methods and main text
**Animal observed in or captured from the field:** Provide species, sex, and age where possible.	Not Applicable	
Please detail **housing and husbandry conditions**.	Yes	Material and methods section

Plants and microbes	Information included in the manuscript?	In which section is the information available? (Reagents and Tools Table, Materials and Methods, Figures, Data Availability Section)
**Plants:** provide species and strain, ecotype and cultivar where relevant, unique accession number if available, and source (including location for collected wild specimens).	Not Applicable	
**Microbes:** provide species and strain, unique accession number if available, and source.	Not Applicable	

Human research participants	Information included in the manuscript?	In which section is the information available? (Reagents and Tools Table, Materials and Methods, Figures, Data Availability Section)
If collected and within the bounds of privacy constraints report on age, sex and gender or ethnicity for all study participants.	Not Applicable	

Core facilities	Information included in the manuscript?	In which section is the information available? (Reagents and Tools Table, Materials and Methods, Figures, Data Availability Section)
If your work benefited from core facilities, was their service mentioned in the acknowledgments section?	Yes	Material and methods

Design

Study protocol	Information included in the manuscript?	In which section is the information available? (Reagents and Tools Table, Materials and Methods, Figures, Data Availability Section)
If study protocol has been **pre-registered, provide DOI in the manuscript**. For clinical trials, provide the trial registration number **OR** cite DOI.	Not Applicable	
Report the **clinical trial registration number** (at ClinicalTrials.gov or equivalent), where applicable.	Not Applicable	

Laboratory protocol	Information included in the manuscript?	In which section is the information available? (Reagents and Tools Table, Materials and Methods, Figures, Data Availability Section)
Provide DOI OR other citation details if **external detailed step-by-step protocols** are available.	Not Applicable	

Experimental study design and statistics	Information included in the manuscript?	In which section is the information available? (Reagents and Tools Table, Materials and Methods, Figures, Data Availability Section)
Include a statement about **sample size** estimate even if no statistical methods were used.	Yes	Main text, Materail and Methods section and Figure legends
Were any steps taken to minimize the effects of subjective bias when allocating animals/samples to treatment (e.g. **randomization procedure**)? If yes, have they been described?	Yes	A randomization was performed to homogeize mice around 100mm3 for the tratements with NP137-177Lu. The same randomization was performed for imagery analyses when tumors reached 400 to 800mm3
Include a statement about **blinding** even if no blinding was done.	Yes	The experiments were not blinded
Describe **inclusion/exclusion criteria** if samples or animals were excluded from the analysis. Were the criteria pre-established?<br><br>If sample or data points were omitted from analysis, report if this was due to attrition or intentional exclusion and provide justification.	Yes	No animals were exclued form the analysis
For every figure, are **statistical tests** justified as appropriate? Do the data meet the assumptions of the tests (e.g., normal distribution)? Describe any methods used to assess it. Is there an estimate of variation within each group of data? Is the variance similar between the groups that are being statistically compared?	Yes	All statistical test are justified in the materail and methods, th main text or figures legends

Sample definition and in-laboratory replication	Information included in the manuscript?	In which section is the information available? (Reagents and Tools Table, Materials and Methods, Figures, Data Availability Section)
In the figure legends: state number of times the experiment was **replicated** in laboratory.	Yes	Figure legends
In the figure legends: define whether data describe **technical or biological replicates**.	Yes	All test are biological replicates.

Ethics

Ethics	Information included in the manuscript?	In which section is the information available? (Reagents and Tools Table, Materials and Methods, Figures, Data Availability Section)
Studies involving **human participants**: State details of **authority granting ethics approval** (IRB or equivalent committee(s), provide reference number for approval.	Yes	The usage of all patient tumor samples was conducted according to the rules of WMA Declaration of Helsinki, the Belmont Report and the Ethic Committee of the Medical Board (CHU Lyon) agreed to these investigations. Informed consent was obtained from all patients. The collection of samples has been realized on primary tumors from patients treated at the Hospices civils de Lyon and specimens are conserved in its pathology department.
Studies involving **human participants**: Include a statement confirming that **informed consent** was obtained from all subjects and that the experiments conformed to the principles set out in the WMA Declaration of Helsinki and the Department of Health and Human Services Belmont Report.	Yes	A statement was added in the Material and methods section accordingly.
Studies involving **human participants:** For publication of **patient photos**, include a statement confirming that consent to publish was obtained.	Not Applicable	
Studies involving experimental **animals**: State details of **authority granting ethics approval** (IRB or equivalent committee(s), provide reference number for approval. Include a statement of compliance with ethical regulations.	Yes	Their care and accommodation were in accordance with European and French institutional guidelines as defined by the local CECCAP Ethics Committee (CECCAPP_CLB_2020_024)
Studies involving **specimen and field samples:** State if relevant **permits** obtained, provide details of authority approving study; if none were required, explain why.	Not Applicable	

Dual Use Research of Concern (DURC)	Information included in the manuscript?	In which section is the information available? (Reagents and Tools Table, Materials and Methods, Figures, Data Availability Section)
Could your study fall under dual use research restrictions? Please check biosecurity documents and list of **select agents and toxins** (CDC): https://www.selectagents.gov/sat/list.htm	Not Applicable	
If you used a select agent, is the security level of the lab appropriate and reported in the manuscript?	Not Applicable	
If a study is subject to dual use research of concern regulations, is the name of the **authority granting approval and reference number** for the regulatory approval provided in the manuscript?	Not Applicable	

Reporting

The MDAR framework recommends adoption of discipline-specific guidelines, established and endorsed through community initiatives. Journals have their own policy about requiring specific guidelines and recommendations to complement MDAR.

Adherence to community standards	Information included in the manuscript?	In which section is the information available? (Reagents and Tools Table, Materials and Methods, Figures, Data Availability Section)
State if relevant guidelines or checklists (e.g., **ICMJE, MIBBI, ARRIVE, PRISMA**) have been followed or provided.	Not Applicable	
For **tumor marker prognostic studies,** we recommend that you follow the **REMARK** reporting guidelines (see link list at top right). See author guidelines, under 'Reporting Guidelines'. Please confirm you have followed these guidelines.	Not Applicable	
For **phase II and III randomized controlled trials**, please refer to the **CONSORT** flow diagram (see link list at top right) and submit the CONSORT checklist (see link list at top right) with your submission. See author guidelines, under 'Reporting Guidelines'. Please confirm you have submitted this list.	Not Applicable	

Data Availability

Data availability	Information included in the manuscript?	In which section is the information available? (Reagents and Tools Table, Materials and Methods, Figures, Data Availability Section)
Have **primary datasets** been deposited according to the journal's guidelines (see 'Data Deposition' section) and the respective accession numbers provided in the Data Availability Section?	Yes	This study includes no data deposited in external repositories
Were **human clinical and genomic datasets** deposited in a public access-controlled repository in accordance to ethical obligations to the patients and to the applicable consent agreement?	Not Applicable	
Are **computational models** that are central and integral to a study available without restrictions in a machine-readable form? Were the relevant accession numbers or links provided?	Not Applicable	
If publicly available data were reused, provide the respective **data citations in the reference list**.	Not Applicable	
